# IBD sharing patterns as intra-breed admixture indicators in small ruminants

**DOI:** 10.1038/s41437-023-00658-x

**Published:** 2023-11-03

**Authors:** Stéphane Blondeau Da Silva, Joram M. Mwacharo, Menghua Li, Abulgasim Ahbara, Farai Catherine Muchadeyi, Edgar Farai Dzomba, Johannes A. Lenstra, Anne Da Silva

**Affiliations:** 1 Rectorat Limoges, 87000 Limoges, France; 2 Animal and Veterinary Sciences, Scotlands Rural College (SRUC) and Centre for Tropical Livestock Genetics and Health (CTLGH), The Roslin Institute Building, EH25 9RG Midlothian, UK; 3 Small Ruminant Genomics, International Centre for Agricultural Research in the Dry Areas (ICARDA), P.O. Box 5689, Addis Ababa, Ethiopia; 4 College of Animal Science and Technology, China Agricultural University, Beijing, 100193 China; 5 Department of Zoology, Faculty of Sciences, Misurata University, Misurata, Libya; 6 Agricultural Research Council, Biotechnology Platform, Onderstepoort, 0110 South Africa; 7 Discipline of Genetics, School of Life Sciences, University of KwaZulu-Natal, Private Bag X01, Scottsville, 3209 South Africa; 8 Faculty of Veterinary Medicine, Utrecht University, Utrecht, Netherlands; 9 Faculté des Sciences et Techniques de Limoges, E2LIM, 87000 Limoges, France

**Keywords:** Animal breeding, Haplotypes

## Abstract

In this study, we investigated how IBD patterns shared between individuals of the same breed could be informative of its admixture level, with the underlying assumption that the most admixed breeds, i.e. the least genetically isolated, should have a much more fragmented genome. We considered 111 goat breeds (i.e. 2501 individuals) and 156 sheep breeds (i.e. 3304 individuals) from Europe, Africa and Asia, for which beadchip SNP genotypes had been performed. We inferred the breed’s level of admixture from: (i) the proportion of the genome shared by breed’s members (i.e. “genetic integrity level” assessed from ADMIXTURE software analyses), and (ii) the “AV index” (calculated from Reynolds’ genetic distances), used as a proxy for the “genetic distinctiveness”. In both goat and sheep datasets, the statistical analyses (comparison of means, Spearman correlations, LM and GAM models) revealed that the most genetically isolated breeds, also showed IBD profiles made up of more shared IBD segments, which were also longer. These results pave the way for further research that could lead to the development of admixture indicators, based on the characterization of intra-breed shared IBD segments, particularly effective as they would be independent of the knowledge of the whole genetic landscape in which the breeds evolve. Finally, by highlighting the fragmentation experienced by the genomes subjected to crossbreeding carried out over the last few generations, the study reminds us of the need to preserve local breeds and the integrity of their adaptive architectures that have been shaped over the centuries.

## Introduction

Whereas livestock diversity is essential for food security, the Food and Agriculture Organization of the United Nations’s (FAO) predicts the loss of one breed per month (FAO 2015). In ruminants in particular, local breeds are largely abandoned in favor of more productive ones, or are subject to unsupervised crossbreeding in order to up-grade them (Shrestha 2005). This crossing with more profitable, often exotic breeds, represents one of the major threats to the diversity of the resource, and is largely found in developing countries where state structures are less able to monitor the practices of breeders, under increasing economic pressure (Scherf et al. 2006; Boutrais 2007). Indiscriminate crossbreeding has led to a widespread loss of the original/parent indigenous breeds and to the formation of nondescript crossbreds (Bett et al. 2013), mostly incompatible with local production systems (Okeno et al. 2013), and characterized by a genetic composition probably unstable over time (Paim et al. 2020). These crossbreeding practices favor genetic homogenization (Ouchene-Khelifi et al. 2018; Belabdi et al. 2019) via the loss of rare or specific variants, and induce disruption of co-adapted gene complexes, to the detriment of the integrity and the viability of locally adapted populations (Todesco et al. 2016; Zhang et al. 2019; Ågren et al. 2019).

Several software programs, assessing the genetic structure of populations, can be used to identify crossbreeding practices, by estimating individual ancestry from independent multilocus SNP genotype datasets, i.e. STRUCTURE (Lawson et al. 2012), ADMIXTURE (Alexander and Novembre 2009), PCAdapt (Luu et al. 2017), etc. Whatever the underlying algorithms, the different programs are designed to analyze the differences in the distribution of genetic variants between populations. Hence, these methods imply that if a population (A) is intensively crossed with a population (B) that does not appear in the dataset, and if (A) and (B) have no genetic interaction with the other populations of the dataset, then (A) will appear as not admixed. In developing countries, it can be very challenging to build datasets that are representative of the complexity of field practices. Indeed, the ideal working conditions would imply significant financial means, allowing to palliate the difficulties linked to the sometimes extreme climatic conditions, to the complex geography, to the political tensions, etc., and especially the ability to collaborate with local specialists of the breeding issue. As it is precisely in developing countries that it is essential to detect crossbreeding practices affecting the indigenous breeds (FAO 2007, 2015) particularly adapted to local conditions (environment and management) (Hoffmann 2013), it seems of interest to find an admixture indicator that could remain effective even in the absence of the breeds, involved in the crosses, within the dataset considered.

A haplotype, as a particular combination of alleles on the same chromosome inherited together, captures Linkage Disequilibrium (LD) among genotypes at linked loci. It constitutes a rich but largely underexploited information in the field of conservation genomics, especially since haplotype data could enhance our understanding of genetic admixture (Leitwein et al. 2020). From Fisher (1949, 1954), several scientists including Barton and Bengtsson (1986), Baird et al. (2003), Janzen et al. (2018), have been interested in partitioning admixed individual’s genomes into blocks originating from different ancestral populations. Indeed, crossbreeding and more generally “hybridization” leads to introgression of migrant chromosomes into a receiving genetic background. The lengths of the “migrant tracts” or admixture “chunks” (Falush et al. 2003) will become progressively shorter over generations (Pool and Nielsen 2009). The admixed genomes can be depicted as a mosaic of local ancestry tracts from differentiated populations (Liang and Nielsen 2014). Hybridization induces the fragmentation of the genomic blocks of contiguous ancestry through genetic recombination, which leads to an exchange of genetic material between homologous chromosomes. The breakdown of ancestral haploblocks (i.e. contiguous ancestry blocks), via the introduction of distinct haplotypes will increase the occurrence of mosaics in the genome (Freedman et al. 2016; Aliloo et al. 2020) and reduce the length of Identity-By-Decent (IBD) tracts (Leutenegger et al. 2003).

IBD refers to identical DNA segments inherited from a recent common ancestor without recombination, i.e. IBD is the shared inheritance of an identical portion of the genome between two individuals, providing direct evidence of genetic relatedness. This differs from identity by state (IBS), in which part of the genomes of two individuals may appear to be identical, but not necessarily as a result of recent common inheritance (Browning and Browning 2012). The length and proportion of shared IBD segments serve as an indicator of the age of the most recent common ancestor. The principle underlying is that long haplotypes shared between individuals are more likely, statistically speaking, to be the consequence of a kinship due to a common, deep-rooted history of the population rather than to random recombination or mutation (Browning 2008). The issue of detecting identical segments by descent (IBD) has aroused renewed interest, as it offers unprecedented possibilities for the study of population history and genealogy (Tang et al. 2022). Indeed, patterns of IBD segment sharing between groups of individuals in the same population reveal the population demographic history, recent effective population size and rates of migration. In particular, IBD segments have been used to detect introgression in both animals (Bosse et al. 2014) and plants (Ferdy and Austerlitz 2002).

In this study, we exploit the availability of large SNP genotyped datasets in goats and sheep to investigate the relationship between admixture, i.e. gene flow between populations, and patterns of IBD-sharing, in breeds of small ruminants. We inferred admixture level from (i) the proportion of the genome shared by breed’s members (“genetic integrity level”), obtained with the ADMIXTURE software, and (ii) the “AV index”, calculated from Reynold’s genetic distances (i.e. distances derived from Wright fixation index, Wright 1965) to identify the level of “genetic originality” of each breed in a dataset. The hypothesis was that admixed breeds, i.e. breeds that are the least isolated from a genetic point of view, should have a much more fragmented genome and thus exhibit shorter and fewer shared IBD segments. In this case, the characterization of intra-breed shared IBD segments could be used as an indicator of admixture level, which would not imply the knowledge of the whole genetic landscape in which the breed evolves. It would be extremely valuable in detecting efficiently and quickly the breeds in danger due to crossbreeding.

## Material and methods

### Datasets

We used the AdaptMap dataset, including goat breeds from Europe (40), Asia (19) and Africa (52) (see details in Table 1 and Supplementary Table 1), genotyped with the Caprine SNP50 BeadChip (Bertolini et al. 2018, available via Dryad: 10.5061/dryad.v8g21pt), for 53,547 SNPs. For sheep, we analyzed European (73), Asian (50) and African (33) breeds (see details in Table 2 and Supplementary Table 2). We used the following datasets : (i) French breeds obtained with the Illumina Ovine HD SNP Beadchip (Rochus et al. 2018, Zenodo repository 10.5281/zenodo.237116); (ii) Italian and Spanish breeds (Ciani et al. 2020, available at 10.23644/uu.8947346); (iii) Asian and North/Central European breeds (Kijas et al. 2012, available at http://www.sheephapmap.org); (iv) Chinese breeds (Zhao et al. 2017); (v) Ethiopian breeds (Edea et al. 2017, available at www.animalgenome.org/repository/pub/KORE2017.1122; Ahbara et al. 2019, available at www.animalgenome.org/repository/pub/NOTT2018.0423; Amane et al. 2020) and (vi) South African sheep breeds (Dzomba et al. 2020, available at osf.io/ceup6/?view_only=a1959659de5f4d5d9bbb1c607b2d83b6; Molotsi et al. 2017). SNP data from French breeds were extracted from the 600 K variation using the Ovine SNP50 BeadChip coordinates of SNPs on the OAR v3.1 reference genome assembly using Vcftools (Danecek et al. 2011). The other sheep datasets were obtained with the Illumina Ovine SNP50 BeadChip. SNPs and animals were pruned with PLINK v1.07 (Purcell et al. 2007) using the following filtering thresholds: (i) SNP call rate ≤ 97%; (ii) SNP minor allele frequency (MAF) ≤ 1%; (iii) animals displaying ≥ 10% of missing genotypes. After filtration of the merged datasets, we retained 50,220 genotypes for 2501 goats and 39,893 genotypes for 3304 sheep. The R-package Hierfstat (Goudet 2005) was used to estimate F_IS_ (mean and 95% confidence interval) by population.

**Table 1:** Characterization of the goat datasets in terms of admixture and shared IBD segments.

Dataset origin (number of breeds considered)	Proportion of “admixed” (Q < 0.85) breeds (“genetic integrity level”: mean; s.d.)	Mean length of IBD for “admixed” breeds - “slightly admixed” breeds (s.d.) Mb	Mean number of IBD for “admixed” breeds – “slightly admixed” breeds (s.d.)	Spearman correlations
**Europe**				
South European dataset (32)	67.65% (0.71; 0.17)	5.71 (4.00) - 10.37 (1.73)***	9.81 (12.00) - 22.51 (18.07)*	
North Europe (8)	12.5% (0.91; 0.19)	2.15 (single value) - 11.26 (2.65)	9.82 (single value) - 55.08 (38.18)	
All European datasets (40)	60% (0.75; 0.19)	5.56 (3.98) - 10.76 (2.15)***	9.81 (11.73) - 36.76 (32.18)**	integrity level/IBD No: 0.52*** integrity level/IBD size: 0.63** AV/IBD No: 0.77*** AV/IBD size: 0.71***
**Asian dataset** (19)	84.21% (0.65; 0.22)	7.06 (4.75) - 14.74 (2.55) nt	12.55 (12.54) - 53.42 (21.82) nt	integrity level/IBD No: 0.76*** integrity level/IBD size: 0.77*** AV/IBD No: 0.90 *** AV/IBD size: 0.84***
**Africa**				
East Africa (33)	70% (0.71; 0.20)	2.42 (3.21) - 6.46 (4.50)**	4.27 (9.02) - 16.44 (15.52)*	
North and West Africa (19)	94.44% (0.57; 0.20)	2.87 (3.13) - 12.37 (single value)	1.03 (3.32) - 11.66 (single value)	
All African datasets (52)	78.43% (0.66;0.21)	1.89 (3.09) - 6.99 (4.63)***	2.89 (7.28) - 16.00 (14.79)*	integrity level/IBD No: 0.37** integrity level/IBD size: 0.39** AV/IBD No: 0.68 *** AV/IBD size: 0.67***
**All datasets (111)**	72.30% (0.69; 0.21)	3.98 (4.29) – 9.78 (4.03)***	6.82 (10.61) – 30.82 (28.28)***	integrity level/IBD No: 0.50 *** integrity level/IBD size: 0.52 *** AV/IBD No: 0.83 *** AV/IBD size: 0.77 ***

Statistical comparison of the mean “length” and mean “number” of IBD shared segments between “admixed” and “slightly admixed” breeds, and Spearman correlations between “AV index” or “genetic integrity level” and the “length” or “number of IBD shared”. *s.d.* standard deviation, *No* number, ns *p* value > 0.1, •0.1 < *p* value > 0.05, *p value ≤ 0.05, **p value ≤ 0.01, ***p value ≤ 0.001, *nt* not tested, due to size imbalance between the two groups.

**Table 2:** Characterization of the sheep datasets in terms of admixture and shared IBD segments.

Dataset origin (number of breeds considered)	Proportion of “admixed” (Q < 0.85) breeds (“genetic integrity level”: mean; s.d.)	Mean length of IBD for “admixed” breeds - “slightly admixed” breeds (s.d.) Mb	Mean number of IBD for “admixed” breeds – “slightly admixed” breeds (s.d.)	Spearman correlations
**Europe**				
France (25)	28% (0.90; 0.14)	6.73 (2.17) - 10.95 (1.51)***	2.10 (1.76) - 17.47 (20.61)**	
Italy (18)	55.55% (0.77; 0.17)	5.44 (3.91) - 10.79 (3.24)**	3.58 (2.93) - 10.28 (5.73)*	
Spain (10)	100% (0.57; 0.21)	3.08 (2.03) - none	1.13 (0.75) - none	
Central Europe (10)	40% (0.83; 0.15)	16.56 (5.27) - 11.89 (3.36) nt	11.77 (2.32) - 11.88 (6.97) nt	
Northern Europe (10)	20% (0.91; 0.08)	17.61 (8.42) - 15.61 (3.78) nt	5.70 (1.08) - 15.97 (10.83) nt	
All European datasets (73)	45.20% (0.81; 0.20)	3.64 (3.84) – 14.89 (15.08)***	7.08 (5.94) – 11.99 (3.22)***	integrity level/IBD No: 0.63 *** integrity level/IBD size: 0.52*** AV/IBD No: 0.73 *** AV/IBD size: 0.82***
**Asia**				
West Asia (7)	71% (0.76; 0.14)	4.51 (3.28) - 7.79 (1.07) nt	3.09 (2.54) - 14.84 (0.87) nt	
East Asia (8)	50% (0.87; 0.07)	1.76 (1.51) - 4.73 (2.02) nt	1.46 (1.31) - 5.99 (2.75) nt	
China (35)	82.86% (0.57;0.23)	4.49 (4.19) - 9.34 (2.81) *	5.14 (13.83) - 16.77 (11.26) **•**	
All Asian datasets (50)	76% (0.64; 0.23)	4.20 (3.96) - 7.54 (3.15)*	4.48 (12.14) - 12.85 (9.31)*	integrity level/IBD No: 0.62 *** integrity level/IBD size: 0.53*** AV/IBD No: 0.71 *** AV/IBD size: 0.56***
**Africa**				
Ethiopia (19)	89.47% (0.62;0.20)	1.06 (1.16) - 0 (0) nt	0.673 (1.04) - 0 (0) nt	
South Africa (14)	64.28 (0.69; 0.22)	11.22 (3.65) - 14.78 (7.00) nt	9.91 (3.41) - 39.18 (11.39) nt	
All African datasets (33)	78.80% (0.65; 0.21)	4.58 (5.42) – 10.56 (9.20) ns	3.86 (4.94) – 27.98 (21.26)*	integrity level/IBD No: 0.11 ns integrity level/IBD size: −0.05 ns AV/IBD No: 0.83 *** AV/IBD size: 0.79***
**All datasets (156)**	62.18% (0.72; 0.22)	5.28 (4.21) - 10.92 (4.56) ***	4.03 (8.26) - 16.03 (15.37) ***	integrity level/IBD No: 0.57 *** integrity level/IBD size: 0.53 *** AV/IBD No: 0.76 *** AV/IBD size: 0.76 ***

Statistical comparison of the mean length and mean number of IBD shared segments between “admixed” and “slightly admixed” breeds, and Spearman correlations between “AV index” or “genetic integrity level” and the “length” or “number of IBD shared”. *s.d.* standard deviation, No number, ns *p* value > 0.1, •0.1 < *p* value > 0.05, *p value ≤ 0.05, **p value ≤ 0.01, ***p value ≤ 0.001, *nt* not tested, due to size imbalance between the two groups.

### IBD sharing pattern

BEAGLE 4.1 (Browning and Browning 2007) was used to detect IBD segments that define haplotypes originating from a recent and single common ancestor. When detecting an IBD segment, it must be sufficiently long to ensure that it is not an aggregate of several short IBD segments from different ancient common ancestors. The first part of the algorithm, based on probabilistic methods, is focused on data phasing. In a second step, the phased haplotypes are used to build a haplotype frequency model and, for each shared candidate IBD segment, the LOD score, which is the log (base 10) of the likelihood ratio, is calculated. Candidate segments whose LOD score is above a specified threshold are flagged as IBD segments. Segments with a LOD score <4 and a length shorter than 0.5 cM were excluded (knowing that Beagle assumes a constant recombination rate of 1 cM per Mb), and the ibdtrim parameter was set to 40, according to recommendation of Browning and Browning (2007). We used in-house python 3 (Van Rossum and Drake 1995) script (available on request) to obtain the mean length and number of IBD segments shared by individuals of the same breed.

### ADMIXTURE analyses

The approaches most frequently used to describe population structure are principal component analysis (Patterson et al. 2006) and admixture proportion inference (Lawson et al. 2012; Alexander and Novembre 2009). Whereas principal component analysis reduces a multidimensional dataset to a much more restricted number of dimensions, with admixture proportion inference, individuals in a sample are modelized as having a fraction of their genome deriving from each of several source populations. The basic assumption of this model is that individuals are members of a set of K discrete groups, each with a specific allele frequency, knowing that individuals can belong fractionally to each group. The aim is to infer the proportions of ancestry in each source population. This model is particularly well suited to the analysis of ruminant breeds, which have developed over time to form populations with specific evolutionary histories linked to a given territory and according to the rules of a pastoral social group (Serranito et al. 2021a). These patterns of genetic uniqueness have been disrupted over the last few decades by agricultural intensification, sometimes leading to recent admixtures, which are detected with great relevance by these approaches, making them widely used (Decker et al. 2014; Upadhyay et al. 2019). ADMIXTURE software is a clustering-based approach for maximum likelihood estimation of individual ancestry, which analyses a dataset of independent multilocus SNP genotypes (Alexander et al. 2009). The first step before using the software is, hence, to filter SNPs on the basis of pairwise LD to produce a reduced set of more independent markers. SNP pruning was carried out using the –indep option of PLINK with the following parameters: 50 SNPs per window, a shift of five SNPs between windows, and a variation inflation factor’s threshold of two (corresponding to R^2^ > 0.5). ADMIXTURE was run with K = 2 to x, with x corresponding to the total number of breeds considered in the dataset. For each value of K, 10 independent runs were performed. The program CLUMPAK (Kopelman et al. 2015) was used to analyze the multiple independent runs at a single K and visualize the results. For K = x (with x corresponding to the total number of breeds considered in the dataset), the proportion of membership for each predefined breed in each cluster was annotated. These results allowed defining two groups: (i) “admixed” breeds were defined as breeds sharing less than 85% of their genome with all the individuals of the breeds in the majority cluster (Q ≤ 0.85), and breeds were classified as (ii) “slightly admixed” if more than 85% of their genome was shared by all members of the breed for the main cluster (Q > 0.85). The value of 0.85 corresponds approximately to the third quartile of the sheep and goat distributions for the “genetic integrity level” variable (defined below); it was thus chosen as a threshold favoring statistically comparable datasets between the “admixed” and “slightly admixed” groups. We also characterized each breed by a “genetic integrity level” defined as the membership proportion for the main cluster (i.e. Q-value of the main cluster). The PCAdapt R package (Privé et al. 2020) was used to perform principal component analysis (PCA) to visualize the variability available within the different datasets (Supplementary Figures 1-2).

### Breed “originality” index

Pavoine et al. (2005) have adapted methods initially developed from phylogenetic trees to measure the “originality” of species and populations. From a biodiversity perspective, “originality” (i.e. distinctiveness or isolation) represents the total contribution of the species or population to the biodiversity of the whole (Pavoine et al. 2005). We used the R package ADIV, to quantify the originality of the breeds in the different datasets, or in other words the strength of the genetic flow linking them to other breeds, via dissimilarity indices. Phylogenetic dissimilarities can be calculated, for example as the sum of the branch lengths of the shortest path linking the two groups considered on the tree (Pavoine et al. 2017). The AV index, for “average”, obtained by the “distinctDis” function, was retained, representing the average dissimilarity between a focal breed and all the others (Eiswerth and Haney 1992; Ricotta 2004). This index was calculated from a pairwise matrix based on Reynolds’ genetic distances (Reynolds et al. 1983), which was used as the dissimilarity matrix. Higher values of AV (towards 1) indicate greater originality, i.e. greater genetic isolation from other breeds, or genetic specificity, while values close to zero indicate strong genetic flow between breeds. NeighborNet graphs based on Reynolds’ genetic distances were constructed using SplitsTree (Huson and Bryant 2006), to visualize the “originality” of breeds within the different datasets, through branch length and arrangement (see Supplementary Figures 3-4).

### Statistical analyses

We used Linear Model (LM) and Generalized Additive Model (GAM) to study the weight of IBD variables (number and length) in the prediction of “AV index” and “genetic integrity level”. The different models were compared in terms of performance with chi-square tests. GAM is a nonparametric extension of the generalized linear model, which can deal directly with nonlinear relationships between response variables and multiple explanatory variables. We use the MGCV R-package to construct and test the models (Pedersen et al. 2018; Wood 2011). The adjusted R^2^, defined as the proportion of variance explained by the model, was used as an indicator of the model’s relevance.

Student’s t-tests were used to compare mean IBD segment lengths and numbers in the “admixed” and “slightly admixed” groups, i.e. according to the “admixture status”. Spearman’s correlation coefficient was used to assess the relationship between the number or length of IBD segments and the “genetic integrity level” or the “AV index”. To compare characteristics (mean “genetic integrity levels”, mean “AV index”, etc.) of the different datasets (African, Asian, and European) ANOVA tests were performed followed by post-hoc Tukey’s tests (alpha = 0.05).

We used chromoMap (Anand and Rodriguez Lopez 2022), an R package, to visualize and map chromosomal feature with known coordinates. The “aggregate_func” argument was parameterized with the “sum” function to represent the set of haplotype blocks shared between pairs of individuals. The software R 4.0.5 (R Core Team 2022) was used for the analyses, with the ggplot2 (Wickham 2016) package, for graphic representations.

## Results

### Datasets characteristics

For goats, we analyzed 111 breeds via 2501 individuals, including 40 breeds from Europe, 52 from Africa and 19 from Asia. For sheep, we considered 156 breeds, via 3304 individuals, including 74 breeds from Europe, 33 breeds from Africa and 49 from Asia. We randomly kept only 30 individuals, for breeds with a large number of individuals, and we removed breeds represented by less than eight individuals (to ensure accuracy of the results, Browning and Browning 2007). On average we considered 22.53 individuals per breed (s.d. = 6.42) for goats and 21.18 individuals per breed (s.d. = 5.35) for sheep (see details in Supplementary Tables 1-2). For goats, the mean F_IS_ was 0.016 (s.d. = 0.036) and F_IS_ per population ranged from −0.076 to 0.173. The Irish breed, Old Irish goat cross (OIGX) and the Thyolo breed from Malawi (THY), were the only breeds showing F_IS_ values above 0.10, respectively at 0.173 and 0.112. For sheep, the mean F_IS_ was 0.015 (s.d. = 0.039) and F_IS_ by population ranged from −0.092 to 0.198. The South African Dorper breed (DOP, F_IS_ = 0.126), the Asian Bengladeshi breed (BGE, F_IS_ = 0.147), and the French Ouessant breed (OUE, F_IS_ = 0.177) showed F_IS_ values above 0.1 (Supplementary Table 3-4).

### Links between admixture levels and IBD sharing patterns

Taking all datasets together, 72.30 and 62.18% of goat and sheep breeds, respectively, were classified as “admixed” (Tables 1, 2, details in Supplementary Tables 1-2 and Supplementary Figures 1-2). It is noteworthy that all the Spanish sheep breeds and 95% of the North and West African goat breeds were classified as “admixed”.

For goats (Table 1), the statistical tests revealed no significant difference between the average “genetic integrity level” of European and Asian breeds, but a trend towards higher genetic integrity level for European breeds (mean = 0.75, s.d. = 0.19) than for African breeds (mean = 0.66, s.d. = 0.21), was detected (*p* value = 0.07). For sheep datasets (Table 2), the average integrity level was higher for European breeds (0.81, s.d. = 0.20) than for Asian or African breeds (mean around 0.65, s.d. around 0.20), i.e. *p* values < 0.01 for ANOVA and Tukey post hoc tests.

For goats the mean “AV index” was 0.092 (s.d. = 0.05) and ranged from 0.028 to 0.353. European breeds showed significantly higher mean AV value (mean = 0.11, s.d = 0.06) than African breeds (mean = 0.07, s.d. = 0.04), i.e. *p* values < 0.001 for ANOVA and Tukey post hoc tests. For sheep the mean “AV index” was 0.097 (s.d. = 0.06) and ranged from 0.017 to 0.293. European breeds showed significantly higher mean AV value (mean = 0.11, s.d. = 0.07) than Asian breeds (mean = 0.07, s.d. = 0.03), i.e. *p* values < 0.01 for ANOVA and Tukey post hoc tests (details in Supplementary Tables 1-2 and Supplementary Figures 3-4).

The average number of IBD segments per goat breed was 13.30 (s.d. = 19.91), and mean length was 5.4 Mb (s.d. = 4.91). The average number of IBD segments per sheep breed was 8.57 (s.d. = 12.84), and mean length was 7.41 Mb (s.d. = 5.67). For goats, African breeds showed significantly fewer IBD segments (mean = 5.62, s.d. = 10.64) than Asian (mean = 19.00, s.d. = 20.45) and European (mean = 20.59, s.d. = 25.66) breeds, i.e. *p* values < 0.01 for ANOVA and Tukey post hoc tests. The same observation applied to the length of the IBD segments (mean = 2.94, s.d. = 10.64 for African breeds, against mean = 8.27, s.d. = 20.45 for Asian breeds and mean = 7.64, s.d. = 25.66 for European breeds). For sheep, there was no significant difference in the average number of IBD segments between African, Asian and European breeds. On the other hand, European breeds showed significantly longer IBD segments (mean = 9.77, s.d. = 5.23) than Asian (mean = 5.00, s.d. = 4.02) and African breeds (mean = 5.85, s.d. = 6.71), i.e. *p* values < 0.001 for ANOVA and Tukey post hoc tests.

In goats, considering all the datasets together, the “admixed” breeds were characterized by fewer IBD segments (on average 6.82 versus 30.82 for the “slightly admixed” breeds, *p* value < 2.2×10^−16^), that were also shorter (on average 3.98 Mb versus 9.78 Mb for the “slightly admixed” breeds, *p* value = 3.66×10^−12^). In sheep, considering all the datasets together, the “admixed” breeds were characterized by fewer IBD segments (on average 4.03 versus 16.03 for the “slightly admixed” breeds, *p* value = 9.514×10^−5^), that were also shorter (on average 5.28 Mb versus 10.92 Mb for the “slightly admixed” breeds, *p* value = 9.514×10^−5^). These conclusions were drawn for all datasets tested individually, except for the number of IBD segments in African sheep (mean = 4.58, s.d. = 5.42 for “admixed breeds” versus, mean = 10.56 and s.d. = 9.20, for “slightly admixed” breeds, *p* value > 0.05) (Tables 1, 2 and Supplementary Figure 5).

Considering the goats and sheep datasets together (Tables 1, 2), the Spearman coefficient showed high correlations between the “AV index” and number or length of IBD segments (*ρ* around 0.75 in each case) ; correlation coefficient values were lower between “genetic integrity levels” and shared IBD segments number or length, *ρ* around 0.5 in each case (sheep and goats considered), but still highly significantly different from zero (Tables 1, 2). Considering the datasets independently, correlations between “genetic integrity levels” and number or length of IBD segments were weakest for African goat datasets (*ρ* around 0.37, *p* value < 0.001), and not significantly different from zero only for the African sheep datasets (Tables 1, 2).

### Modeling admixture from IBD sharing patterns

**Fig. 1:**
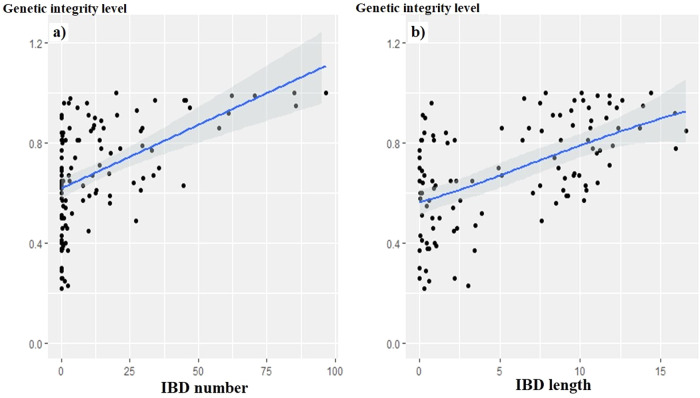
Plots of LM models with the variable to be predicted, “genetic integrity level” and the predictors. **a** The mean “number of shared IBD segments” (adjusted R^2^ = 0.24, *p* value < 0.0001) or (**b**) their mean “length” (in Mb, adjusted R^2^ = 0.31, *p* value < 0.0001)), for the Asian, African and European goat datasets considered together.

**Fig. 2:**
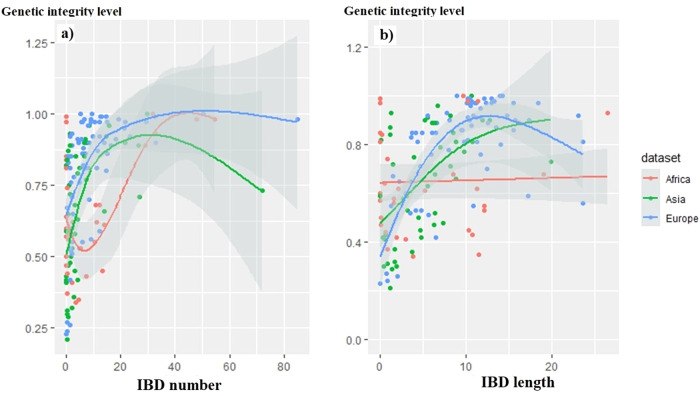
Plots of GAM models, with the variable to be predicted “genetic integrity level” and the predictors. **a** The mean “number of shared IBD segments” and (**b**) the mean “length of the shared IBD segments” (in Mb), taking into account the different sheep datasets (African, Asian and European), see details in Table 3.

Considering the “genetic integrity level” as the response (y) and the shared IBD segments “number” or “length” as predictors (x): (i) for goats, it appeared that LM models were more suitable than GAM models to fit the data. In addition, the models including the origin of the dataset (i.e. Africa, Asia or Europe) did not perform better than the basic LM models (*p* values > 0.05). The relationship (Fig. 1) between genetic integrity and the “number” (adjusted R^2^ = 0.236, *p* value < 0.0001) or “length” (adjusted R^2^ = 0.307, *p* value < 0.0001) of IBD segments was significant, but at low predictive values, there was a notable failure to adjust to the response, whereas at higher values, the correlation between predictor and response was clearer. (ii) For sheep, GAM models were better than LM models (*p* values < 0.0001). Table 3 gives details of the various GAM models tested, and Fig. 2 shows the relationships between “genetic integrity level” and shared IBD segments “number” and “length”, taking into account the origin of the datasets as it improved the models (adjusted R^2^ with “IBD segment number” as predictor = 0.43, adjusted R^2^ with “IBD segment length” as predictor = 0.45, *p* values < 0.001). The effective degrees of freedom (edf) estimated from GAM models can be used as an approximation of the degree of nonlinearity in predictor-response relationships. Indeed, an edf of 1 is equivalent to a linear relationship, an edf > 1 and ≤ 2 corresponds to a weakly non-linear relationship, while an edf > 2 indicates a strongly non-linear relationship (Zuur et al. 2009). Highly non-linear relationships are most likely to have inflection points and threshold responses. According to the edf values, the relationships were non-linear for all datasets. It appeared that as the value of the predictor increased, the value of “genetic integrity” increased, with a threshold zone beyond which there appeared a plateau or even a slight decrease. The African dataset showed the weakest relationship between “genetic integrity level” and “number” of IBD segments, with a convoluted curve and again for this dataset the relationship was not significant when considering “genetic integrity level” and IBD segment “length” as predictor (Table 3, Fig. 2).

**Table 3:** GAM models for sheep considering the “genetic integrity level”.

Sheep GAM Models for y = ”Genetic Integrity Level” (GIL)	Edf	*F* value (*p* value)	Adjusted R^2^
GIL ~ IBD number	IBD number: 3.70	14.84 ( < 0.0001)	0.30
GIL ~ IBD number*dataset origin	IBD number*Africa: 7.03 IBD number*Asia: 3.88 IBD number*Europe: 4.63	4.11 ( < 0.0001) 10.21 ( < 0.0001) 7.86 ( < 0.0001)	0.43
GIL ~ IBD length	IBD length: 6.29	11.14 ( < 0.0001)	0.34
GIL ~ IBD length*dataset origin	IBD length*Africa: 5.91 IBD length*Asia: 2.13 IBD length*Europe: 3.07	2.05 (0.06) 11.40 ( < 0.0001) 19.68 ( < 0.0001)	0.45

With the variable to be predicted “genetic integrity level” and the predictors, the mean “number of shared IBD segments” or the mean “length of the shared IBD segments”. *Edf* effective degrees of freedom.

**Fig. 3:**
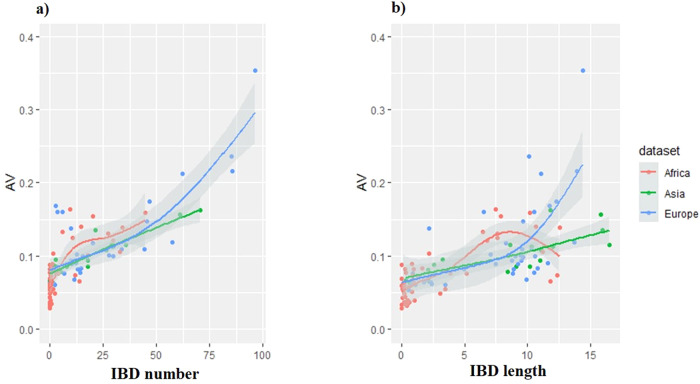
Plots of GAM models, with the variable to be predicted “AV index” and the predictors. **a** The mean “number of shared IBD segments” and (**b**) the mean “length of the shared IBD segments” (in Mb), taking into account the different goat datasets (African, Asian and European), see details in Table 4.

**Fig. 4:**
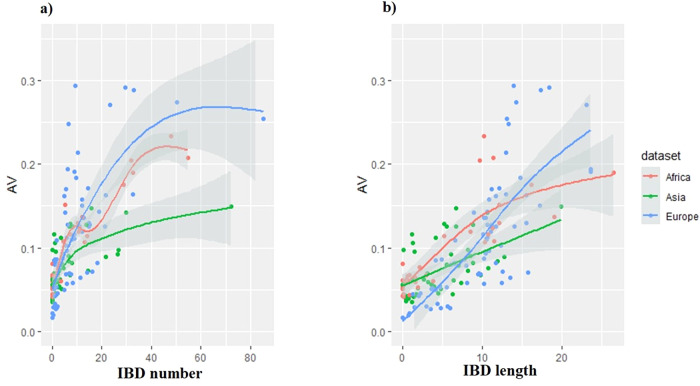
Plots of GAM models, with the variable to be predicted “AV index” and the predictors. **a** the mean “number of shared IBD segments” and (**b**) the mean “length of the shared IBD segments” (in Mb), taking into account the different sheep datasets (African, Asian and European), see details in Table 4.

Considering the “AV index” as the response (y) and shared IBD segments “number” and “length” as predictors (x) (Figs. 3, 4): robust models, indicating an overall increase in genetic originality as the “number” and “length” of IBD shared segments increased, were found. Strong adjusted R^2^ values were obtained, around 0.6, which even approached 0.8 in the goat dataset considering the “number” of IBD segment as the predictor (Table 4). It can be noted that the Asian dataset displayed linear or quasi-linear relationships.

**Table 4:** GAM models for goats and sheep considering the “AV index”.

Goat GAM Models for y=AV index	Edf	F-value (*p* value)	Adjusted R^2^
AV ~ IBD number	IBD number: 8.44	43.73 ( < 0.0001)	0.77
AV ~ IBD number*dataset origin	IBD number*Africa: 5.02 IBD number*Asia: 1.00 IBD number*Europe: 8.77	20.30 ( < 0.0001) 26.73 ( < 0.0001) 29.78 ( < 0.0001)	0.80
AV ~ IBD length	IBD length: 8.67	17.92 ( < 0.0001)	0.58
AV ~ IBD length*dataset origin	IBD length*Africa: 3.23 IBD length*Asia: 1.00 IBD length*Europe: 7.92	15.27 ( < 0.0001) 9.53 (0.002) 12.02 ( < 0.0001)	0.62
Sheep GAM Models for y=AV index			
AV ~ IBD number	IBD number: 6.63	30.00 ( < 0.0001)	0.59
AV ~ IBD number*dataset origin	IBD number*Africa: 1.83 IBD number*Asia: 3.02 IBD number*Europe: 8.69	30.25 ( < 0.0001) 7.17 ( < 0.0001) 23.17 ( < 0.0001)	0.66
AV ~ IBD length	IBD length: 3.31	47.38 ( < 0.0001)	0.56
AV ~ IBD length*dataset origin	IBD length*Africa: 3.22 IBD length*Asia: 1.00 IBD length*Europe: 3.99	10.26 ( < 0.0001) 16.54 ( < 0.0001) 39.16 ( < 0.0001)	0.59

With the variable to be predicted “AV index” and the predictors, the mean “number of shared IBD segments” or the mean “length of the shared IBD segments”. Edf=effective degrees of freedom.

### Sharing IBD patterns and crossbreeding: an example with Irish goat breeds

We exploited the opportunity to have genotypes of Old Irish goat breed (OIG) and also of crossbred individuals (OIGx). The Old Irish Goat is the native Irish breed, now critically endangered and found only in remote mountain ranges, living mostly in wild herds (www.oldirishgoat.ie). OIG are very hardy and adapted to their territory, even though they are supposed to have arrived in Ireland 5000 years ago in Neolithic times (Yalden 1999). In the latter half of the twentieth century, they were widely crossbred with improved-type goats (Swiss and Anglo-Nubian breeds), precipitating their decline (Porter and Tebbit 1996).

**Fig. 5:**
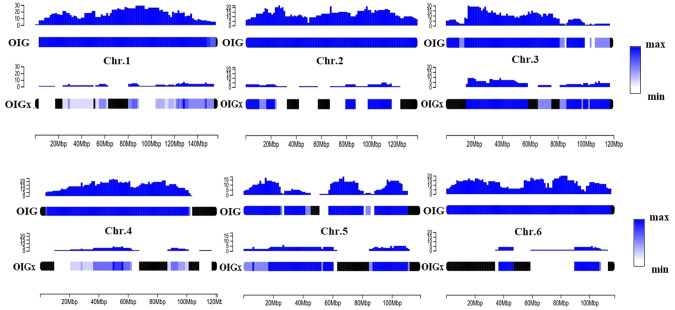
Shared IBD pattern representation, considering OIG and OIGx breeds, for chromosomes 1 to 6. Unshared portions of the genome are symbolized in black, while the blue gradient indicates the maximum number of shares towards deep blue, and a low number of shares towards white. The number of shared segments is represented by the histogram above the chromosome. On the x-axis, the scale indicates the position on the chromosome. Ten individuals in each breed have been considered.

For OIG, Admixture analysis revealed that 94% of the genome was shared by all individuals of the breed, compared to 45% for OIGx. Furthermore, OIG shared a very high number of IBD segments, 46.8 versus 9.8 for OIGx, and these shared segments were very long, averaging 12.3 Mb versus 2.1 Mb for OIGx. The Fig. 5 shows, by way of example, the number of shared segments for chromosomes 1 to 6, according to chromosomal position, and considering the same number of individuals for each breed (i.e. ten individuals). Unshared portions of the genome are symbolized in black, while the blue gradient indicates the maximum number of shares towards deep blue, and a low number of shares towards white. The number of shared segments is represented by the histogram above the chromosome. For OIGx, chromosome “mosaicism” was pronounced, with large proportions of genomes with little or no sharing, whereas for OIG, almost all parts of the chromosomes were shared, with frequency values at least 4 to 5 times higher than for crossbred individuals. Taking chromosome 6 as an example, 5 signatures associated with adaptation to temperature and altitude gradients, have been identified in Mediterranean goats by Serranito et al. (2021a), including signatures targeting: (i) the gene *GSTCD* (Glutathione S-Transferase C-Terminal Domain Containing), also identified by Wang et al. (2019) as being linked to hypoxia adaptation in yaks and by Mdladla (2016) as favoring adaptation to climate variables in South African goats. The gene (ii) *HERC6* (HECT and RLD Domain Containing E3 Ubiquitin Protein Ligase Family Member 6), was also found linked to climate adaptation in South African goats (Mdladla 2016), associated with stress grazing tolerance in sheep (Mwacharo et al. 2017), and associated with arid adaptation in Chinese sheep (Yang et al. 2016). In contrast to OIG, the *GSTCD* gene zone (chromosomal location 19,640,852–19,780,384 bp) was no longer shared by OIGx, and sharing of the *HERC6* gene was extremely low between OIGx individuals (chromosomal location 36,819,275–36,872,479 bp) (Fig. 5).

## Discussion

In this study, we assessed how the “length” and the “number” of IBD segments shared between individuals of the same breed could be informative of its admixture level, or, in other words, its isolation in terms of gene flow. To do this, we considered the available SNP genotype datasets for goats and sheep, and we retained datasets that allowed the most relevant screening of some countries or world regions.

We found that breeds classified as “admixed” showed fewer and smaller IBD segments in both goat and sheep datasets. Moreover, Spearman correlations were highly significant between the “genetic integrity level” and the “number” or “length” of IBD tracts, with values around 0.5, except for African goat breeds showing lower values and for African sheep breeds for which no correlation was shown. Spearman correlations between the “AV index” and the number or length of IBD segments, on the other hand, were all significant, with higher values around 0.7–0.8. In line with these results, statistical modeling showed particularly pronounced links between the “AV index” and the variables “number” or “length of shared IBD segments”. Different patterns appeared depending on the geographical origin of the samples. For example, GAM models showed more convoluted curves for African breeds, involving relationships between IBD patterns and levels of genetic originality, more complex to explain. Refining the analyses by considering more restricted and coherent geographical zones, for which a substantial number of genotyped breeds would be available, as well as precise information concerning management methods, appears essential in order to understand which parameters influence IBD-sharing patterns according to the regions of the world considered.

Taken together, the results showed a clear link between the genome fragmentation, inferred by the characteristics of the IBD segments, and the various indicators considered to assess the level of admixture of the different small ruminant breeds. It is interesting to note that the IBD pattern predicts the “AV index”, focusing on the strength of the population’s isolation rather than on the genetic similarity between individuals, more accurately than the “admixture level”, which does not integrate these notions of genetic proximity or originality.

All these results imply that the intra-breed shared segments of IBD patterns could be used to develop relevant indicators of admixture, or genetic isolation of breeds. More comprehensive theoretical and experimental studies, designed to take into account the evolutionary trajectories of the breeds (e.g. bottleneck experience, Marsden et al. 2016), and to precisely control the levels of admixture are needed to further our understanding of the phenomena. In particular, it is necessary to elucidate (i) which parameters induce a large “number” and/or “length” of IBD segments within breeds that are however admixed. Moreover (ii), it is important to disentangle the factors other than the admixture level (artificial selection pressure, inbreeding patterns, demographic histories, genetic drift, domestication, etc., see Xiang et al. 2022; Kristensen and Sørensen 2005; Orozco-TerWengel et al. 2015) that influence, differentially or not, the “length” and “number “of IBD shared segments. If we consider the “inbreeding” parameter, much work has been done on runs of homozygous genotypes (ROH) segments (i.e. long stretches of homozygous genotypes that commonly arise when individuals inherit haplotypes identical by descent) as indicators to estimate inbreeding levels and associated depression (Zhao et al. 2017; Peripolli et al. 2017). Indeed, the more recent the inbreeding, the longer these segments will tend to be, as few opportunities will have arisen for recombination to break them. For the “artificial selection pressure” parameter, methods also based on haplotype characteristics have been developed (Sabeti et al. 2002). The main property of positive selection is that it causes an unusually rapid increase in allele frequency, over a sufficiently short period to ensure that recombination does not lead to substantial breakdown of the haplotype on which the selected mutation occurs (Liu et al. 2013; Vanvanhossou et al. 2021). More generally, linkage disequilibrium in livestock has been largely influenced (Amaral et al. 2008), since domestication, by human decisions, which have governed livestock demography and the intensity and direction of artificial selection, itself closely linked to the level of inbreeding. Taking account of the different evolutionary trajectories is therefore essential in understanding haplotype patterns (Mészáros et al. 2021). Finally, (iii) the notion of timing needs to be further explored. The issue of crossbreeding in local breeds arose in the last few decades, so we are looking for an estimator that is particularly sensitive to recent events that have shaped genome profiles. Browning and Browning (2015) postulated that IBD segments could approximate the past 100 generations of demographic history. This rationale has been used to estimate the timing of recent mixing events (e.g., Patterson et al. 2004; Hoggart et al. 2004; Koopman et al. 2007).

To illustrate our point, we can apply our results to the conclusions of Belabdi et al. (2019), for North African sheep. The study revealed that, while the majority of Algerian and Moroccan breeds were highly admixed, the Sidaoun and Hamra breed (from pilot farms) were clearly differentiated (according to F_ST_ values, Weir and Cockerham 1984; ADMIXTURE; NetView, Steinig et al. 2016; fineSTRUCTURE, Lawson et al. 2012, analyses), appearing as not admixed. Analysis of IBD segments revealed that the ten highly admixed breeds shared an average number of intra-breed IBD segments close to zero, while for breeds appearing to be preserved from admixture, the mean number of segments was substantial at around 45.6 for Hamra but only at 0.11 for Sidaoun. The authors noted the discrepancy in the IBD sharing patterns, relative to other analyses for the Sidaoun breed. Combining these results with the present study, we can hypothesize that Sidaoun, would in fact probably be subject to outcrossing but with breeds not analyzed in the dataset. Indeed, the Sidaoun, bred by the Bedouins of the desert, is found in southern Algeria. For sanitary reasons, this breed cannot travel to the north, where all the other breeds analyzed by Belabdi et al., are found. It thus remains to study the links that Sidaoun breeders have with the southern border countries, particularly Mali and Niger, in order to identify whether the breed is indeed subject to crossbreeding via these as yet ungenotyped populations.

This study highlights the fact that European breeds (with the exceptions discussed below) appear to be globally better “preserved” than Asian and African breeds, probably due to early breed standardization as dictated by breed societies and stringent selection schemes. However, we must not neglect the fact that a breed may be in a good state of preservation in terms of admixture levels, but nevertheless suffer from genomic alterations induced by different factors, leading in particular to the erosion of genetic diversity. In fact, while selection schemes, with their strict control of sires, maintain the integrity of the breed, their application also leads to a reduction in the pool of genetic diversity, and taken to the extreme, intensive selection leads to severe loss of resilience (Doublet et al. 2020, Stachowicz et al. 2011, Taberlet et al. 2008, Rauw et al. 1998). For developing countries, the problem is quite different. If we consider the goat dataset, from East, North and West Africa, i.e. 17 countries represented through 55 breeds, we note that for 35 of them, less than one IBD segment shared, was recorded on average. These results probably reflect crossbreeding practices that weaken the resource by damaging its genetic integrity and the adaptive architecture established over time. In North Africa, Ouchene-Khelifi et al. (2018), showed a genetic homogenization of the goat stock, probably due to anarchic crossbreeding of breeders subjected to increasing economic pressure. In East Africa, Serranito et al. (2021b) highlighted very pronounced levels of admixture for goat breeds of Uganda, Tanzania and Kenya. The authors drew attention to the potentially decisive causal role of various structures involving religious organizations, government institutions, non-governmental organization, such as Heifer International, British Farm-Africa, National Livestock Production Development Programs, German-GTZ, etc. (Wilson et al. 1990; Mruttu et al. 2016). Indeed, these programs have been implemented since the 1980s to improve goats, essentially represented by small indigenous breeds highly adapted to the regions environment, such as the Small East African (SEA) goat, via crossbreeding with imported exotic breeds, notably Boer, Kamorai, Toggenburg, Saanen, Norwegian, Alpine and Anglo-Nubian.

The situation of the sheep resource in Spain spurred our attention. The ten breeds considered were all classified as “admixed” with an average number of IBD segments of 1.13. These values are very close to those found in Africa for goats, indicating highly fragmented genomes, potentially resulting from crossbreeding in the recent past and/or present. Careful analysis of the ADMIXTURE results (Supplementary Figure 2) reveals genetic proximity between certain Spanish breeds, that can be explained by a common origin (e.g. Latxa and Sasi Ardi belonging to the Churro branch, Rendo et al. 2004). However, for many breeds, this argument is hard to sustain. If we consider the Segurena breed, originating from the Entrefino branch, analyses showed that it shares significant proportions of its genome with breeds derived from the Iberico and Merino branches. This ancient breed, whose cradle is the Sierra de Segura, is an important part of the local heritage, with transhumance practices that have been known since the Middle Ages. Like a large number of local Spanish breeds, it is registered in the Official Catalog of Spanish Livestock, supervised by the Ministry of Agriculture (MAPA). However, it turns out that “industrial crossbreeding”, i.e. mating of males from meat breeds with females from local breeds, was a technique widely used in Spain between the 1970s and 1990s. In this respect, many crossbreeding studies have been carried out, notably with meat breeds known as Ovinos Precoces (Espejo et al. 1977; Esteban 2003). In particular, the Segurena breed has been tested for crosses with the Texel, Merino Landschaf and Ile de France breeds (Baro Shakery 1975). The interest in this issue remains valid today, as evidenced by work published in 2016, assessing the value of crosses between Texel and Segurena breeds (Blasco et al. 2016). As pointed out by Sierra Alfranca (1984), the use of these crossbreeds is not risk-free, since without strict F1 slaughtering, the spread of crossbred individuals in the populations can lead to the rapid breed’s loss. An in-depth study of the history of local Spanish breeds, with a particular focus on the agricultural policies pursued over the last few decades, seem essential to the understanding of the genetic results obtained in our study. Perea and Arias (2022) note that Spain is one of the countries with the greatest diversity of local breeds, thanks to an extensive and heterogeneous cultural and agro-ecological heritage. However, in the context of intensification of agriculture, there is a loss of economic competitiveness of traditional agricultural systems, and globally, an abandonment of local breeds. Our results suggest the urgent need to deepen genetic analyses and couple them with anthropological and historical studies in order to understand the changing dynamics in this country.

## Conclusion

This study highlighted the potential of IBD sharing patterns as indicators of admixture. Such indicators would optimize the protection of local breeds, enabling the detection of endangered breeds due to crossbreeding without the need for exhaustive knowledge of management practices and/or genotyping of breeds likely to be crossed with the breed in question, which is currently the case. The results also emphasize the fragmentation of genomes and the disruption of unique adaptation patterns, induced by crossbreeding practices driven by short-term productivity objectives.

### Supplementary information

Supplementary Material

## Data Availability

The goat AdaptMap dataset, is available via Dryad: 10.5061/dryad.v8g21pt). For sheep datasets : French breeds are available via Zenodo repository 10.5281/zenodo.237116; Italian and Spanish breeds via 10.23644/uu.8947346; Asian and North/Central European breeds via http://www.sheephapmap.org; Ethiopian breeds via www.animalgenome.org/repository/pub/KORE2017.1122 and also www.animalgenome.org/repository/pub/NOTT2018.0423; South African sheep breeds via osf.io/ceup6/?view_only=a1959659de5f4d5d9bbb1c607b2d83b6.
