# Supplementary material for: IBD sharing patterns as intra-breed admixture indicators in small ruminants
Source: Heredity (Edinb). 2023 Nov 3;132(1):30–42. doi: 10.1038/s41437-023-00658-x (PMC10799084; doi:10.1038/s41437-023-00658-x)
Supplement: Supplementary file 1 — Supplementary Material [file 41437_2023_658_MOESM1_ESM.pdf]

Supplementary Figures 1: ADMIXTURE analyses for goat breeds. For each dataset, (i) table showing membership proportion for each predefined breed in Qx clusters (x corresponding to the total number of breeds considered), highlighted in yellow proportion ≥ 0.1, breed code; (ii) Bayesian graph clustering. K = number of clusters. Finally, a PCA plot shows clusters of breeds for each dataset (see breed code in Supplementary Table 1).

	Q1	Q2	Q3	Q4	Q5	Q6	Q7	Q8	Q9	Q10	Q11	Q12	Q13	Q14	Q15	Q16	Q17	Q18	Q19	Q20	Q21	Q22	Q23	Q24	Q25	Q26	Q27	Q28	Q29	Q30	Q31	Q32	Q33
ABRmean	0.00	0.00	0.00	0.00	0.00	0.01	0.90	0.00	0.00	0.00	0.00	0.01	0.00	0.00	0.00	0.00	0.01	0.00	0.00	0.01	0.00	0.00	0.00	0.01	0.00	0.01	0.00	0.00	0.00	0.00	0.02	0.00	0.00
ANGmean	0.00	0.00	0.00	0.00	0.00	0.00	0.01	0.00	0.00	0.00	0.00	0.00	0.00	0.00	0.97	0.00	0.00	0.00	0.00	0.00	0.00	0.00	0.00	0.00	0.00	0.00	0.00	0.00	0.00	0.00	0.00	0.00	0.00
BAWmean	0.00	0.00	0.00	0.00	0.00	0.00	0.00	0.01	0.00	0.01	0.00	0.00	0.02	0.00	0.01	0.01	0.00	0.06	0.00	0.00	0.01	0.00	0.00	0.00	0.84	0.00	0.00	0.00	0.00	0.00	0.00	0.00	0.00
BOEmean	0.00	0.00	0.00	0.00	0.00	0.00	0.00	0.00	0.00	0.24	0.00	0.00	0.21	0.00	0.00	0.00	0.00	0.00	0.00	0.00	0.01	0.00	0.00	0.00	0.00	0.00	0.00	0.00	0.00	0.52	0.00	0.01	0.00
BURmean	0.00	0.01	0.00	0.00	0.00	0.00	0.00	0.01	0.00	0.00	0.00	0.00	0.00	0.00	0.00	0.00	0.00	0.01	0.00	0.00	0.00	0.00	0.91	0.00	0.01	0.00	0.01	0.00	0.00	0.00	0.00	0.00	0.01
DIAmean	0.01	0.00	0.42	0.00	0.00	0.00	0.00	0.00	0.00	0.00	0.43	0.00	0.00	0.00	0.00	0.19	0.00	0.00	0.00	0.00	0.00	0.00	0.00	0.00	0.00	0.00	0.00	0.00	0.00	0.00	0.00	0.00	0.00
DZDmean	0.00	0.00	0.00	0.00	0.00	0.00	0.00	0.00	0.00	0.00	0.00	0.00	0.00	0.00	0.00	0.00	0.00	0.00	0.00	0.00	0.00	0.00	0.01	0.00	0.96	0.00	0.00	0.00	0.00	0.01	0.00	0.00	0.00
GALmean	0.00	0.00	0.00	0.00	0.00	0.01	0.1	0.00	0.00	0.00	0.00	0.00	0.00	0.00	0.00	0.00	0.74	0.00	0.00	0.00	0.00	0.00	0.00	0.00	0.00	0.00	0.00	0.00	0.00	0.00	0.1	0.00	0.00
GOGmean	0.00	0.00	0.00	0.01	0.00	0.01	0.01	0.00	0.00	0.00	0.00	0.01	0.01	0.01	0.00	0.00	0.00	0.00	0.00	0.1	0.00	0.00	0.1	0.00	0.05	0.02	0.00	0.05	0.00	0.00	0.57	0.01	0.00
GUMmean	0.00	0.00	0.00	0.00	0.00	0.14	0.70	0.00	0.00	0.00	0.00	0.01	0.00	0.00	0.00	0.00	0.00	0.00	0.00	0.00	0.00	0.00	0.1	0.00	0.00	0.00	0.00	0.00	0.00	0.00	0.00	0.00	0.00
KARmean	0.00	0.00	0.00	0.00	0.00	0.03	0.02	0.00	0.00	0.00	0.00	0.01	0.00	0.01	0.00	0.00	0.43	0.00	0.00	0.01	0.00	0.01	0.17	0.01	0.01	0.02	0.00	0.00	0.00	0.00	0.24	0.00	0.00
KEFmean	0.00	0.00	0.00	0.00	0.00	0.83	0.05	0.00	0.00	0.00	0.00	0.01	0.00	0.00	0.00	0.00	0.05	0.00	0.00	0.01	0.00	0.00	0.02	0.01	0.00	0.00	0.00	0.00	0.00	0.00	0.02	0.00	0.00
LNDmean	0.00	0.00	0.00	0.01	0.00	0.00	0.00	0.01	0.00	0.01	0.00	0.00	0.01	0.00	0.01	0.00	0.00	0.81	0.01	0.00	0.03	0.00	0.00	0.00	0.06	0.00	0.00	0.00	0.00	0.00	0.00	0.02	0.01
MAAmean	0.00	0.00	0.00	0.00	0.00	0.01	0.00	0.00	0.00	0.00	0.00	0.00	0.00	0.01	0.00	0.00	0.1	0.00	0.00	0.11	0.00	0.00	0.01	0.04	0.02	0.02	0.00	0.01	0.00	0.00	0.65	0.00	0.00
MENmean	0.00	0.00	0.00	0.00	0.00	0.00	0.00	0.00	0.03	0.00	0.00	0.00	0.00	0.00	0.00	0.00	0.00	0.00	0.00	0.00	0.00	0.00	0.00	0.00	0.00	0.00	0.00	0.00	0.96	0.00	0.00	0.00	0.00
MLYmean	0.00	0.00	0.00	0.00	0.00	0.01	0.00	0.00	0.00	0.04	0.00	0.00	0.1	0.00	0.00	0.00	0.00	0.00	0.00	0.00	0.00	0.00	0.02	0.00	0.00	0.01	0.00	0.67	0.00	0.02	0.06	0.02	0.00
MSHmean	0.00	0.00	0.00	0.04	0.00	0.00	0.00	0.17	0.00	0.00	0.00	0.00	0.01	0.00	0.00	0.00	0.00	0.25	0.02	0.00	0.23	0.00	0.00	0.00	0.20	0.00	0.00	0.00	0.00	0.00	0.00	0.03	0.02
MTBmean	0.00	0.01	0.00	0.03	0.01	0.00	0.01	0.01	0.01	0.02	0.01	0.00	0.05	0.00	0.03	0.00	0.00	0.10	0.10	0.00	0.00	0.00	0.01	0.00	0.00	0.00	0.01	0.00	0.00	0.01	0.00	0.52	0.03
MTBxmean	0.00	0.00	0.00	0.28	0.00	0.00	0.00	0.00	0.00	0.00	0.00	0.00	0.00	0.00	0.00	0.00	0.00	0.02	0.38	0.00	0.01	0.00	0.00	0.00	0.00	0.00	0.00	0.00	0.00	0.00	0.00	0.01	0.28
MUBmean	0.00	0.00	0.00	0.00	0.00	0.01	0.01	0.00	0.00	0.01	0.00	0.01	0.01	0.28	0.00	0.00	0.02	0.00	0.00	0.01	0.00	0.00	0.50	0.00	0.00	0.01	0.00	0.00	0.00	0.00	0.12	0.00	0.00
NGDmean	0.00	0.00	0.00	0.00	0.00	0.02	0.01	0.00	0.00	0.02	0.00	0.00	0.03	0.06	0.01	0.00	0.02	0.00	0.00	0.01	0.00	0.00	0.62	0.01	0.00	0.01	0.00	0.01	0.00	0.02	0.13	0.01	0.00
NRWmean	0.00	0.02	0.00	0.00	0.00	0.00	0.00	0.00	0.00	0.00	0.00	0.00	0.00	0.00	0.00	0.00	0.00	0.00	0.00	0.00	0.00	0.01	0.00	0.00	0.01	0.00	0.89	0.00	0.00	0.00	0.03	0.00	0.00
PRWmean	0.00	0.00	0.00	0.00	0.00	0.01	0.00	0.00	0.00	0.00	0.00	0.01	0.00	0.00	0.00	0.00	0.03	0.00	0.00	0.67	0.00	0.00	0.01	0.01	0.00	0.01	0.00	0.00	0.00	0.00	0.24	0.00	0.00
SAAmean	0.00	0.23	0.00	0.00	0.63	0.00	0.00	0.00	0.00	0.00	0.00	0.00	0.01	0.00	0.00	0.00	0.00	0.00	0.00	0.01	0.00	0.00	0.00	0.00	0.00	0.00	0.00	0.01	0.00	0.00	0.05	0.01	0.00
SEAmean	0.00	0.00	0.00	0.00	0.00	0.01	0.01	0.01	0.00	0.01	0.00	0.01	0.02	0.01	0.00	0.00	0.23	0.14	0.00	0.02	0.00	0.00	0.22	0.01	0.03	0.04	0.00	0.01	0.00	0.01	0.19	0.01	0.00
SEAxmean	0.00	0.00	0.00	0.00	0.00	0.00	0.03	0.00	0.00	0.00	0.00	0.01	0.00	0.01	0.01	0.00	0.58	0.00	0.00	0.02	0.00	0.00	0.02	0.01	0.00	0.10	0.00	0.00	0.00	0.00	0.14	0.00	0.00
SEBmean	0.00	0.00	0.00	0.00	0.00	0.01	0.01	0.00	0.00	0.00	0.00	0.01	0.01	0.02	0.00	0.00	0.08	0.00	0.00	0.01	0.00	0.00	0.21	0.01	0.00	0.01	0.00	0.00	0.00	0.01	0.60	0.00	0.00
SNJmean	0.00	0.01	0.00	0.00	0.00	0.01	0.00	0.00	0.00	0.00	0.00	0.01	0.00	0.00	0.00	0.00	0.04	0.00	0.00	0.05	0.00	0.00	0.02	0.36	0.01	0.01	0.00	0.00	0.00	0.00	0.46	0.00	0.00
SOFmean	0.59	0.00	0.00	0.00	0.00	0.00	0.00	0.00	0.00	0.00	0.00	0.00	0.00	0.00	0.00	0.41	0.00	0.00	0.00	0.00	0.00	0.00	0.00	0.00	0.00	0.00	0.00	0.00	0.00	0.00	0.00	0.00	0.00
SOUmean	0.01	0.00	0.01	0.00	0.00	0.00	0.00	0.00	0.81	0.00	0.01	0.00	0.00	0.00	0.02	0.00	0.00	0.00	0.00	0.00	0.00	0.00	0.00	0.00	0.00	0.00	0.00	0.00	0.13	0.00	0.00	0.00	0.00
THYmean	0.00	0.00	0.00	0.00	0.00	0.00	0.00	0.02	0.00	0.00	0.00	0.00	0.00	0.00	0.00	0.00	0.00	0.19	0.00	0.00	0.01	0.00	0.00	0.00	0.77	0.00	0.00	0.00	0.00	0.00	0.00	0.00	0.00
TOGmean	0.00	0.02	0.00	0.00	0.01	0.00	0.00	0.00	0.00	0.00	0.00	0.00	0.00	0.00	0.00	0.00	0.01	0.00	0.00	0.01	0.00	0.49	0.00	0.00	0.00	0.00	0.02	0.01	0.00	0.00	0.09	0.00	0.30
WYGmean	0.00	0.00	0.00	0.00	0.00	0.11	0.02	0.00	0.00	0.00	0.00	0.22	0.00	0.00	0.00	0.00	0.51	0.00	0.00	0.01	0.00	0.00	0.02	0.01	0.00	0.01	0.00	0.00	0.00	0.00	0.05	0.00	0.00

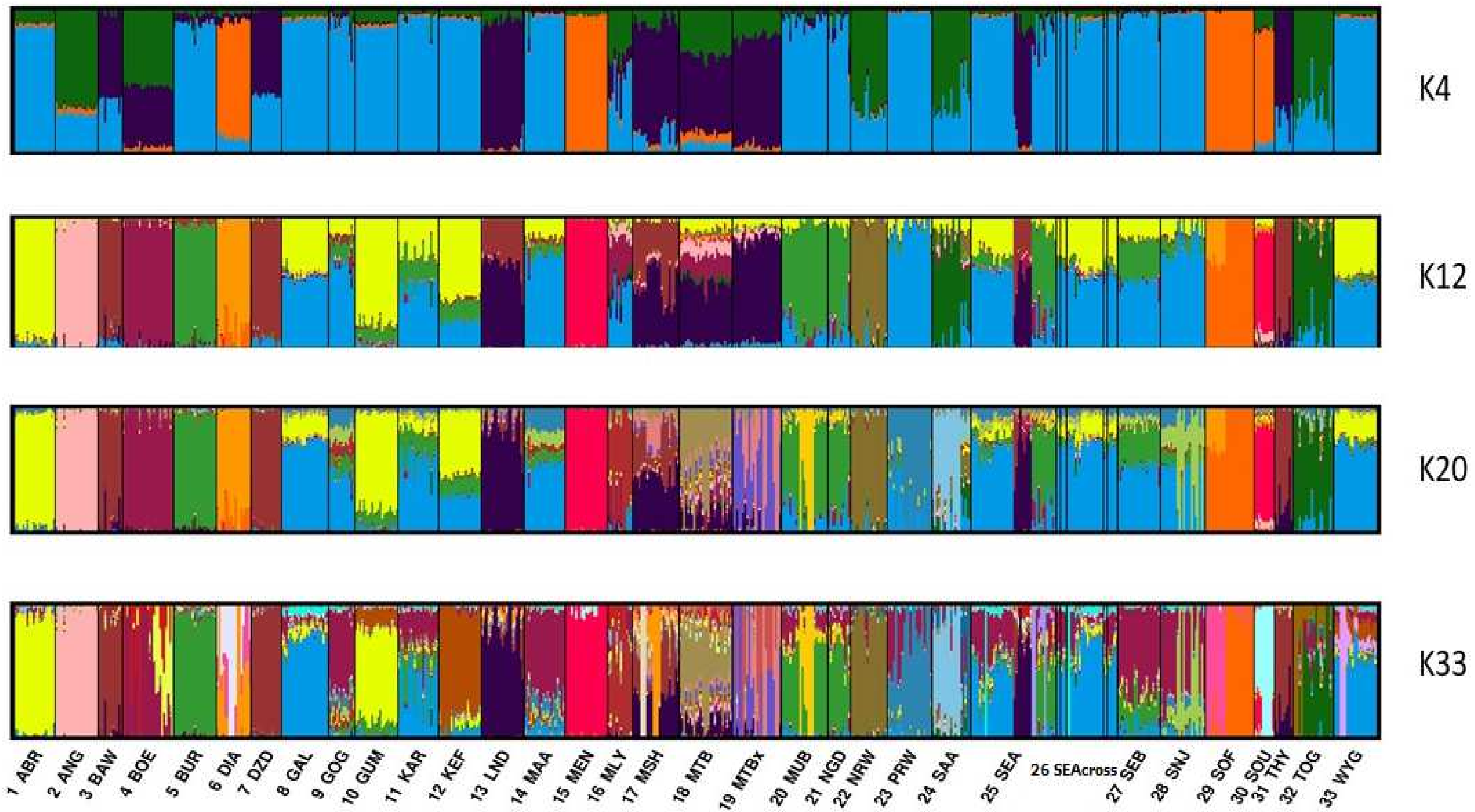

Goat dataset: East Africa

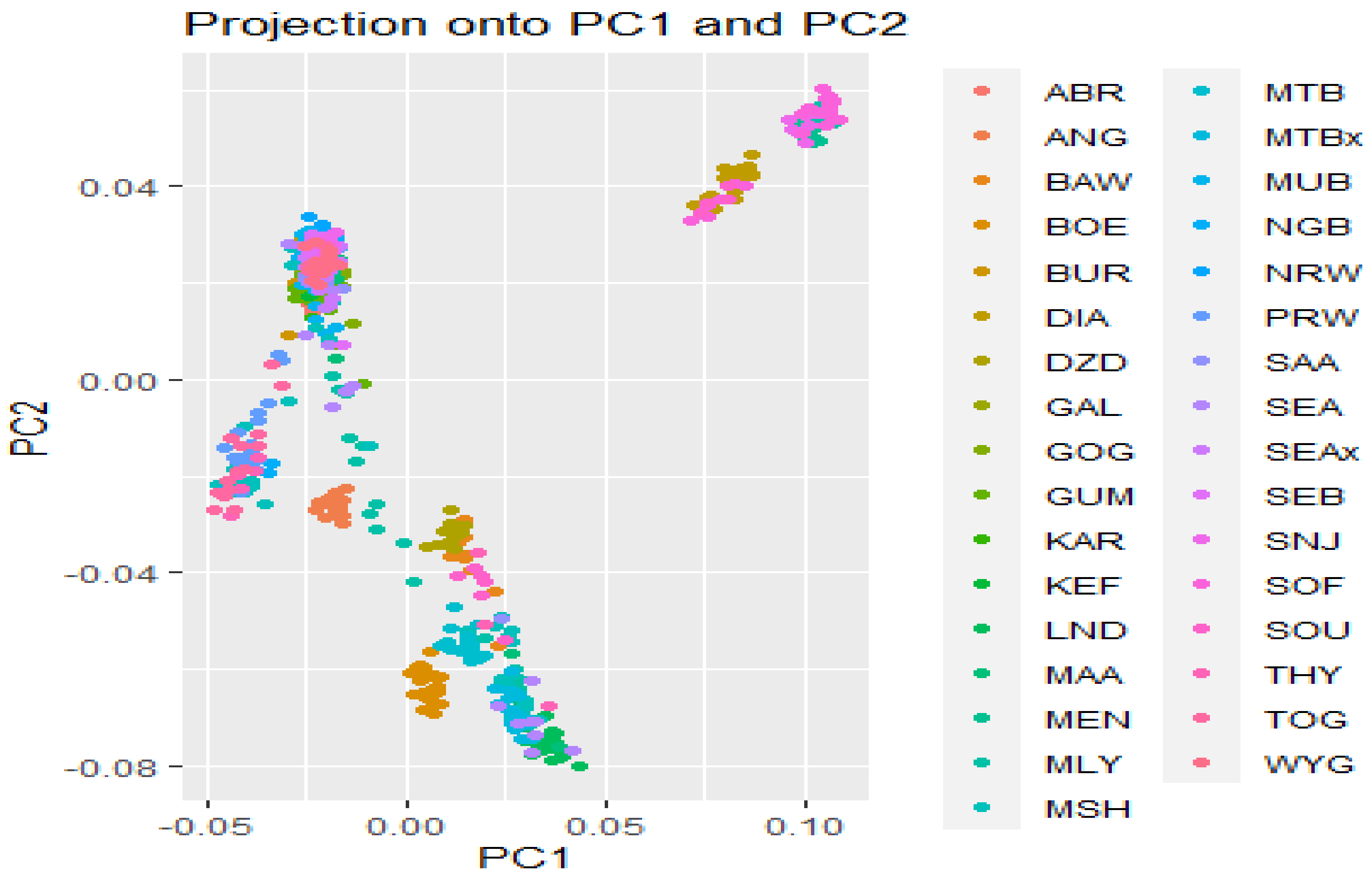

Goat dataset: East Africa

	Q1	Q2	Q3	Q4	Q5	Q6	Q7	Q8	Q9	Q10	Q11	Q12	Q13	Q14	Q15	Q16	Q17	Q18	Q19
BRKmean	0.00	0.00	0.03	0.40	0.01	0.00	0.01	0.01	0.00	0.01	0.00	0.00	0.32	0.04	0.01	0.02	0.01	0.00	0.13
CAMmean	0.01	0.00	0.00	0.00	0.67	0.01	0.04	0.00	0.00	0.19	0.01	0.00	0.01	0.00	0.00	0.03	0.00	0.00	0.00
DJAmean	0.00	0.01	0.01	0.00	0.00	0.81	0.01	0.01	0.00	0.00	0.06	0.00	0.00	0.00	0.02	0.06	0.00	0.03	0.00
GUEmean	0.00	0.00	0.03	0.00	0.01	0.02	0.00	0.01	0.42	0.00	0.00	0.16	0.01	0.00	0.00	0.1	0.23	0.01	0.01
MAUmean	0.00	0.01	0.63	0.01	0.02	0.02	0.00	0.02	0.01	0.01	0.00	0.01	0.01	0.00	0.03	0.20	0.01	0.01	0.01
MORmean	0.01	0.01	0.14	0.08	0.02	0.01	0.02	0.02	0.01	0.01	0.02	0.02	0.26	0.00	0.03	0.22	0.01	0.01	0.13
NAImean	0.00	0.00	0.00	0.00	0.00	0.39	0.00	0.00	0.00	0.00	0.02	0.00	0.00	0.00	0.05	0.04	0.01	0.47	0.00
NBNmean	0.00	0.00	0.00	0.01	0.00	0.00	0.00	0.00	0.00	0.00	0.00	0.00	0.12	0.86	0.00	0.00	0.00	0.00	0.01
OSSmean	0.00	0.00	0.00	0.03	0.00	0.00	0.00	0.00	0.00	0.00	0.00	0.00	0.26	0.04	0.00	0.00	0.00	0.00	0.65
PEUmean	0.01	0.01	0.06	0.00	0.01	0.05	0.00	0.36	0.00	0.02	0.00	0.01	0.00	0.00	0.03	0.41	0.00	0.01	0.00
RSKmean	0.00	0.01	0.03	0.00	0.31	0.02	0.02	0.02	0.01	0.00	0.22	0.00	0.01	0.00	0.02	0.30	0.00	0.00	0.01
SAHmean	0.02	0.02	0.05	0.00	0.03	0.06	0.00	0.06	0.01	0.01	0.02	0.00	0.00	0.00	0.03	0.64	0.01	0.01	0.01
SDNmean	0.01	0.01	0.04	0.00	0.01	0.25	0.00	0.02	0.00	0.01	0.03	0.01	0.00	0.00	0.29	0.24	0.01	0.06	0.01
SHLmean	0.00	0.01	0.04	0.00	0.37	0.01	0.01	0.02	0.00	0.00	0.08	0.00	0.08	0.00	0.02	0.32	0.00	0.01	0.02
SIDmean	0.00	0.00	0.01	0.05	0.06	0.00	0.00	0.00	0.00	0.00	0.00	0.00	0.60	0.03	0.01	0.00	0.01	0.00	0.22
TARmean	0.01	0.20	0.06	0.00	0.03	0.02	0.00	0.04	0.01	0.01	0.00	0.00	0.01	0.00	0.03	0.55	0.01	0.01	0.01
TUNmean	0.01	0.00	0.08	0.09	0.01	0.00	0.01	0.01	0.01	0.00	0.01	0.01	0.27	0.00	0.01	0.08	0.01	0.00	0.38
WADmean	0.00	0.00	0.00	0.00	0.11	0.00	0.84	0.00	0.00	0.01	0.01	0.00	0.01	0.00	0.00	0.00	0.00	0.00	0.00
WADnmean	0.12	0.00	0.00	0.00	0.03	0.00	0.00	0.00	0.00	0.00	0.81	0.00	0.00	0.00	0.00	0.02	0.00	0.00	0.00

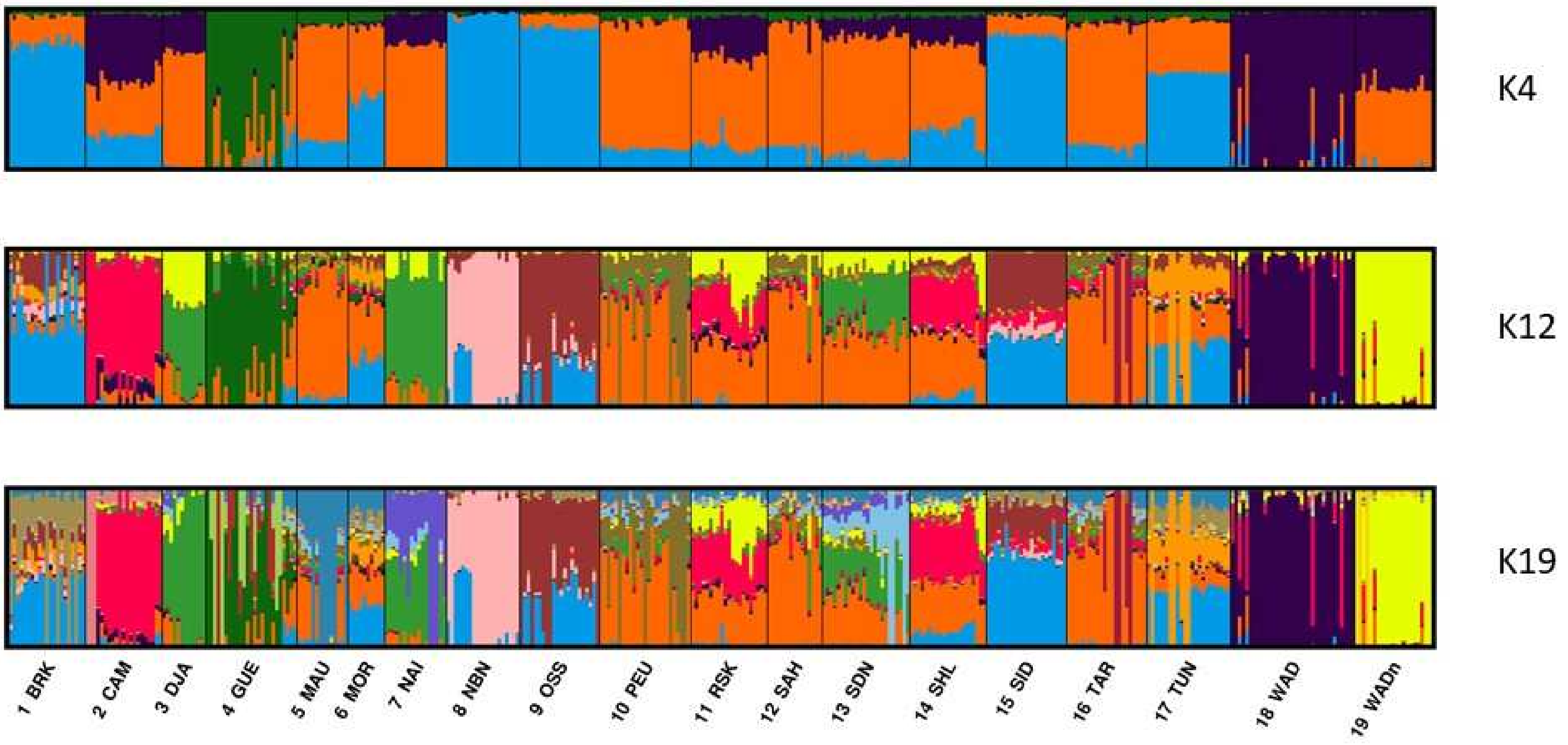

Goat dataset: North and West Africa

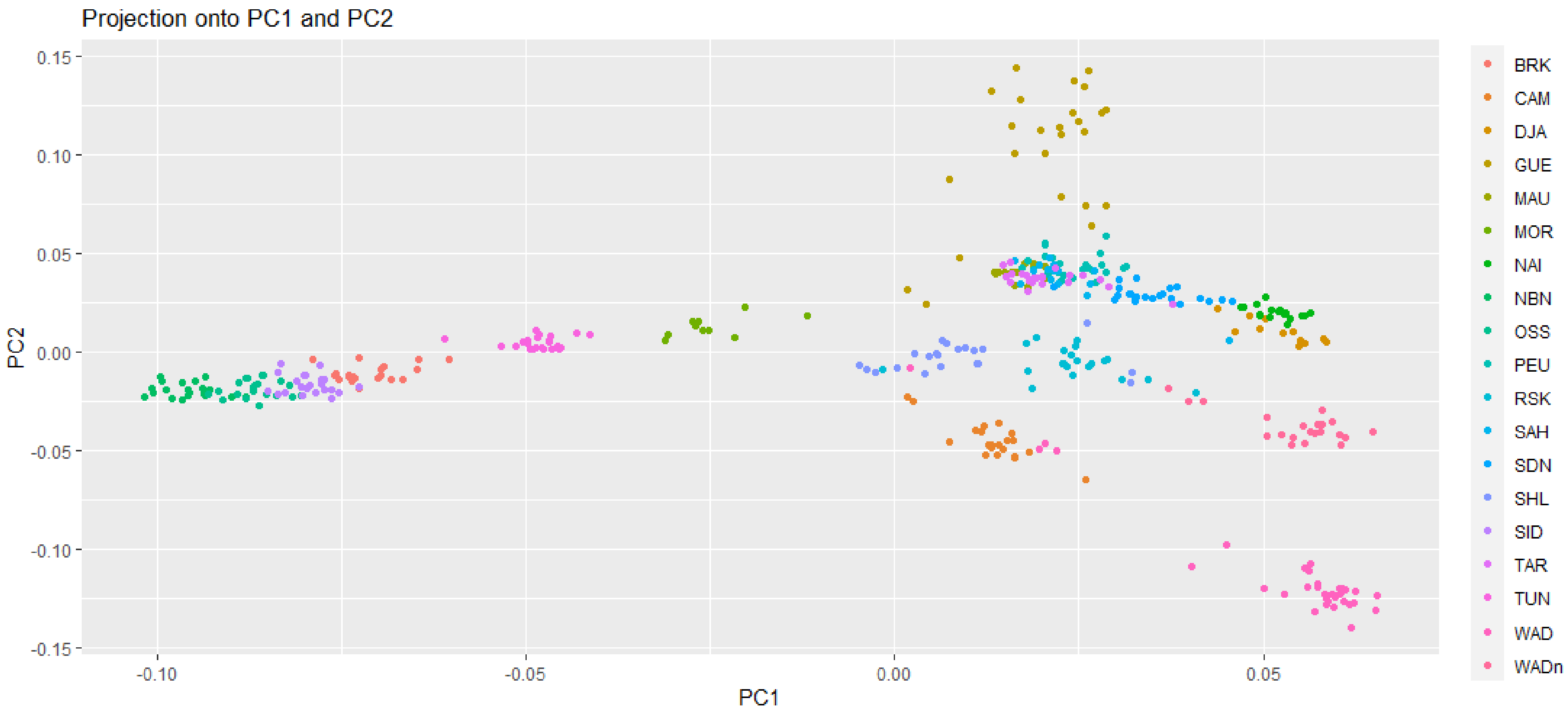

Goat dataset: North and West Africa

	Q1	Q2	Q3	Q4	Q5	Q6	Q7	Q8	Q9	Q10	Q11	Q12	Q13	Q14	Q15	Q16	Q17	Q18	Q19	Q20	Q21	Q22	Q23	Q24	Q25	Q26	Q27	Q28	Q29	Q30	Q31	Q32	Q33
ALPmean	0.00	0.00	0.00	0.01	0.00	0.00	0.00	0.00	0.01	0.00	0.00	0.00	0.01	0.00	0.00	0.00	0.00	0.01	0.00	0.90	0.00	0.00	0.01	0.00	0.01	0.00	0.00	0.00	0.00	0.00	0.00	0.00	0.01
ANGmean	0.00	0.00	0.00	0.00	0.00	0.00	0.01	0.00	0.00	0.00	0.00	0.00	0.00	0.00	0.62	0.00	0.00	0.00	0.00	0.00	0.00	0.00	0.00	0.00	0.00	0.00	0.00	0.00	0.00	0.00	0.35	0.00	0.00
ARGmean	0.38	0.08	0.00	0.00	0.01	0.01	0.00	0.01	0.00	0.04	0.03	0.00	0.00	0.02	0.01	0.01	0.07	0.02	0.03	0.01	0.00	0.00	0.00	0.01	0.01	0.02	0.01	0.05	0.01	0.08	0.00	0.02	0.05
ASPmean	0.37	0.26	0.00	0.01	0.00	0.01	0.00	0.00	0.00	0.03	0.02	0.00	0.01	0.02	0.01	0.00	0.04	0.01	0.01	0.01	0.00	0.00	0.01	0.00	0.00	0.01	0.01	0.02	0.04	0.05	0.01	0.01	0.02
BEYmean	0.00	0.00	0.81	0.01	0.00	0.00	0.00	0.02	0.00	0.00	0.00	0.00	0.01	0.00	0.00	0.01	0.01	0.00	0.00	0.01	0.00	0.01	0.00	0.01	0.00	0.00	0.01	0.00	0.00	0.00	0.00	0.05	0.00
BIOmean	0.01	0.01	0.01	0.01	0.05	0.01	0.00	0.00	0.02	0.00	0.00	0.00	0.02	0.00	0.00	0.01	0.01	0.65	0.00	0.03	0.00	0.00	0.02	0.00	0.01	0.01	0.00	0.01	0.00	0.00	0.00	0.01	0.10
BOEmean	0.00	0.00	0.00	0.00	0.00	0.00	0.52	0.00	0.00	0.00	0.00	0.44	0.00	0.00	0.00	0.00	0.00	0.00	0.00	0.00	0.01	0.00	0.00	0.00	0.00	0.00	0.00	0.00	0.00	0.00	0.01	0.00	0.00
CCGmean	0.39	0.02	0.01	0.01	0.01	0.04	0.00	0.00	0.01	0.00	0.01	0.00	0.01	0.00	0.00	0.00	0.09	0.02	0.04	0.01	0.00	0.00	0.02	0.01	0.00	0.02	0.00	0.01	0.01	0.02	0.01	0.00	0.20
CRSmean	0.01	0.01	0.00	0.01	0.01	0.01	0.00	0.00	0.00	0.00	0.00	0.00	0.01	0.00	0.00	0.00	0.69	0.02	0.01	0.01	0.00	0.00	0.01	0.00	0.01	0.01	0.00	0.01	0.00	0.00	0.00	0.00	0.13
DITmean	0.08	0.02	0.00	0.00	0.00	0.86	0.00	0.02	0.01	0.00	0.00	0.00	0.00	0.00	0.00	0.00	0.01	0.00	0.01	0.00	0.00	0.00	0.00	0.00	0.00	0.00	0.00	0.01	0.00	0.01	0.00	0.00	0.02
FSSmean	0.00	0.00	0.04	0.04	0.01	0.00	0.00	0.01	0.02	0.00	0.00	0.00	0.02	0.00	0.00	0.02	0.01	0.04	0.00	0.05	0.00	0.00	0.02	0.61	0.02	0.00	0.00	0.00	0.00	0.00	0.00	0.03	0.03
GARmean	0.04	0.00	0.00	0.00	0.00	0.00	0.01	0.00	0.00	0.01	0.02	0.01	0.00	0.00	0.01	0.00	0.01	0.00	0.68	0.00	0.01	0.00	0.01	0.00	0.00	0.01	0.00	0.00	0.00	0.11	0.02	0.00	0.02
GGTmean	0.02	0.67	0.00	0.00	0.00	0.00	0.00	0.00	0.00	0.00	0.00	0.00	0.00	0.00	0.00	0.00	0.00	0.00	0.00	0.00	0.00	0.00	0.00	0.00	0.00	0.00	0.00	0.00	0.28	0.00	0.00	0.00	0.00
JONmean	0.02	0.00	0.00	0.00	0.00	0.00	0.00	0.00	0.00	0.01	0.46	0.00	0.00	0.00	0.00	0.00	0.00	0.00	0.00	0.00	0.00	0.00	0.00	0.00	0.00	0.45	0.00	0.00	0.00	0.03	0.00	0.00	0.00
MALmean	0.00	0.00	0.05	0.00	0.00	0.00	0.00	0.02	0.00	0.00	0.00	0.00	0.00	0.00	0.00	0.01	0.01	0.00	0.00	0.00	0.01	0.60	0.00	0.00	0.00	0.00	0.03	0.00	0.00	0.00	0.00	0.25	0.00
MLGmean	0.0	0.00	0.1	0.00	0.00	0.00	0.00	0.02	0.00	0.02	0.01	0.00	0.00	0.01	0.00	0.00	0.00	0.00	0.00	0.01	0.02	0.02	0.00	0.00	0.01	0.01	0.05	0.00	0.00	0.01	0.01	0.65	0.01
MLSmean	0.05	0.01	0.00	0.00	0.00	0.00	0.00	0.00	0.00	0.14	0.02	0.00	0.00	0.63	0.00	0.00	0.04	0.00	0.00	0.00	0.01	0.00	0.00	0.00	0.00	0.00	0.00	0.01	0.01	0.02	0.01	0.04	0.00
MLTmean	0.08	0.01	0.00	0.00	0.00	0.00	0.00	0.00	0.00	0.66	0.03	0.00	0.00	0.07	0.01	0.00	0.00	0.00	0.00	0.00	0.01	0.00	0.01	0.00	0.00	0.02	0.00	0.01	0.01	0.04	0.00	0.03	0.00
MUGmean	0.00	0.00	0.06	0.00	0.00	0.00	0.00	0.02	0.00	0.00	0.00	0.00	0.00	0.00	0.00	0.01	0.00	0.00	0.00	0.01	0.01	0.01	0.00	0.00	0.00	0.00	0.70	0.00	0.00	0.00	0.00	0.13	0.00
NICmean	0.46	0.02	0.01	0.01	0.00	0.01	0.00	0.00	0.00	0.04	0.02	0.00	0.00	0.01	0.01	0.00	0.01	0.01	0.01	0.01	0.00	0.00	0.00	0.00	0.01	0.01	0.00	0.25	0.00	0.03	0.01	0.02	0.01
OROmean	0.00	0.00	0.00	0.00	0.91	0.00	0.00	0.00	0.00	0.00	0.00	0.00	0.01	0.00	0.00	0.00	0.00	0.04	0.00	0.01	0.00	0.00	0.00	0.00	0.00	0.00	0.00	0.00	0.00	0.00	0.00	0.00	0.01
PALmean	0.00	0.00	0.00	0.00	0.00	0.00	0.00	0.00	0.00	0.00	0.00	0.00	0.00	0.00	0.00	0.00	0.00	0.00	0.00	0.00	1.00	0.00	0.00	0.00	0.00	0.00	0.00	0.00	0.00	0.00	0.00	0.00	0.00
PTVmean	0.00	0.00	0.00	0.93	0.00	0.00	0.00	0.00	0.01	0.00	0.00	0.00	0.00	0.00	0.00	0.00	0.00	0.00	0.00	0.01	0.00	0.00	0.01	0.00	0.01	0.00	0.00	0.00	0.00	0.00	0.00	0.00	0.00
PVCmean	0.00	0.00	0.01	0.02	0.00	0.00	0.00	0.00	0.01	0.00	0.00	0.00	0.01	0.00	0.00	0.01	0.01	0.03	0.00	0.02	0.00	0.00	0.02	0.01	0.78	0.00	0.00	0.00	0.01	0.00	0.00	0.01	0.01
PYRmean	0.00	0.00	0.03	0.01	0.00	0.00	0.00	0.01	0.01	0.00	0.00	0.00	0.01	0.00	0.00	0.59	0.01	0.00	0.00	0.27	0.00	0.00	0.01	0.01	0.01	0.00	0.00	0.00	0.00	0.00	0.00	0.02	0.00
RASmean	0.00	0.00	0.11	0.01	0.00	0.00	0.00	0.63	0.00	0.00	0.00	0.00	0.00	0.00	0.00	0.01	0.00	0.00	0.00	0.01	0.00	0.01	0.00	0.00	0.00	0.00	0.02	0.00	0.00	0.00	0.00	0.18	0.00
RMEmean	0.05	0.01	0.00	0.00	0.00	0.00	0.00	0.00	0.00	0.02	0.02	0.00	0.00	0.01	0.00	0.00	0.00	0.00	0.01	0.00	0.00	0.00	0.00	0.00	0.00	0.02	0.00	0.01	0.00	0.81	0.00	0.01	0.00
SAAsmean	0.00	0.00	0.00	0.01	0.00	0.00	0.00	0.00	0.86	0.00	0.00	0.00	0.01	0.00	0.00	0.00	0.00	0.03	0.00	0.02	0.00	0.00	0.02	0.00	0.00	0.00	0.00	0.00	0.00	0.00	0.00	0.00	0.02
SAAmean	0.01	0.00	0.01	0.01	0.01	0.01	0.00	0.01	0.02	0.00	0.00	0.00	0.01	0.00	0.00	0.01	0.00	0.01	0.00	0.02	0.00	0.00	0.81	0.01	0.01	0.01	0.00	0.00	0.00	0.00	0.00	0.01	0.01
SAAimean	0.01	0.00	0.01	0.01	0.02	0.01	0.00	0.00	0.10	0.01	0.00	0.00	0.01	0.00	0.00	0.01	0.01	0.04	0.00	0.03	0.00	0.00	0.67	0.01	0.01	0.00	0.00	0.00	0.00	0.00	0.00	0.01	0.03
SARmean	0.03	0.01	0.01	0.00	0.00	0.00	0.00	0.00	0.00	0.07	0.01	0.00	0.00	0.05	0.00	0.00	0.50	0.00	0.00	0.00	0.03	0.01	0.00	0.00	0.00	0.00	0.01	0.00	0.01	0.03	0.00	0.17	0.00
VALmean	0.00	0.00	0.00	0.01	0.00	0.00	0.00	0.00	0.01	0.00	0.00	0.00	0.87	0.00	0.00	0.00	0.01	0.03	0.00	0.01	0.00	0.00	0.01	0.00	0.00	0.00	0.00	0.00	0.00	0.00	0.00	0.00	0.01
VSSmean	0.01	0.01	0.00	0.00	0.01	0.00	0.00	0.00	0.01	0.00	0.00	0.00	0.01	0.00	0.00	0.00	0.01	0.03	0.01	0.01	0.00	0.00	0.01	0.01	0.01	0.00	0.00	0.01	0.00	0.00	0.00	0.01	0.80

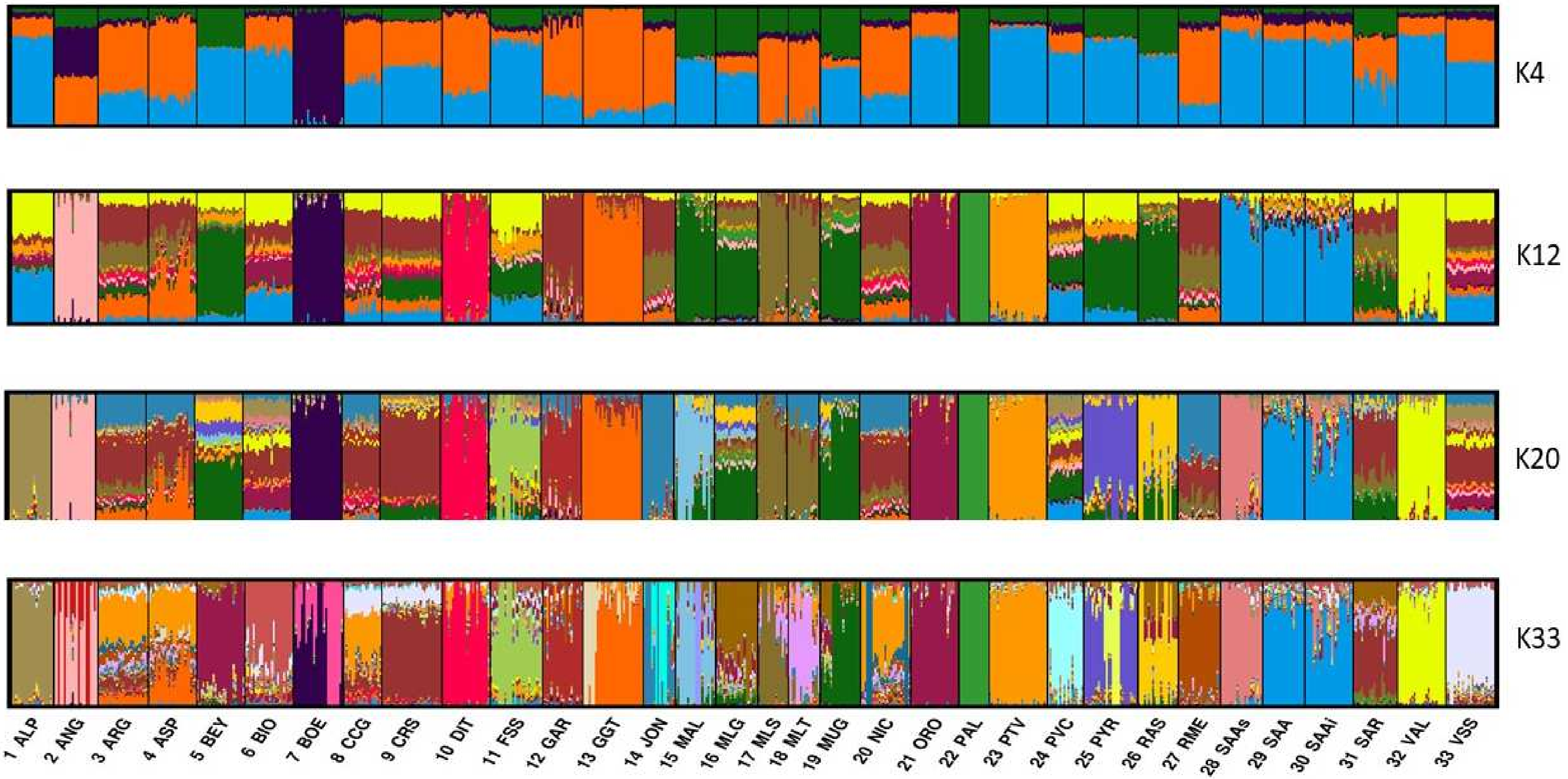

Goat datasets: France, Italy, Spain

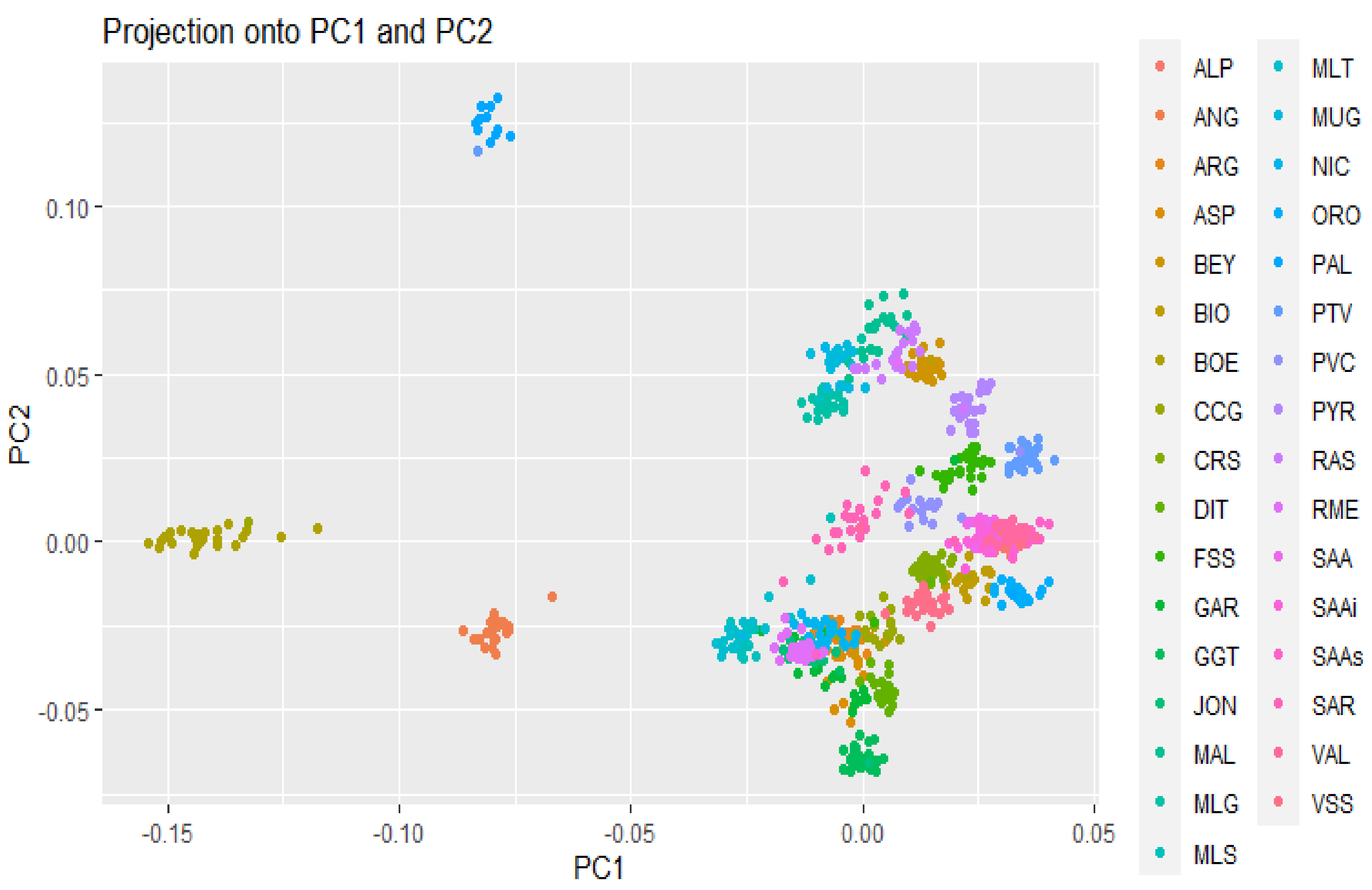

Goat datasets: France, Italy, Spain

	Q1	Q2	Q3	Q4	Q5	Q6	Q7	Q8
ARRmean	0.00	0.00	1.00	0.00	0.00	0.00	0.00	0.00
BLBmean	0.01	0.01	0.01	0.00	0.00	0.95	0.02	0.00
ICLmean	0.00	0.00	0.00	1.00	0.00	0.00	0.00	0.00
LNRDKmean	0.03	0.01	0.01	0.00	0.41	0.01	0.01	0.53
LNRFImean	0.98	0.00	0.00	0.00	0.00	0.00	0.00	0.01
LNRNLmean	0.00	0.99	0.00	0.00	0.00	0.00	0.00	0.00
OIGmean	0.01	0.01	0.01	0.00	0.00	0.01	0.94	0.01
OIGxmean	0.10	0.06	0.14	0.01	0.02	0.14	0.45	0.08

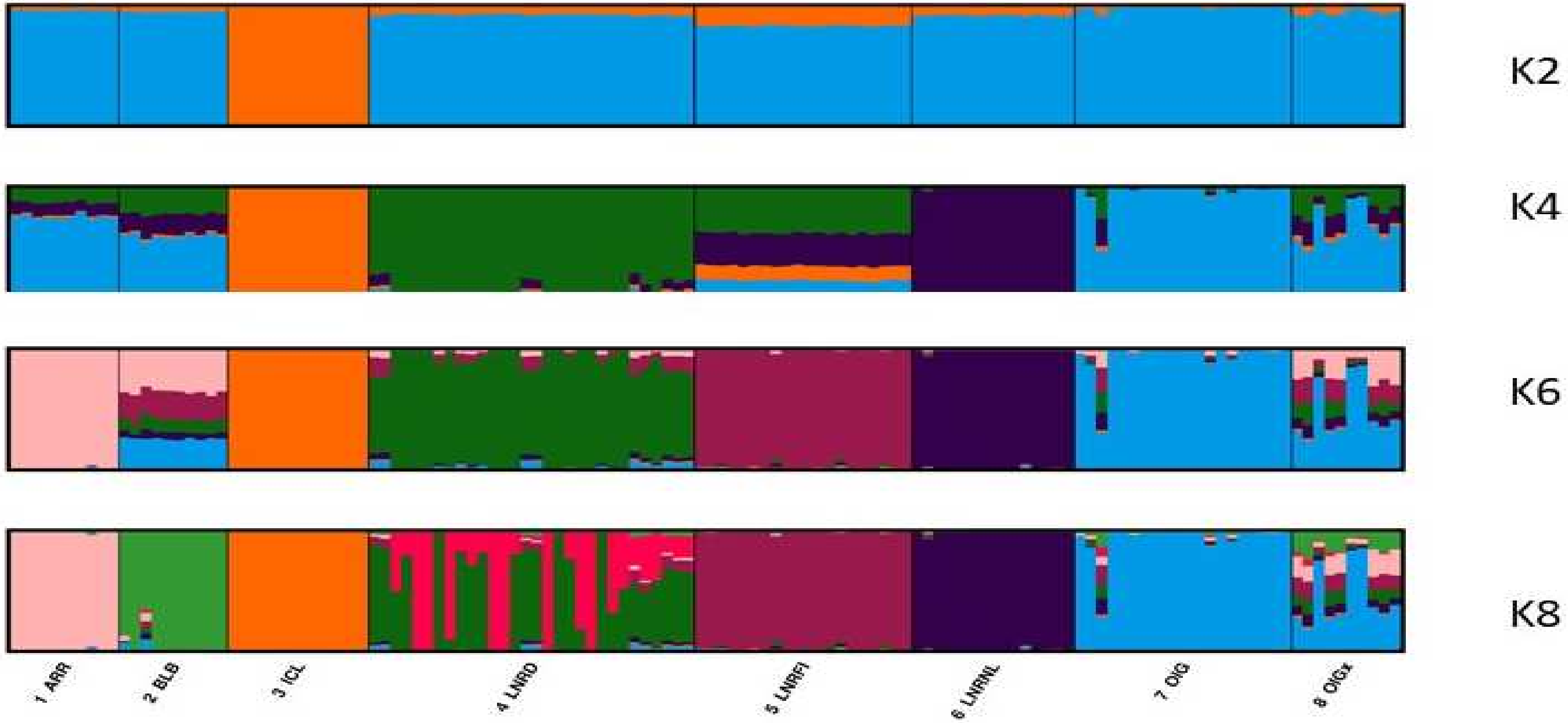

Goat dataset: North Europe

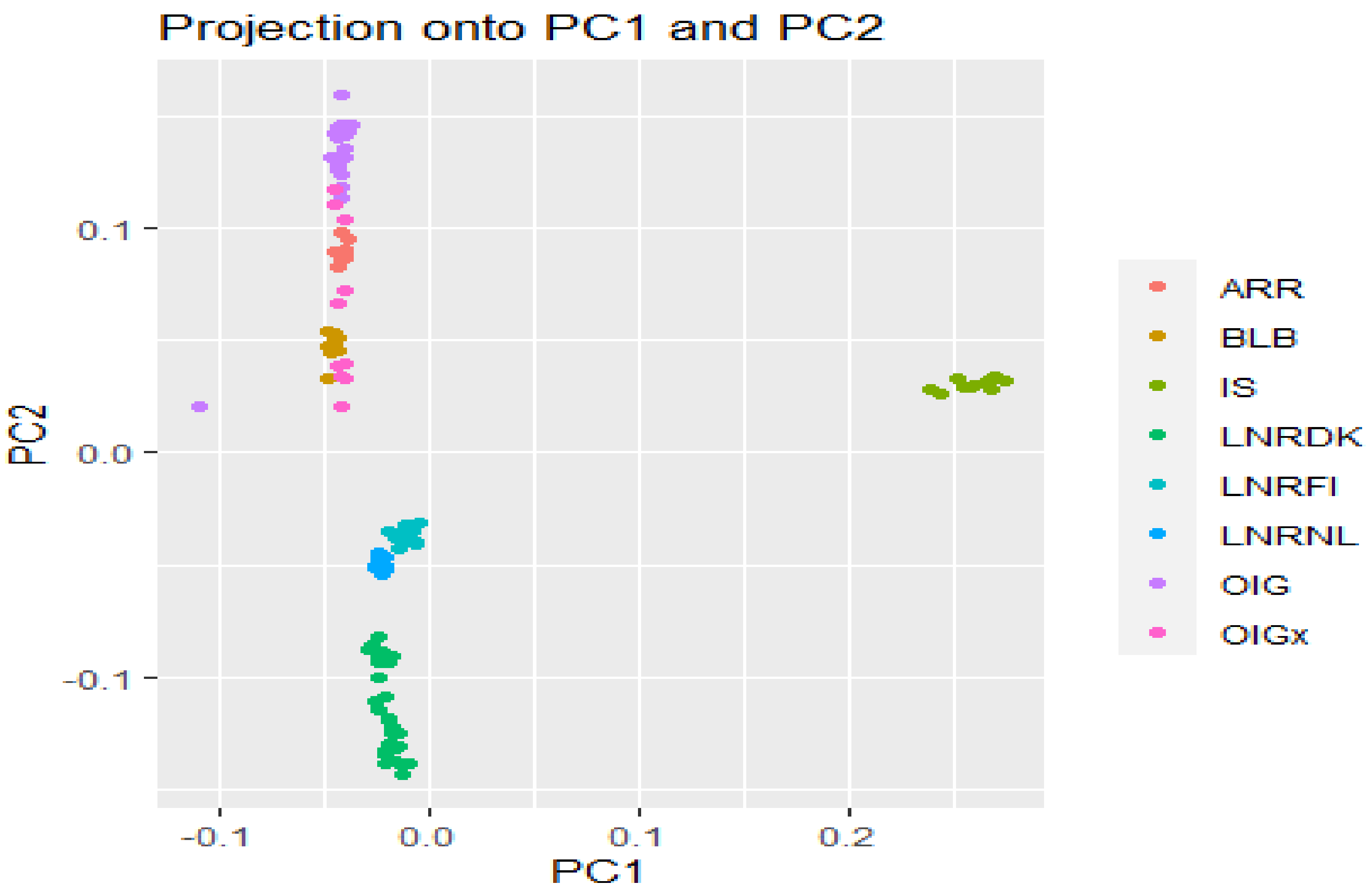

Goat dataset: North Europe

		Q1	Q2	Q3	Q4	Q5	Q6	Q7	Q8	Q9	Q10	Q11	Q12	Q13	Q14	Q15	Q16	Q17	Q18	Q19
ANKmean	adm	0.65	0.00	0.00	0.00	0.15	0.00	0.01	0.00	0.01	0.03	0.00	0.01	0.00	0.00	0.00	0.10	0.00	0.00	0.01
BABmean	pur	0.00	0.00	0.00	0.00	0.00	0.00	0.00	0.00	0.00	0.00	0.00	0.00	0.99	0.00	0.00	0.00	0.00	0.00	0.00
BRImean	adm	0.01	0.01	0.01	0.01	0.01	0.01	0.01	0.01	0.02	0.02	0.01	0.02	0.02	0.79	0.01	0.01	0.01	0.00	0.01
BUTmean	pur	0.00	0.00	0.01	0.36	0.00	0.02	0.48	0.01	0.01	0.01	0.01	0.00	0.00	0.00	0.01	0.00	0.02	0.00	0.07
CHAmean	adm	0.05	0.03	0.00	0.01	0.04	0.05	0.03	0.01	0.19	0.20	0.00	0.01	0.01	0.02	0.03	0.03	0.02	0.04	0.22
DDPmean	adm	0.00	0.70	0.00	0.00	0.00	0.01	0.00	0.00	0.11	0.07	0.00	0.00	0.01	0.00	0.01	0.00	0.01	0.01	0.06
JATmean	adm	0.00	0.01	0.00	0.01	0.00	0.02	0.02	0.01	0.03	0.05	0.01	0.01	0.01	0.00	0.05	0.00	0.61	0.01	0.15
KACmean	pur	0.00	0.00	0.00	0.00	0.00	0.00	0.01	0.00	0.01	0.01	0.00	0.00	0.00	0.00	0.92	0.00	0.03	0.00	0.02
KAMmean	adm	0.00	0.00	0.46	0.00	0.00	0.21	0.01	0.31	0.00	0.00	0.00	0.00	0.00	0.00	0.00	0.00	0.00	0.00	0.01
KESmean	adm	0.06	0.03	0.00	0.00	0.06	0.03	0.02	0.00	0.21	0.25	0.01	0.02	0.01	0.02	0.02	0.04	0.02	0.04	0.16
KILmean	adm	0.22	0.00	0.00	0.00	0.57	0.00	0.00	0.00	0.03	0.02	0.00	0.01	0.00	0.00	0.00	0.12	0.00	0.00	0.01
KLSmean	adm	0.17	0.00	0.00	0.00	0.17	0.00	0.00	0.00	0.02	0.03	0.00	0.01	0.01	0.01	0.00	0.54	0.00	0.00	0.01
LOHmean	adm	0.00	0.01	0.01	0.00	0.00	0.07	0.01	0.02	0.05	0.05	0.00	0.00	0.01	0.01	0.01	0.00	0.01	0.64	0.08
LOPmean	adm	0.00	0.01	0.00	0.00	0.00	0.00	0.01	0.02	0.03	0.84	0.00	0.01	0.00	0.00	0.01	0.00	0.00	0.01	0.04
PAHmean	adm	0.00	0.03	0.00	0.00	0.01	0.01	0.01	0.00	0.74	0.08	0.00	0.01	0.01	0.01	0.01	0.00	0.01	0.02	0.05
PATmean	adm	0.01	0.01	0.09	0.01	0.00	0.57	0.02	0.07	0.03	0.04	0.00	0.00	0.00	0.00	0.01	0.00	0.01	0.02	0.11
TAPmean	adm	0.00	0.00	0.01	0.00	0.00	0.01	0.01	0.00	0.01	0.01	0.33	0.00	0.00	0.01	0.00	0.00	0.01	0.00	0.59
TEDmean	adm	0.02	0.00	0.00	0.00	0.01	0.00	0.00	0.00	0.04	0.08	0.00	0.76	0.00	0.01	0.00	0.01	0.00	0.00	0.03
THAmean	adm	0.00	0.04	0.00	0.02	0.00	0.03	0.04	0.01	0.16	0.24	0.00	0.02	0.01	0.01	0.07	0.00	0.07	0.03	0.26

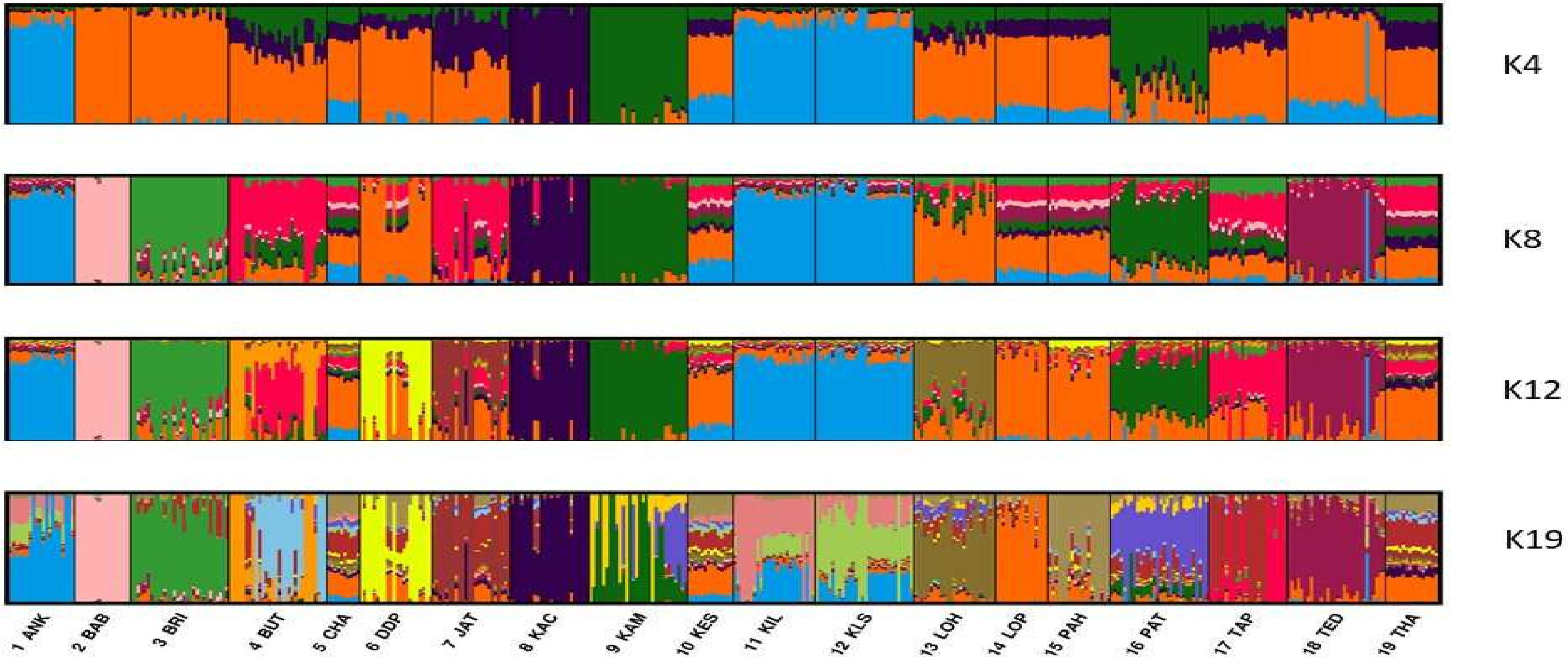

Goat dataset: Asia

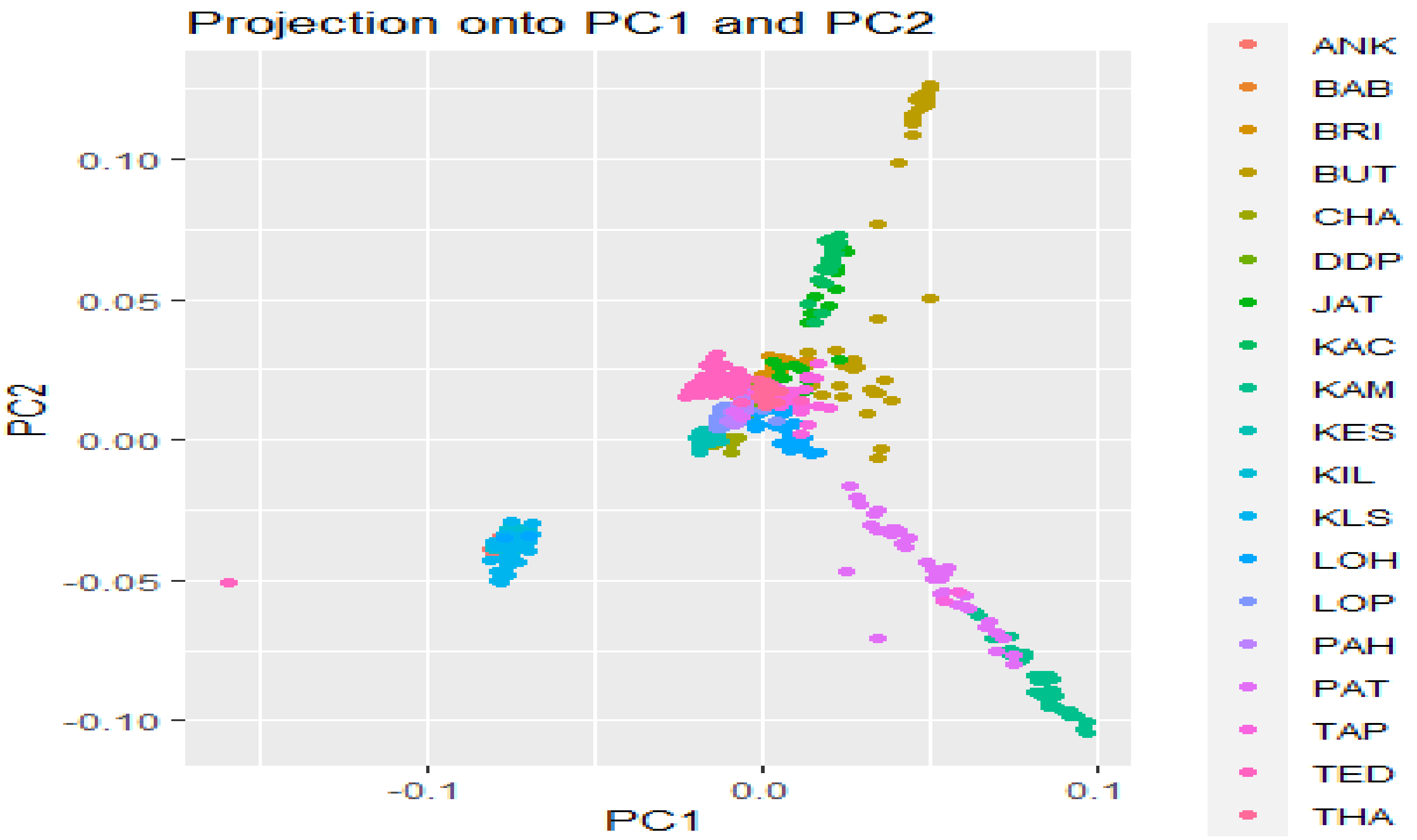

Goat dataset: Asia

Supplementary Figures 2: ADMIXTURE analyses for sheep breeds. For each dataset, (i) table showing membership proportion for each predefined breed in Qx clusters (x corresponding to the total number of breeds considered), highlighted in yellow proportion ≥ 0.1, breed code; (ii) Bayesian graph clustering. K = number of clusters. Finally, a PCA plot shows clusters of breeds for each dataset (see breed code in Supplementary Table 2).

	Q1	Q2	Q3	Q4	Q5	Q6	Q7	Q8
BGAmean	0.85	0.00	0.00	0.04	0.00	0.10	0.00	0.01
BGEmean	0.05	0.00	0.00	0.01	0.01	0.93	0.00	0.00
CHAmean	0.01	0.02	0.12	0.10	0.01	0.01	0.72	0.01
GARmean	0.02	0.00	0.00	0.04	0.90	0.03	0.00	0.00
GURmean	0.00	0.01	0.01	0.96	0.00	0.00	0.01	0.00
IDCmean	0.01	0.82	0.01	0.1	0.00	0.02	0.02	0.02
SUMmean	0.01	0.04	0.00	0.01	0.00	0.01	0.01	0.91
TIBmean	0.00	0.00	0.85	0.01	0.00	0.00	0.15	0.00

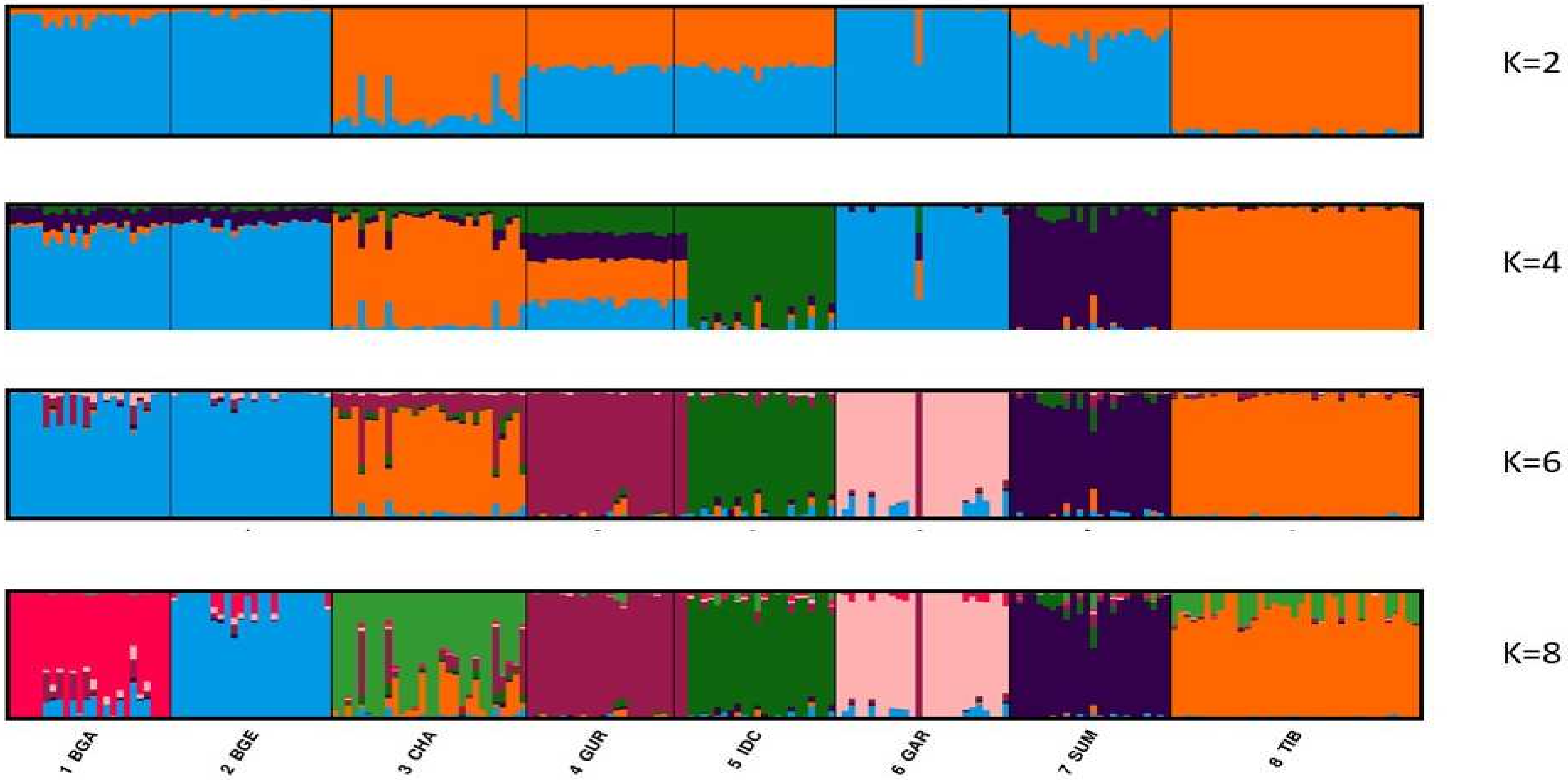

Sheep dataset : Pacific Asia

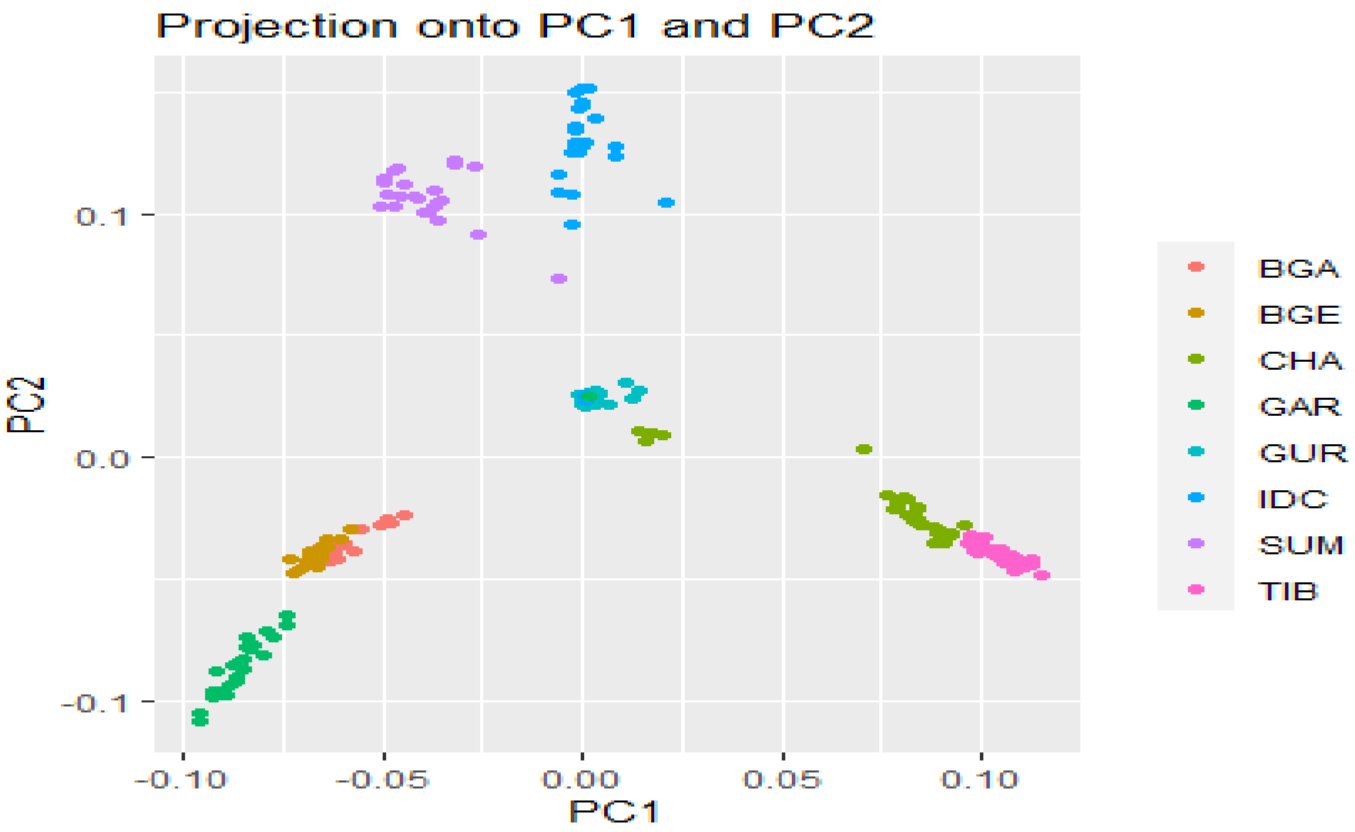

Sheep dataset : Pacific Asia

	Q1	Q2	Q3	Q4	Q5	Q6	Q7
AFSmean	0.02	0.79	0.01	0.02	0.01	0.00	0.14
CFTmean	0.01	0.01	0.01	0.00	0.66	0.28	0.04
KRSmean	0.13	0.02	0.01	0.63	0.01	0.01	0.19
MOGmean	0.04	0.05	0.01	0.04	0.02	0.01	0.84
NDZmean	0.85	0.01	0.00	0.06	0.00	0.00	0.07
QEZmean	0.10	0.14	0.02	0.11	0.03	0.01	0.59
SKZmean	0.00	0.00	0.96	0.00	0.00	0.00	0.03

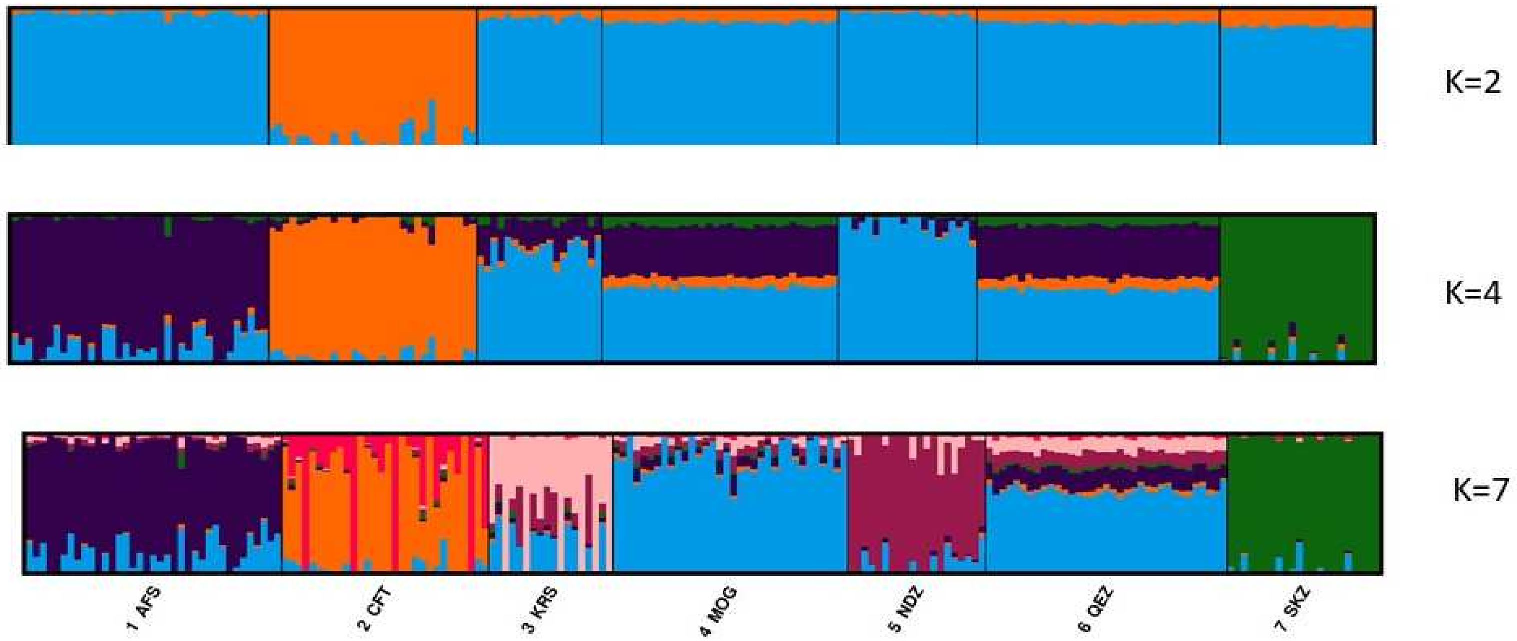

Sheep dataset : South West Asia

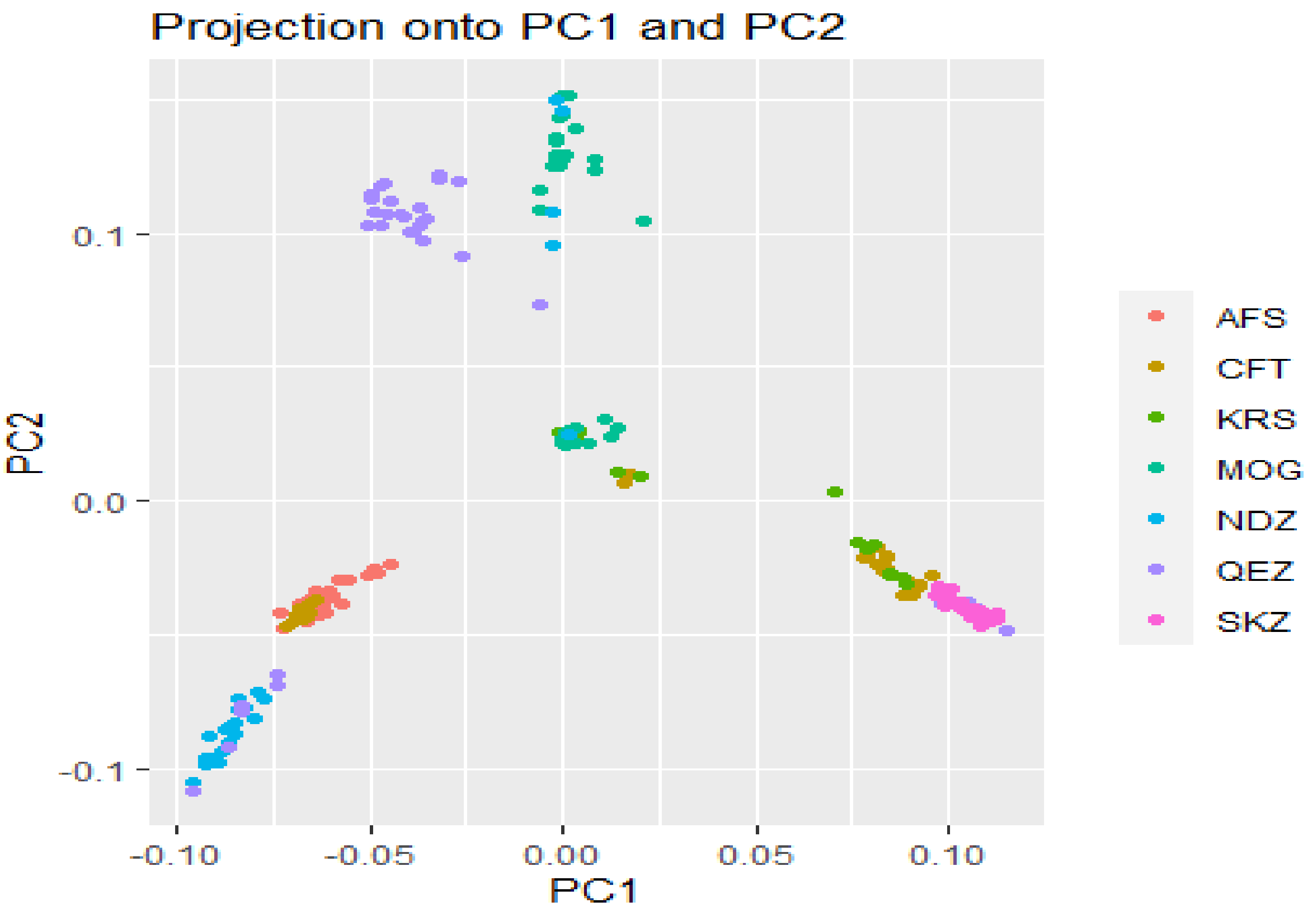

Sheep dataset : South West Asia

	Q1	Q2	Q3	Q4	Q5	Q6	Q7	Q8	Q9	Q10	Q11	Q12	Q13	Q14	Q15	Q16	Q17	Q18	Q19	Q20
BHMmean	0.04	0.05	0.00	0.01	0.00	0.01	0.04	0.04	0.00	0.01	0.01	0.00	0.01	0.01	0.03	0.01	0.59	0.09	0.01	0.03
BORmean	0.00	0.00	0.00	0.00	0.05	0.00	0.02	0.00	0.00	0.00	0.90	0.00	0.00	0.00	0.00	0.00	0.00	0.00	0.00	0.01
BOSmean	0.00	0.02	0.00	0.00	0.00	0.01	0.00	0.01	0.00	0.00	0.00	0.00	0.00	0.03	0.00	0.86	0.00	0.00	0.00	0.04
BRLmean	0.00	0.00	0.00	0.00	0.00	0.00	0.00	0.00	0.00	0.98	0.00	0.00	0.00	0.00	0.00	0.00	0.00	0.00	0.00	0.00
DSHmean	0.00	0.01	0.00	0.00	0.00	0.00	0.02	0.00	0.00	0.00	0.00	0.00	0.00	0.01	0.00	0.00	0.00	0.01	0.92	0.01
EFBmean	0.00	0.00	0.56	0.00	0.00	0.00	0.00	0.00	0.00	0.00	0.00	0.00	0.40	0.00	0.01	0.00	0.00	0.00	0.00	0.00
EFWmean	0.00	0.00	0.01	0.00	0.00	0.00	0.00	0.00	0.00	0.00	0.00	0.00	0.99	0.00	0.00	0.00	0.00	0.00	0.00	0.00
ERSmean	0.00	0.01	0.00	0.00	0.00	0.01	0.01	0.01	0.00	0.00	0.00	0.00	0.01	0.01	0.00	0.02	0.00	0.00	0.00	0.89
GALmean	0.92	0.00	0.00	0.00	0.00	0.00	0.00	0.01	0.01	0.01	0.00	0.00	0.00	0.00	0.03	0.00	0.00	0.00	0.00	0.00
GTXmean	0.02	0.01	0.00	0.01	0.00	0.01	0.01	0.02	0.05	0.02	0.00	0.00	0.02	0.00	0.76	0.01	0.01	0.03	0.00	0.01
ISFmean	0.01	0.00	0.00	0.00	0.00	0.00	0.00	0.00	0.00	0.00	0.00	0.19	0.00	0.00	0.00	0.00	0.00	0.78	0.00	0.00
SBFmean	0.00	0.01	0.00	0.01	0.00	0.01	0.91	0.00	0.00	0.00	0.01	0.00	0.00	0.00	0.00	0.01	0.00	0.01	0.01	0.01
SBSmean	0.01	0.81	0.00	0.00	0.00	0.01	0.01	0.01	0.00	0.01	0.00	0.00	0.00	0.02	0.01	0.01	0.01	0.01	0.00	0.04
SMSmean	0.01	0.01	0.00	0.00	0.00	0.80	0.00	0.11	0.00	0.00	0.00	0.00	0.00	0.01	0.00	0.01	0.00	0.00	0.00	0.03
SOAmean	0.00	0.00	0.00	0.00	1.00	0.00	0.00	0.00	0.00	0.00	0.00	0.00	0.00	0.00	0.00	0.00	0.00	0.00	0.00	0.00
STXmean	0.01	0.00	0.00	0.00	0.00	0.00	0.05	0.00	0.81	0.04	0.00	0.00	0.00	0.00	0.09	0.00	0.00	0.00	0.00	0.00
SWAmean	0.01	0.01	0.00	0.00	0.00	0.03	0.00	0.88	0.00	0.01	0.00	0.00	0.00	0.01	0.00	0.02	0.00	0.00	0.00	0.02
VBSmean	0.00	0.00	0.00	0.00	0.00	0.00	0.00	0.00	0.00	0.00	0.00	0.00	0.00	0.92	0.00	0.01	0.00	0.00	0.00	0.06
VRSmean	0.00	0.00	0.00	0.00	0.00	0.00	0.00	0.02	0.00	0.00	0.00	0.00	0.00	0.97	0.00	0.00	0.00	0.00	0.00	0.00
WILmean	0.00	0.00	0.00	0.97	0.00	0.00	0.00	0.00	0.00	0.01	0.00	0.00	0.00	0.00	0.00	0.00	0.00	0.00	0.00	0.00

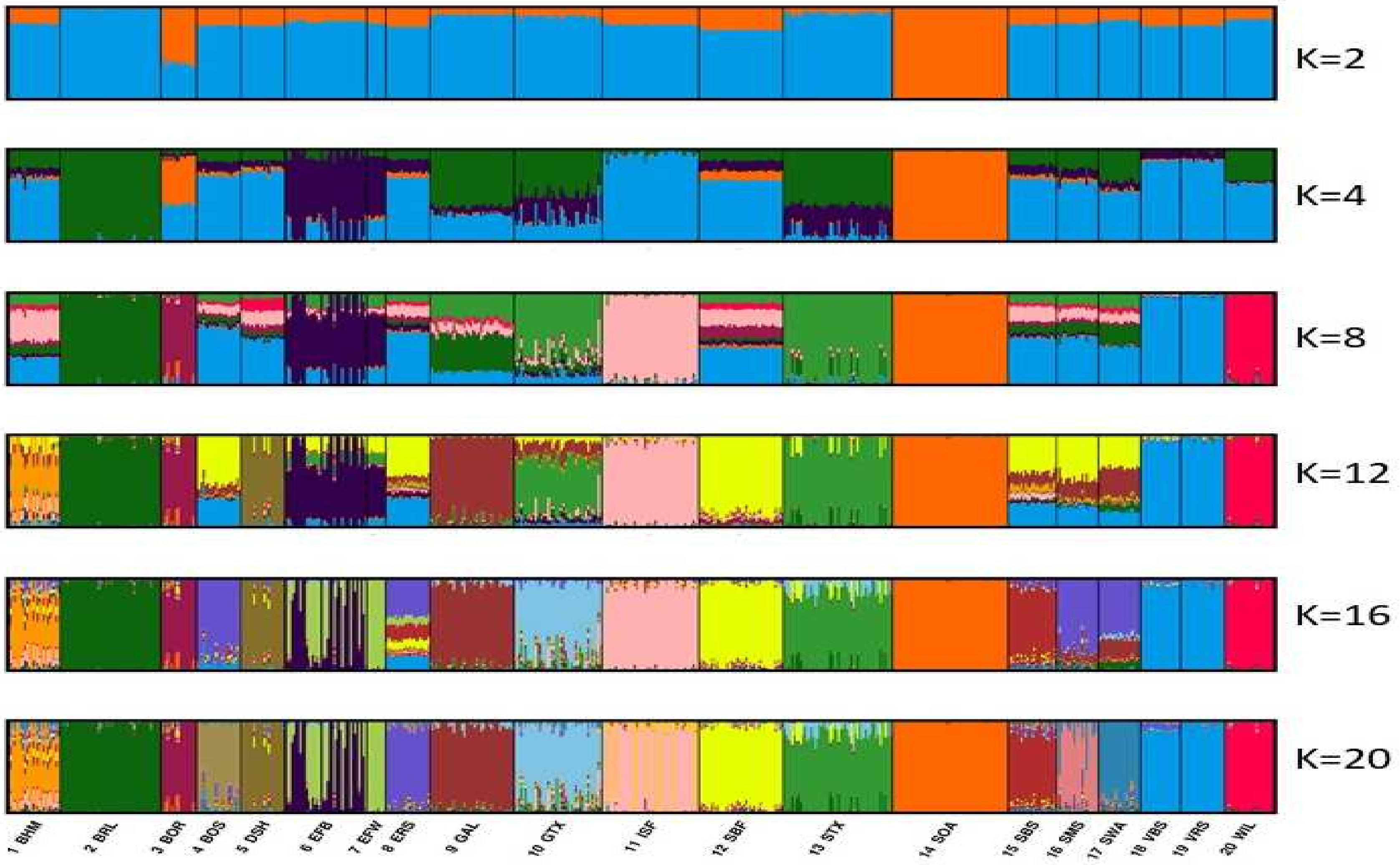

Sheep dataset : Central/North Europe

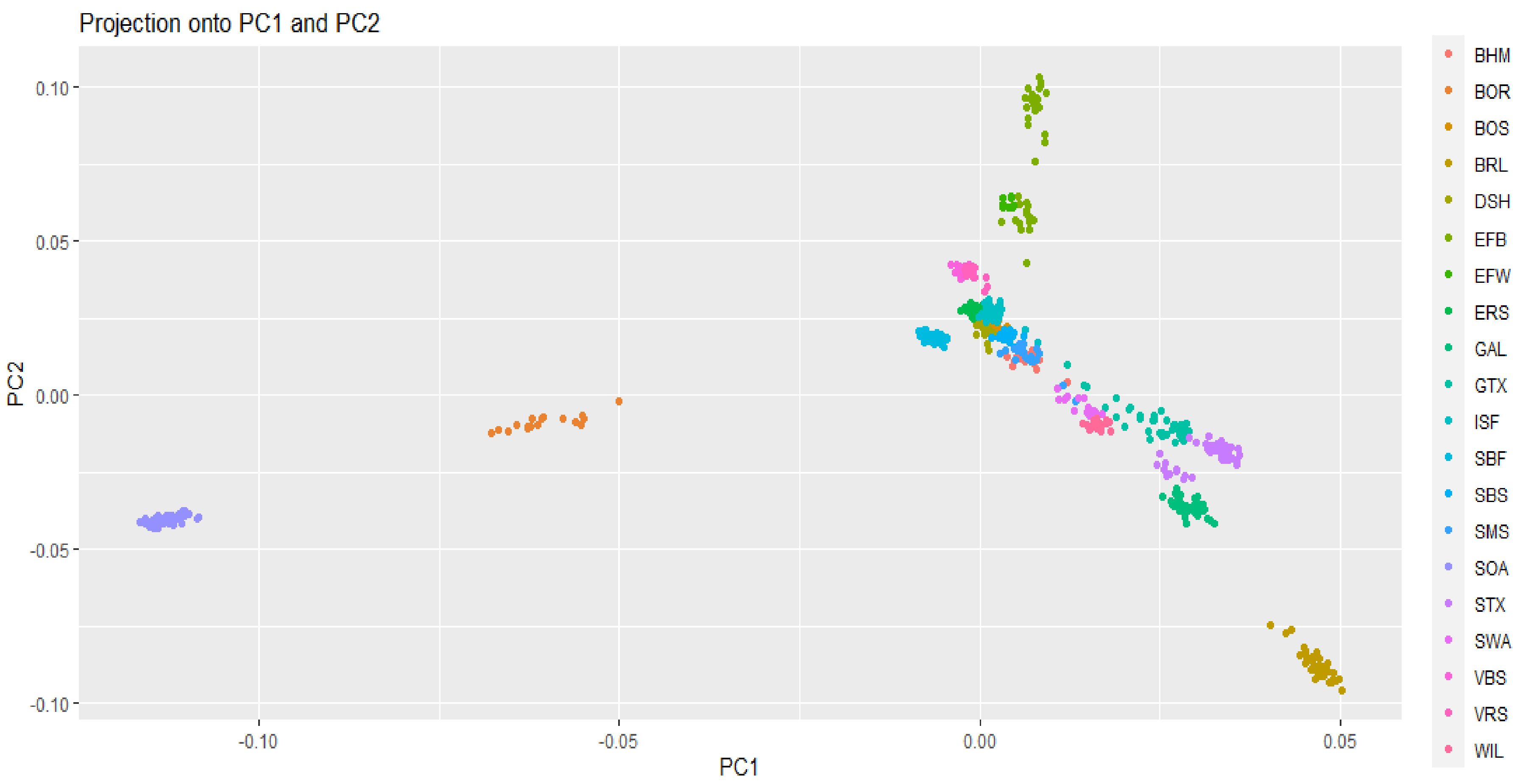

Sheep dataset : Central/North Europe

		Q1	Q2	Q3	Q4	Q5	Q6	Q7	Q8	Q9	Q10	Q11	Q12	Q13	Q14	Q15	Q16	Q17	Q18	Q19	Q20	Q21	Q22	Q23	Q24	Q25	Q26	Q27	Q28	Q29	Q30	Q31	Q32	Q33	Q34	Q35
BRK	adm	0,01	0,01	0,01	0,06	0,01	0,01	0,02	0,01	0,01	0,01	0,01	0,21	0,00	0,01	0,00	0,01	0,01	0,01	0,01	0,01	0,04	0,01	0,00	0,01	0,02	0,06	0,06	0,00	0,01	0,01	0,02	0,01	0,01	0,32	0,01
BSB	adm	0,01	0,14	0,01	0,01	0,06	0,02	0,01	0,01	0,01	0,01	0,02	0,30	0,00	0,01	0,11	0,01	0,02	0,01	0,01	0,02	0,03	0,01	0,01	0,01	0,02	0,01	0,01	0,01	0,01	0,01	0,02	0,01	0,01	0,01	0,02
CLS	adm	0,00	0,01	0,00	0,00	0,00	0,00	0,02	0,00	0,01	0,00	0,00	0,07	0,01	0,00	0,01	0,00	0,00	0,01	0,00	0,01	0,77	0,00	0,00	0,00	0,01	0,00	0,00	0,00	0,01	0,00	0,01	0,01	0,00	0,01	0,00
DQS	pur	0,00	0,00	0,00	0,00	0,00	0,00	0,00	0,00	0,00	0,00	0,00	0,01	0,00	0,00	0,00	0,00	0,00	0,04	0,00	0,01	0,00	0,00	0,00	0,00	0,00	0,00	0,00	0,58	0,00	0,32	0,00	0,00	0,00	0,00	0,00
GDS	adm	0,00	0,00	0,00	0,01	0,01	0,00	0,01	0,00	0,01	0,00	0,04	0,01	0,01	0,00	0,01	0,00	0,01	0,01	0,01	0,75	0,01	0,00	0,00	0,00	0,00	0,00	0,00	0,00	0,01	0,01	0,00	0,02	0,01	0,01	0,03
GLS	adm	0,00	0,01	0,01	0,17	0,01	0,01	0,00	0,00	0,01	0,01	0,01	0,09	0,01	0,00	0,00	0,00	0,00	0,01	0,00	0,01	0,01	0,00	0,00	0,00	0,02	0,00	0,52	0,00	0,00	0,00	0,03	0,01	0,00	0,00	0,01
HLS	adm	0,01	0,03	0,01	0,01	0,01	0,01	0,02	0,01	0,01	0,01	0,02	0,31	0,01	0,02	0,01	0,01	0,01	0,01	0,01	0,03	0,03	0,01	0,01	0,01	0,02	0,22	0,02	0,01	0,01	0,01	0,03	0,01	0,02	0,01	0,02
HTS	adm	0,00	0,02	0,00	0,00	0,01	0,01	0,45	0,00	0,02	0,01	0,01	0,18	0,01	0,00	0,01	0,01	0,01	0,00	0,01	0,03	0,09	0,01	0,01	0,00	0,03	0,00	0,00	0,00	0,01	0,00	0,01	0,02	0,01	0,02	0,01
HZS	pur	0,00	0,00	0,00	0,00	0,01	0,00	0,00	0,00	0,00	0,00	0,01	0,00	0,00	0,00	0,00	0,00	0,67	0,01	0,00	0,02	0,00	0,00	0,00	0,00	0,00	0,00	0,00	0,00	0,23	0,00	0,00	0,01	0,01	0,00	0,01
JZS	adm	0,01	0,04	0,03	0,03	0,01	0,02	0,02	0,01	0,01	0,02	0,01	0,29	0,01	0,01	0,02	0,01	0,01	0,02	0,01	0,03	0,03	0,00	0,01	0,01	0,06	0,01	0,05	0,01	0,01	0,01	0,10	0,02	0,02	0,01	0,03
KIR	adm	0,01	0,03	0,01	0,01	0,02	0,01	0,04	0,01	0,02	0,01	0,01	0,43	0,01	0,02	0,01	0,01	0,01	0,01	0,02	0,01	0,06	0,01	0,01	0,01	0,05	0,01	0,02	0,01	0,01	0,01	0,03	0,01	0,01	0,04	0,01
LAN	adm	0,01	0,01	0,00	0,01	0,01	0,00	0,05	0,01	0,00	0,01	0,01	0,28	0,01	0,01	0,00	0,01	0,00	0,01	0,01	0,03	0,07	0,01	0,01	0,01	0,30	0,00	0,01	0,00	0,01	0,00	0,01	0,02	0,01	0,04	0,01
LOP	adm	0,01	0,02	0,01	0,01	0,02	0,01	0,04	0,01	0,02	0,01	0,01	0,37	0,01	0,02	0,01	0,01	0,01	0,01	0,01	0,02	0,13	0,01	0,01	0,01	0,04	0,02	0,02	0,01	0,01	0,01	0,03	0,01	0,00	0,05	0,01
LZS	adm	0,00	0,02	0,23	0,02	0,00	0,01	0,01	0,00	0,01	0,01	0,01	0,11	0,00	0,02	0,01	0,01	0,00	0,00	0,00	0,00	0,01	0,00	0,00	0,00	0,36	0,01	0,03	0,00	0,01	0,01	0,05	0,00	0,01	0,01	0,01
MXS	adm	0,00	0,01	0,01	0,00	0,42	0,01	0,01	0,00	0,01	0,01	0,01	0,08	0,01	0,00	0,19	0,00	0,02	0,02	0,01	0,03	0,03	0,00	0,00	0,00	0,02	0,00	0,01	0,00	0,01	0,00	0,02	0,01	0,02	0,01	0,01
NLS	adm	0,02	0,00	0,01	0,00	0,00	0,00	0,00	0,02	0,05	0,00	0,00	0,02	0,00	0,15	0,00	0,00	0,00	0,59	0,01	0,00	0,00	0,03	0,01	0,02	0,01	0,00	0,00	0,01	0,00	0,02	0,00	0,00	0,00	0,00	0,00
SNS	adm	0,01	0,02	0,01	0,01	0,02	0,01	0,01	0,01	0,02	0,01	0,03	0,47	0,01	0,02	0,01	0,01	0,03	0,03	0,02	0,02	0,02	0,01	0,01	0,01	0,01	0,02	0,02	0,01	0,01	0,01	0,03	0,01	0,03	0,01	0,02
SPS	pur	0,00	0,00	0,00	0,00	0,00	0,00	0,00	0,00	0,55	0,00	0,00	0,00	0,00	0,00	0,00	0,00	0,00	0,00	0,45	0,00	0,00	0,00	0,00	0,00	0,00	0,00	0,00	0,00	0,00	0,00	0,00	0,00	0,00	0,00	0,00
TAN	adm	0,02	0,01	0,02	0,01	0,00	0,01	0,01	0,01	0,00	0,01	0,01	0,50	0,01	0,03	0,00	0,01	0,01	0,02	0,02	0,10	0,03	0,02	0,01	0,01	0,02	0,01	0,01	0,01	0,01	0,03	0,01	0,00	0,00	0,02	0,01
TCS	adm	0,00	0,00	0,00	0,00	0,00	0,00	0,00	0,01	0,03	0,00	0,00	0,01	0,00	0,81	0,00	0,00	0,00	0,07	0,01	0,00	0,00	0,01	0,00	0,00	0,00	0,00	0,00	0,01	0,00	0,00	0,00	0,00	0,00	0,00	0,00
THQ	adm	0,00	0,02	0,02	0,01	0,00	0,02	0,01	0,01	0,00	0,01	0,01	0,09	0,00	0,01	0,01	0,01	0,01	0,00	0,00	0,01	0,01	0,00	0,00	0,00	0,02	0,00	0,01	0,00	0,01	0,01	0,65	0,00	0,00	0,00	0,01
TLF	adm	0,01	0,02	0,01	0,08	0,01	0,01	0,01	0,01	0,01	0,01	0,01	0,21	0,01	0,01	0,01	0,01	0,09	0,01	0,01	0,11	0,02	0,00	0,00	0,01	0,03	0,01	0,08	0,01	0,12	0,00	0,03	0,01	0,01	0,02	0,01
TON	adm	0,00	0,68	0,01	0,01	0,01	0,01	0,01	0,00	0,01	0,01	0,01	0,08	0,00	0,00	0,00	0,01	0,01	0,01	0,00	0,01	0,01	0,00	0,00	0,00	0,01	0,00	0,01	0,00	0,01	0,00	0,02	0,01	0,01	0,01	0,01
TSK	adm	0,00	0,01	0,00	0,00	0,01	0,00	0,00	0,00	0,01	0,01	0,08	0,02	0,01	0,00	0,01	0,00	0,01	0,01	0,01	0,16	0,01	0,00	0,00	0,00	0,01	0,00	0,01	0,01	0,02	0,00	0,01	0,02	0,06	0,00	0,48
WGS	pur	0,00	0,00	0,00	0,00	0,00	0,00	0,00	0,01	0,05	0,00	0,00	0,00	0,00	0,00	0,00	0,00	0,00	0,89	0,01	0,00	0,00	0,01	0,00	0,01	0,00	0,00	0,00	0,00	0,00	0,00	0,00	0,00	0,00	0,00	0,00
WNS	pur	0,20	0,00	0,00	0,00	0,00	0,00	0,00	0,20	0,02	0,00	0,00	0,00	0,00	0,01	0,00	0,00	0,00	0,05	0,00	0,00	0,00	0,49	0,00	0,01	0,00	0,00	0,00	0,00	0,00	0,00	0,00	0,00	0,00	0,00	0,00
WRS	adm	0,01	0,02	0,02	0,02	0,01	0,02	0,02	0,01	0,02	0,01	0,03	0,42	0,01	0,01	0,01	0,01	0,02	0,02	0,01	0,03	0,02	0,01	0,01	0,02	0,03	0,02	0,02	0,01	0,01	0,01	0,04	0,02	0,03	0,01	0,02
WZS	adm	0,01	0,03	0,01	0,02	0,02	0,02	0,02	0,00	0,04	0,01	0,02	0,41	0,01	0,00	0,01	0,01	0,03	0,02	0,01	0,04	0,03	0,01	0,01	0,01	0,02	0,03	0,03	0,01	0,01	0,01	0,05	0,01	0,02	0,01	0,03
YEC	adm	0,01	0,02	0,01	0,01	0,01	0,01	0,05	0,01	0,01	0,01	0,01	0,27	0,01	0,01	0,01	0,01	0,01	0,01	0,01	0,06	0,07	0,00	0,01	0,01	0,08	0,01	0,18	0,00	0,01	0,01	0,02	0,02	0,01	0,02	0,01
YXZ	pur	0,00	0,01	0,00	0,00	0,00	0,29	0,00	0,00	0,00	0,41	0,00	0,01	0,00	0,00	0,00	0,22	0,00	0,00	0,00	0,00	0,00	0,00	0,00	0,00	0,00	0,00	0,00	0,00	0,01	0,00	0,01	0,00	0,00	0,00	0,00
ZCD	adm	0,00	0,01	0,00	0,01	0,01	0,00	0,00	0,00	0,01	0,00	0,58	0,00	0,01	0,00	0,01	0,00	0,02	0,01	0,00	0,15	0,01	0,00	0,00	0,00	0,00	0,00	0,00	0,01	0,01	0,00	0,00	0,02	0,05	0,00	0,05
ZLZ	pur	0,00	0,00	0,00	0,00	0,01	0,00	0,00	0,00	0,01	0,00	0,03	0,02	0,71	0,01	0,01	0,00	0,01	0,01	0,00	0,06	0,01	0,00	0,01	0,00	0,00	0,00	0,00	0,00	0,01	0,01	0,00	0,03	0,02	0,00	0,02
ZNQ	adm	0,00	0,01	0,00	0,00	0,01	0,00	0,01	0,00	0,01	0,00	0,07	0,01	0,01	0,00	0,01	0,00	0,02	0,02	0,00	0,14	0,00	0,00	0,01	0,00	0,00	0,00	0,00	0,01	0,01	0,00	0,00	0,04	0,53	0,01	0,05
ZRK	adm	0,00	0,00	0,00	0,00	0,00	0,00	0,00	0,00	0,00	0,00	0,02	0,01	0,00	0,00	0,00	0,00	0,01	0,01	0,00	0,04	0,00	0,00	0,00	0,00	0,00	0,00	0,00	0,00	0,01	0,00	0,00	0,81	0,02	0,00	0,02
ZTS	adm	0,00	0,00	0,00	0,00	0,00	0,00	0,00	0,00	0,01	0,00	0,00	0,00	0,00	0,00	0,00	0,00	0,00	0,01	0,00	0,00	0,00	0,00	0,24	0,73	0,00	0,00	0,00	0,00	0,00	0,00	0,00	0,00	0,00	0,00	0,00

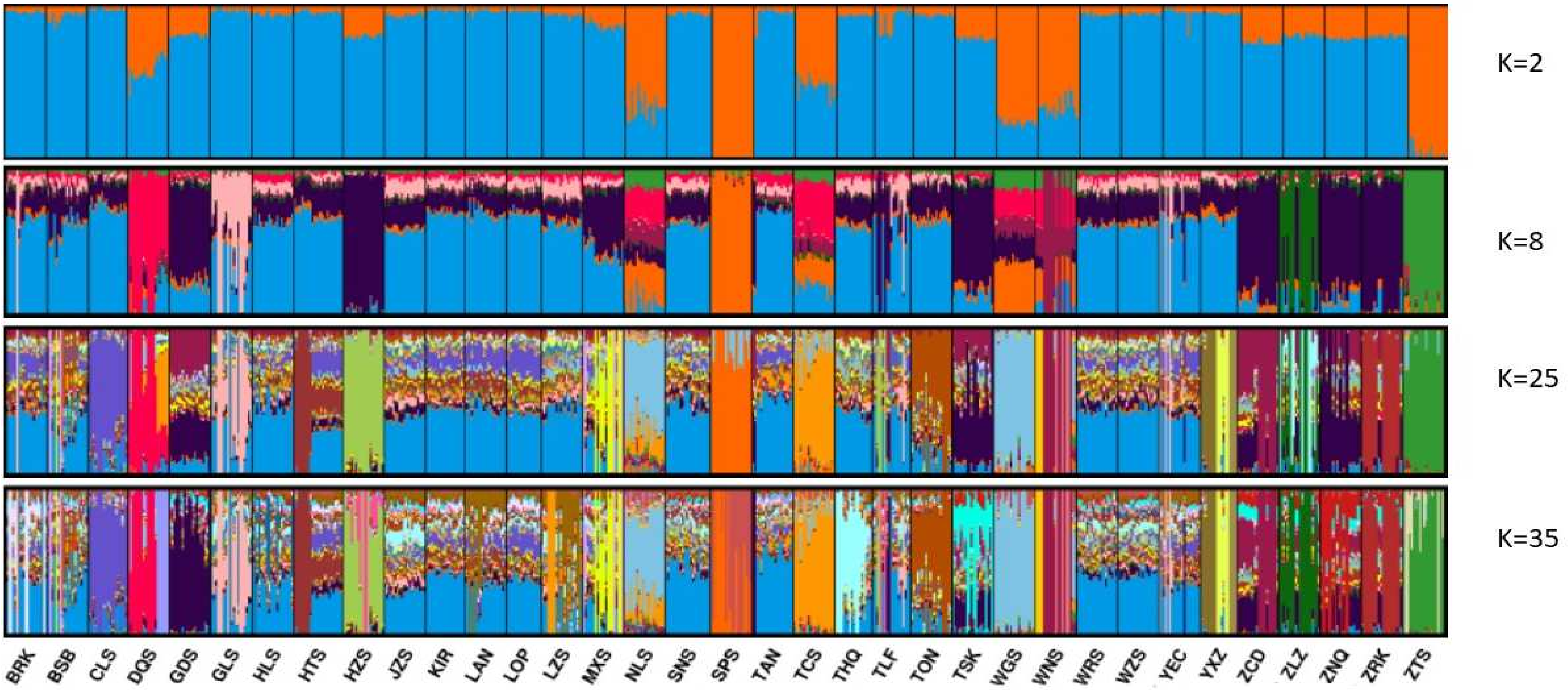

Sheep dataset : China

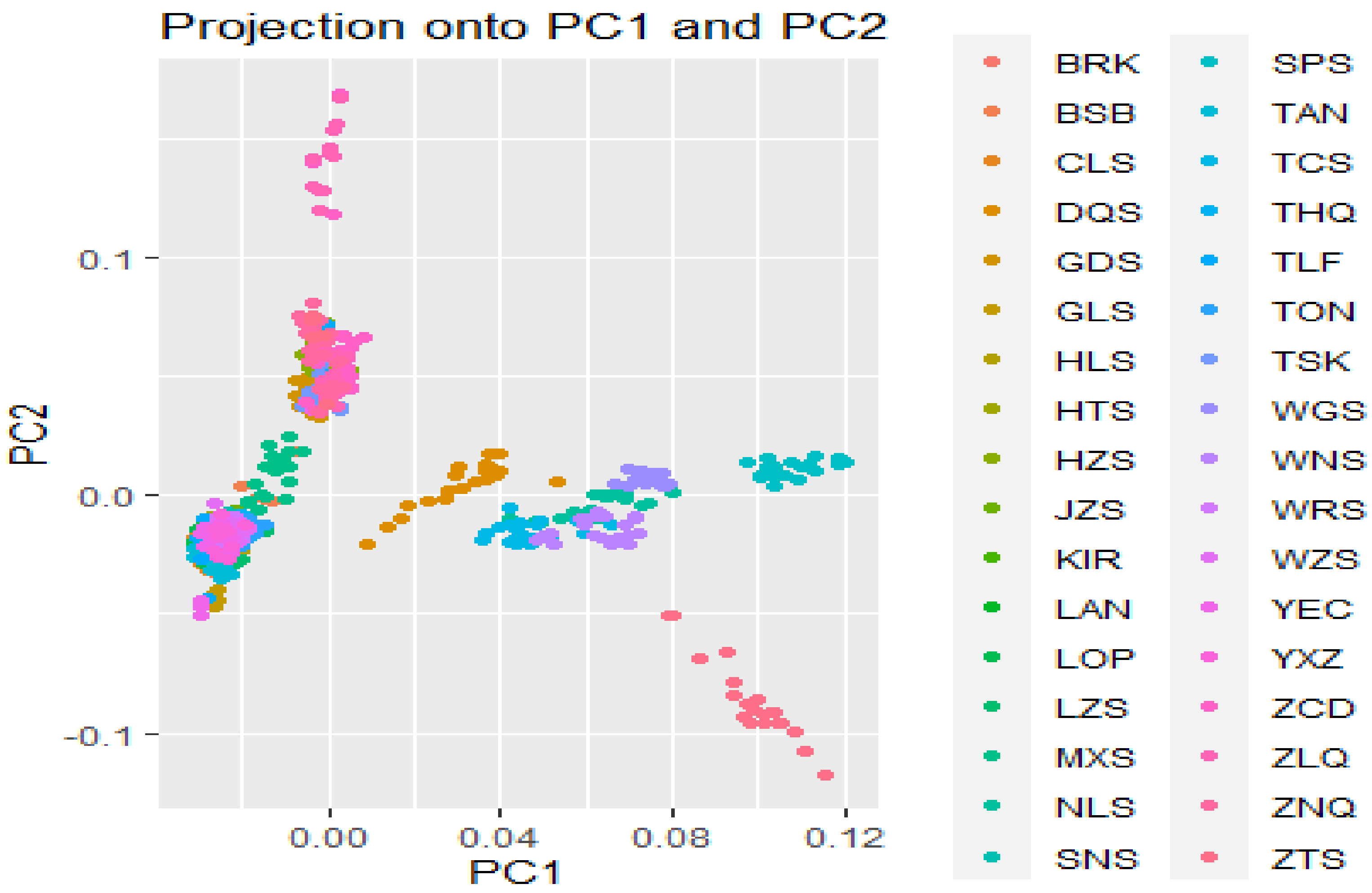

Sheep dataset : China

	Q1	Q2	Q3	Q4	Q5	Q6	Q7	Q8	Q9	Q10	Q11	Q12	Q13	Q14	Q15	Q16	Q17	Q18	Q19	Q20
AKD	0.18	0.00	0.00	0.18	0.00	0.05	0.00	0.00	0.00	0.00	0.02	0.00	0.01	0.01	0.00	0.02	0.34	0.00	0.00	0.17
ADI	0.00	0.01	0.00	0.00	0.00	0.03	0.01	0.00	0.01	0.10	0.21	0.60	0.00	0.01	0.00	0.00	0.00	0.02	0.00	0.00
AKR	0.58	0.00	0.00	0.01	0.01	0.10	0.01	0.00	0.01	0.01	0.00	0.00	0.04	0.01	0.02	0.01	0.01	0.00	0.00	0.16
ARB	0.00	0.01	0.01	0.01	0.01	0.04	0.02	0.00	0.01	0.81	0.06	0.00	0.01	0.00	0.00	0.00	0.01	0.01	0.01	0.01
BHS	0.00	0.00	0.00	0.00	0.00	0.97	0.00	0.00	0.00	0.00	0.00	0.00	0.00	0.00	0.00	0.00	0.00	0.00	0.00	0.00
BON	0.00	0.00	0.00	0.00	0.00	0.00	0.00	0.00	0.00	0.00	0.00	0.99	0.00	0.00	0.00	0.00	0.00	0.00	0.01	0.00
DOY	0.00	0.00	0.00	0.00	0.00	0.01	0.01	0.01	0.00	0.01	0.85	0.05	0.00	0.01	0.00	0.00	0.00	0.02	0.00	0.00
FRT	0.01	0.01	0.13	0.02	0.03	0.02	0.01	0.01	0.05	0.01	0.05	0.02	0.02	0.02	0.01	0.08	0.01	0.01	0.00	0.50
GGD	0.00	0.05	0.03	0.00	0.00	0.01	0.00	0.01	0.18	0.00	0.00	0.11	0.01	0.56	0.01	0.02	0.00	0.00	0.00	0.00
HRI	0.00	0.01	0.00	0.00	0.00	0.01	0.00	0.00	0.00	0.00	0.00	0.00	0.64	0.00	0.21	0.01	0.01	0.00	0.09	0.00
HOR	0.00	0.01	0.01	0.00	0.01	0.00	0.01	0.00	0.57	0.10	0.18	0.02	0.00	0.03	0.00	0.02	0.00	0.01	0.00	0.03
KSH	0.01	0.00	0.00	0.00	0.00	0.01	0.00	0.00	0.00	0.00	0.00	0.00	0.44	0.00	0.18	0.00	0.00	0.00	0.33	0.00
KEF	0.74	0.00	0.00	0.01	0.00	0.16	0.01	0.01	0.01	0.00	0.00	0.00	0.03	0.00	0.02	0.01	0.01	0.00	0.00	0.00
KID	0.00	0.33	0.03	0.00	0.00	0.00	0.00	0.00	0.14	0.00	0.00	0.06	0.00	0.41	0.00	0.00	0.00	0.00	0.00	0.00
LOY	0.00	0.00	0.00	0.00	0.00	0.00	0.00	0.00	0.00	0.62	0.01	0.03	0.00	0.00	0.00	0.00	0.00	0.33	0.00	0.00
EMZA	0.01	0.01	0.01	0.02	0.01	0.02	0.01	0.02	0.00	0.01	0.00	0.01	0.00	0.01	0.01	0.01	0.01	0.01	0.01	0.85
MNZ	0.02	0.01	0.01	0.02	0.02	0.01	0.01	0.02	0.01	0.02	0.00	0.02	0.02	0.03	0.01	0.02	0.02	0.00	0.00	0.85
SHG	0.05	0.00	0.00	0.01	0.00	0.01	0.24	0.00	0.01	0.42	0.22	0.00	0.00	0.00	0.00	0.00	0.00	0.01	0.00	0.01
WSH	0.01	0.01	0.37	0.01	0.17	0.00	0.00	0.07	0.08	0.00	0.04	0.02	0.00	0.02	0.00	0.10	0.00	0.01	0.00	0.08
WOL	0.02	0.01	0.03	0.02	0.01	0.01	0.01	0.03	0.02	0.00	0.10	0.00	0.00	0.00	0.01	0.23	0.01	0.01	0.00	0.47

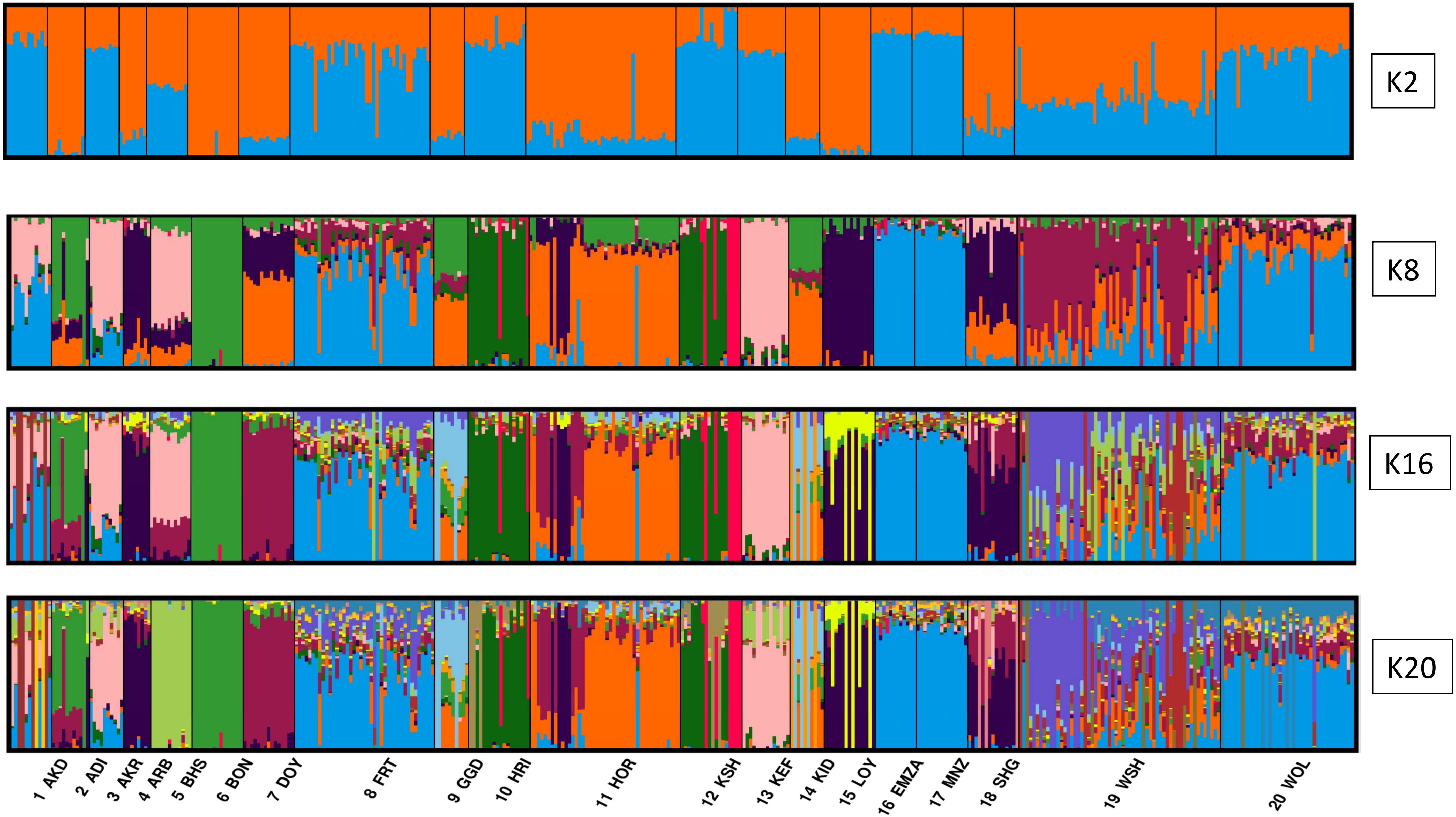

Sheep dataset : Ethiopia

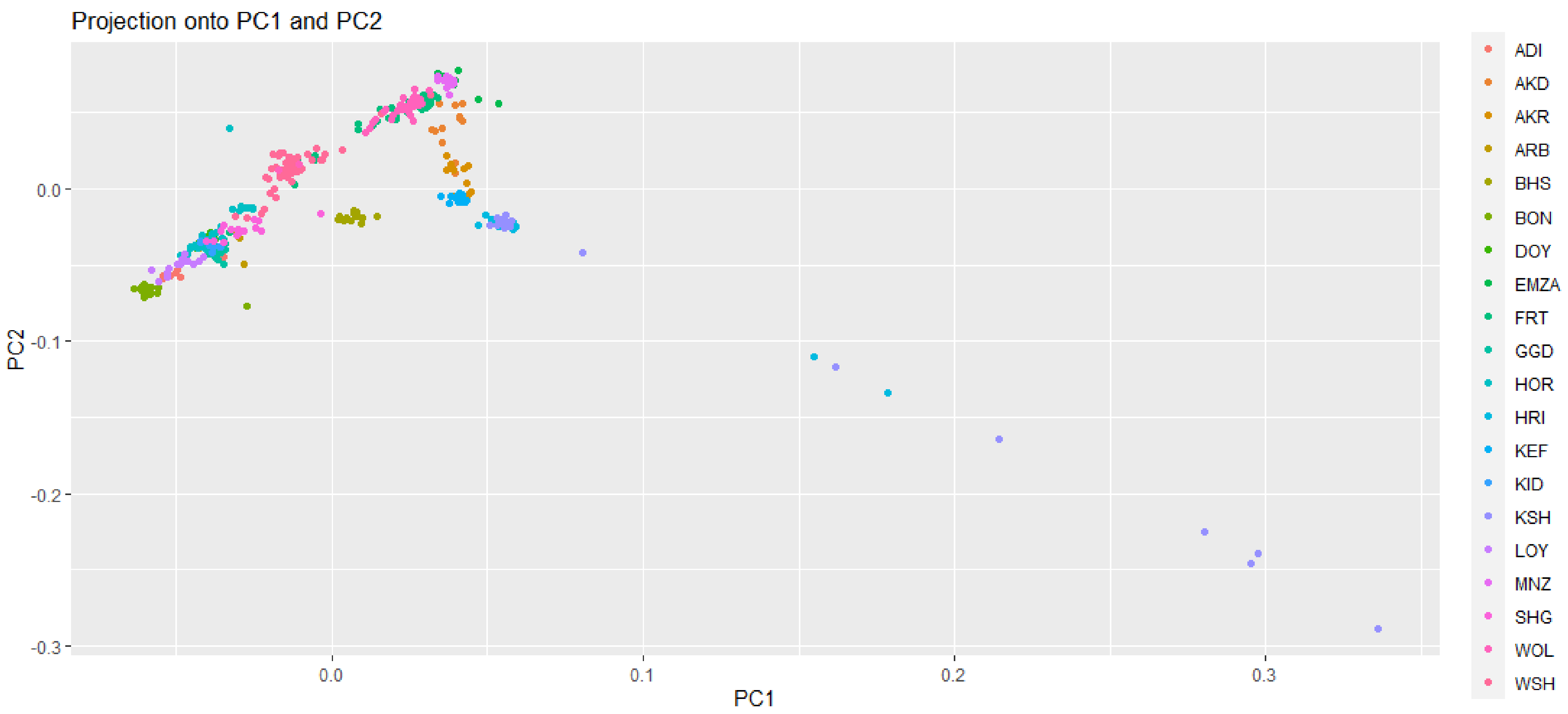

Sheep dataset : Ethiopia

	Q1	Q2	Q3	Q4	Q5	Q6	Q7	Q8	Q9	Q10	Q11	Q12	Q13	Q14	Q15	Q16	Q17	Q18	Q19	Q20	Q21	Q22	Q23	Q24	Q25
BERmean	0.00	0.00	0.00	0.00	0.00	0.00	0.00	0.00	0.99	0.00	0.00	0.00	0.00	0.00	0.00	0.00	0.00	0.00	0.00	0.00	0.00	0.00	0.00	0.00	0.00
BMCmean	0.00	0.00	0.00	0.00	0.00	0.00	0.00	0.01	0.01	0.00	0.01	0.01	0.01	0.01	0.01	0.00	0.85	0.01	0.00	0.00	0.00	0.01	0.00	0.00	0.01
CDLmean	0.00	0.00	0.00	0.00	0.00	0.00	0.00	0.00	0.00	0.00	0.00	0.00	0.01	0.00	0.00	0.00	0.00	0.00	0.00	0.00	0.00	0.97	0.00	0.00	0.00
CHAmean	0.00	0.96	0.00	0.00	0.00	0.00	0.00	0.00	0.00	0.02	0.00	0.00	0.00	0.00	0.00	0.00	0.00	0.00	0.00	0.00	0.00	0.00	0.00	0.00	0.00
CHRmean	0.00	0.00	0.00	0.00	0.00	0.00	0.00	0.00	0.00	0.00	0.00	0.00	0.00	0.00	0.00	0.00	0.00	0.00	0.98	0.00	0.00	0.00	0.00	0.00	0.00
IDFmean	0.00	0.00	0.00	0.00	0.00	0.00	0.00	0.00	0.02	0.00	0.00	0.00	0.00	0.00	0.00	0.00	0.00	0.00	0.00	0.00	0.00	0.00	0.97	0.00	0.00
LACmean	0.00	0.00	0.00	0.00	0.00	0.00	0.00	0.00	0.00	0.00	0.00	0.00	0.01	0.91	0.03	0.00	0.01	0.00	0.00	0.00	0.00	0.00	0.00	0.00	0.00
LAMmean	0.03	0.00	0.01	0.00	0.00	0.00	0.02	0.00	0.01	0.00	0.00	0.00	0.01	0.10	0.78	0.01	0.00	0.00	0.00	0.00	0.00	0.00	0.00	0.00	0.01
LIMmean	0.00	0.00	0.00	0.00	0.00	0.00	0.00	0.00	0.00	0.00	0.00	0.00	0.00	0.00	0.00	0.00	0.00	0.00	0.00	0.00	0.00	0.00	0.00	0.00	0.98
MERmean	0.01	0.00	0.01	0.00	0.01	0.00	0.00	0.00	0.00	0.00	0.00	0.00	0.01	0.01	0.00	0.91	0.00	0.00	0.00	0.01	0.00	0.00	0.01	0.00	0.00
MOUmean	0.01	0.00	0.06	0.00	0.00	0.01	0.01	0.00	0.03	0.00	0.01	0.01	0.42	0.01	0.00	0.32	0.00	0.01	0.00	0.01	0.00	0.01	0.03	0.00	0.02
MTRmean	0.00	0.00	0.00	0.00	0.00	0.00	0.00	0.00	0.00	0.00	0.00	0.97	0.00	0.00	0.00	0.00	0.00	0.00	0.00	0.00	0.00	0.00	0.00	0.00	0.00
NVEmean	0.93	0.00	0.00	0.01	0.00	0.00	0.00	0.00	0.00	0.00	0.00	0.00	0.01	0.00	0.00	0.00	0.01	0.00	0.00	0.00	0.00	0.00	0.00	0.00	0.01
OUEmean	0.00	0.00	0.00	0.00	0.00	0.00	0.00	0.00	0.00	0.00	0.98	0.00	0.00	0.00	0.00	0.00	0.00	0.00	0.00	0.00	0.00	0.00	0.00	0.00	0.00
PASmean	0.02	0.00	0.00	0.00	0.00	0.00	0.01	0.00	0.00	0.01	0.00	0.01	0.85	0.01	0.02	0.01	0.02	0.00	0.00	0.00	0.00	0.01	0.00	0.00	0.01
RAMmean	0.00	0.00	0.00	0.00	0.54	0.00	0.00	0.00	0.00	0.00	0.00	0.00	0.00	0.00	0.00	0.00	0.00	0.00	0.00	0.00	0.00	0.00	0.00	0.46	0.00
RAVmean	0.01	0.00	0.01	0.00	0.00	0.00	0.90	0.00	0.00	0.00	0.00	0.00	0.01	0.00	0.01	0.01	0.01	0.00	0.00	0.00	0.00	0.01	0.00	0.00	0.01
RMNmean	0.00	0.00	0.00	0.00	0.00	0.00	0.00	0.00	0.32	0.00	0.00	0.00	0.00	0.00	0.00	0.00	0.00	0.55	0.00	0.01	0.00	0.00	0.11	0.00	0.00
ROMmean	0.00	0.00	0.00	0.00	0.00	0.00	0.00	0.00	0.00	0.00	0.00	0.00	0.00	0.00	0.00	0.00	0.00	1.00	0.00	0.00	0.00	0.00	0.00	0.00	0.00
ROUmean	0.00	0.00	0.00	0.01	0.00	0.95	0.00	0.00	0.00	0.01	0.00	0.00	0.00	0.00	0.00	0.00	0.00	0.00	0.00	0.01	0.00	0.00	0.00	0.00	0.00
RWEmean	0.00	0.00	0.00	0.00	0.00	0.00	0.00	0.00	0.00	0.01	0.00	0.00	0.00	0.00	0.00	0.00	0.00	0.00	0.00	0.97	0.00	0.00	0.00	0.00	0.00
SUFmean	0.00	0.00	0.00	0.99	0.00	0.00	0.00	0.00	0.00	0.00	0.00	0.00	0.00	0.00	0.00	0.00	0.00	0.00	0.00	0.00	0.00	0.00	0.00	0.00	0.00
TARmean	0.01	0.00	0.85	0.00	0.00	0.00	0.00	0.00	0.01	0.01	0.01	0.01	0.00	0.01	0.01	0.02	0.01	0.01	0.00	0.00	0.00	0.00	0.00	0.00	0.00
TEXmean	0.00	0.00	0.00	0.00	0.00	0.00	0.00	0.00	0.00	0.00	0.00	0.00	0.00	0.00	0.00	0.00	0.00	0.00	0.00	0.00	0.99	0.00	0.00	0.00	0.00
VENmean	0.00	0.01	0.00	0.00	0.00	0.00	0.00	0.00	0.00	0.97	0.00	0.00	0.00	0.00	0.00	0.00	0.00	0.00	0.00	0.00	0.00	0.00	0.00	0.00	0.00

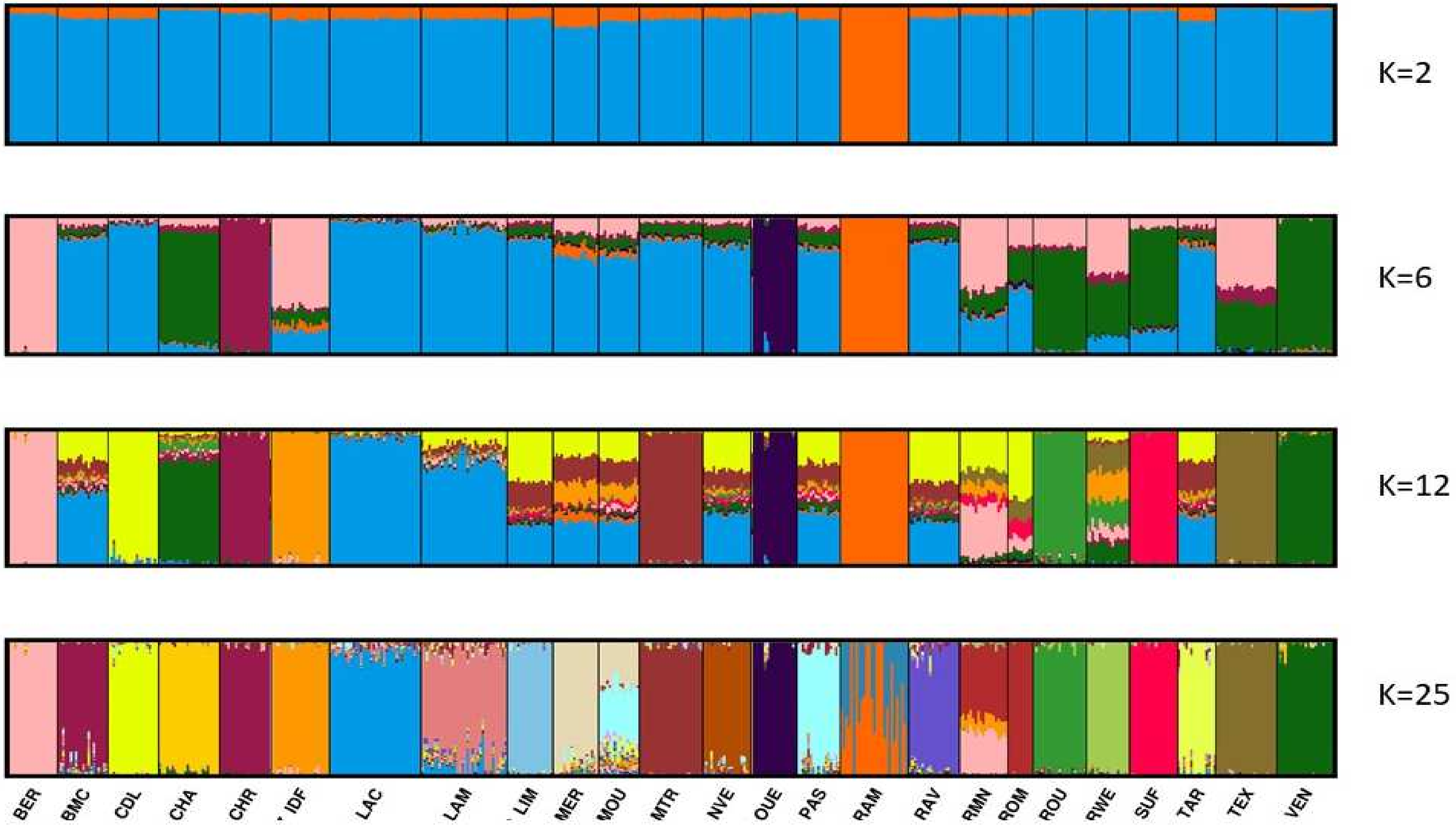

Sheep dataset : France

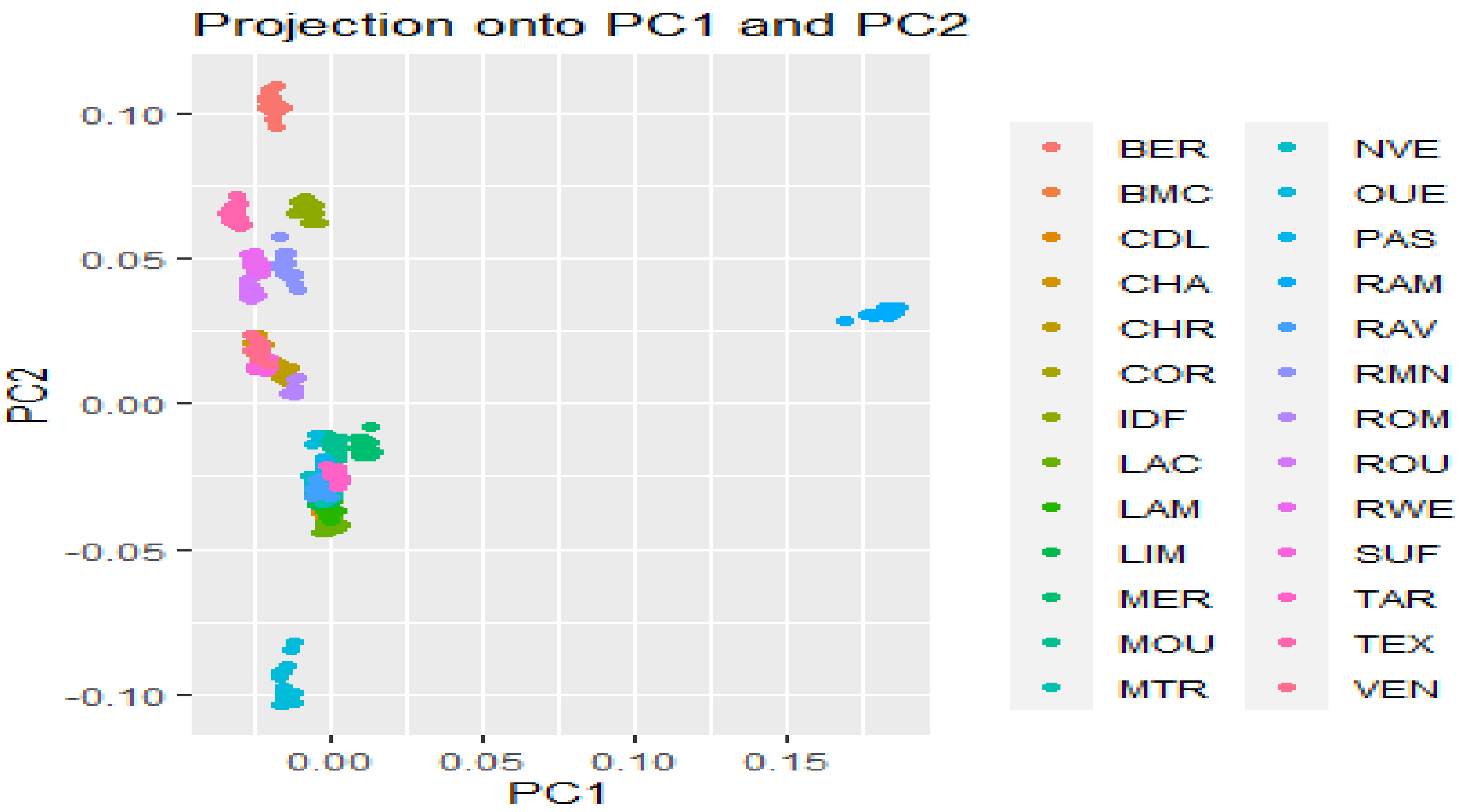
Projection onto PC1 and PC2
0.10
0.05
0.00
-0.05
-0.10
PC2
0.00
0.05
0.10
0.15
PC1
BER
BMC
CDL
CHA
CHR
COR
IDF
LAC
LAM
LIM
MER
MOU
MTR
NVE
OUE
PAS
RAM
RAV
RMN
ROM
ROU
RWE
SUF
TAR
TEX
VEN

	Q1	Q2	Q3	Q4	Q5	Q6	Q7	Q8	Q9	Q10	Q11	Q12	Q13	Q14
BHP	0.00	0.00	0.00	0.98	0.00	0.00	0.00	0.00	0.00	0.00	0.00	0.01	0.00	0.00
DAM	0.01	0.00	0.00	0.02	0.01	0.00	0.25	0.00	0.01	0.00	0.00	0.68	0.01	0.00
DOP	0.16	0.01	0.01	0.04	0.61	0.00	0.00	0.00	0.01	0.01	0.00	0.10	0.02	0.02
KAR	0.00	0.53	0.00	0.01	0.01	0.18	0.00	0.14	0.00	0.00	0.13	0.01	0.00	0.00
MMA	0.02	0.03	0.02	0.09	0.45	0.01	0.02	0.02	0.03	0.01	0.01	0.26	0.01	0.01
NQA	0.60	0.00	0.01	0.02	0.02	0.01	0.01	0.01	0.03	0.02	0.00	0.22	0.02	0.02
NGU	0.00	0.00	0.00	0.01	0.00	0.00	0.08	0.00	0.00	0.00	0.00	0.02	0.89	0.00
PED	0.00	0.00	0.00	0.02	0.00	0.00	0.01	0.00	0.93	0.00	0.00	0.03	0.00	0.00
SAFM	0.00	0.00	0.00	0.00	0.00	0.00	0.00	0.00	0.00	0.00	0.00	0.00	0.00	1.00
SAMM	0.00	0.00	0.00	0.00	0.00	0.00	0.00	0.00	0.00	0.98	0.00	0.00	0.00	0.01
BVS	0.01	0.06	0.02	0.00	0.03	0.04	0.00	0.62	0.00	0.01	0.18	0.00	0.00	0.03
GVS	0.01	0.35	0.03	0.02	0.02	0.29	0.00	0.16	0.01	0.01	0.06	0.02	0.00	0.02
WSS	0.00	0.05	0.01	0.00	0.03	0.31	0.00	0.55	0.00	0.00	0.00	0.00	0.00	0.03
WVS	0.00	0.17	0.15	0.00	0.00	0.43	0.00	0.10	0.00	0.00	0.14	0.00	0.00	0.00
ZUL	0.00	0.00	0.00	0.01	0.00	0.00	0.68	0.00	0.01	0.00	0.00	0.23	0.05	0.00

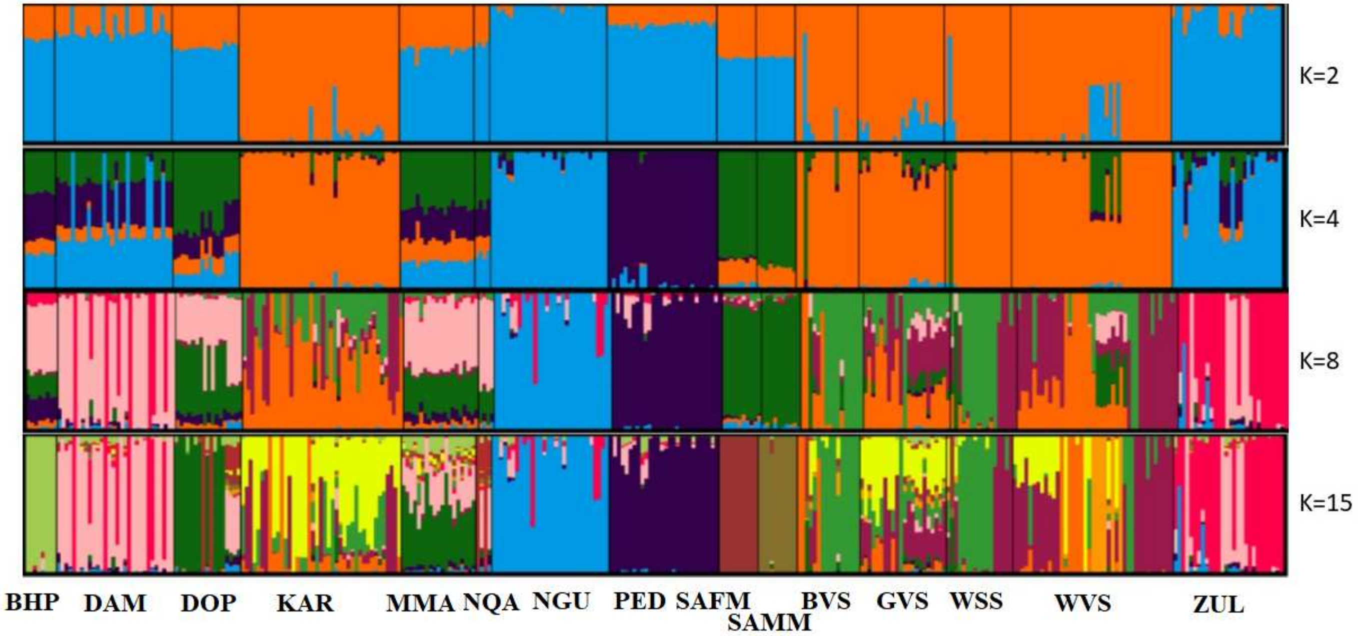

Sheep Dataset : South Africa

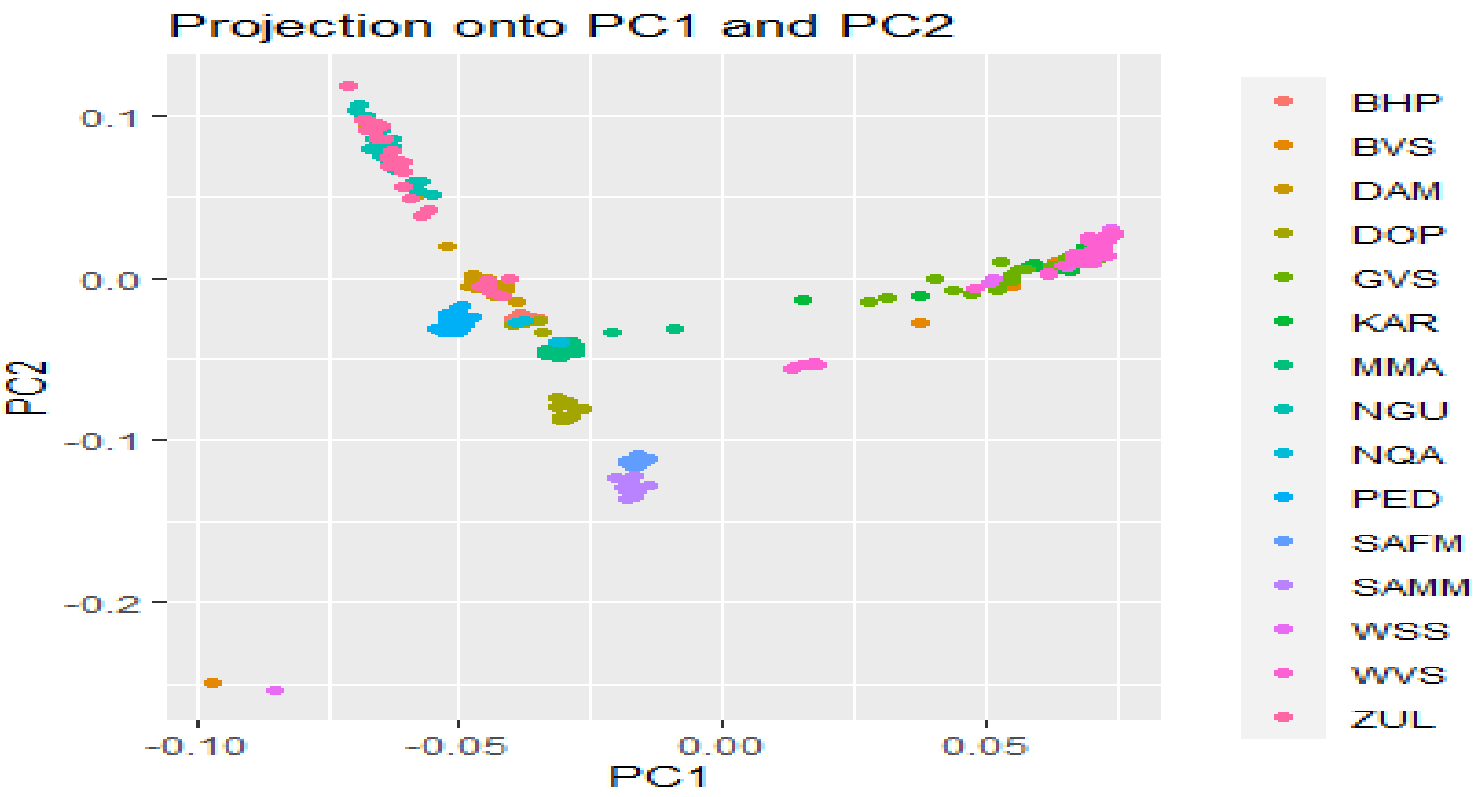

Projection onto PC1 and PC2
0.1
0.0
-0.1
-0.2
PC2
-0.10
-0.05
0.00
0.05
PC1
BHP
BVS
DAM
DOP
GVS
KAR
MMA
NGU
NQA
PED
SAFM
SAMM
WSS
WVS
ZUL

	Q1	Q2	Q3	Q4	Q5	Q6	Q7	Q8	Q9	Q10
CASmean	0.02	0.04	0.05	0.51	0.03	0.03	0.01	0.02	0.28	0.01
LTXmean	0.01	0.00	0.02	0.01	0.00	0.00	0.00	0.60	0.00	0.36
MEEmean	0.03	0.07	0.10	0.04	0.53	0.13	0.02	0.04	0.02	0.01
MERmean	0.02	0.02	0.06	0.01	0.05	0.81	0.01	0.01	0.01	0.01
OJAmean	0.02	0.76	0.07	0.03	0.03	0.02	0.01	0.03	0.02	0.00
RAAmean	0.07	0.17	0.23	0.10	0.12	0.12	0.04	0.09	0.04	0.02
RIPmean	0.04	0.05	0.52	0.03	0.02	0.02	0.28	0.03	0.01	0.01
SAAmean	0.02	0.03	0.10	0.01	0.02	0.01	0.01	0.67	0.02	0.11
SEGmean	0.07	0.17	0.24	0.10	0.13	0.12	0.02	0.08	0.05	0.02
XISmean	0.27	0.12	0.25	0.07	0.07	0.07	0.04	0.08	0.03	0.02

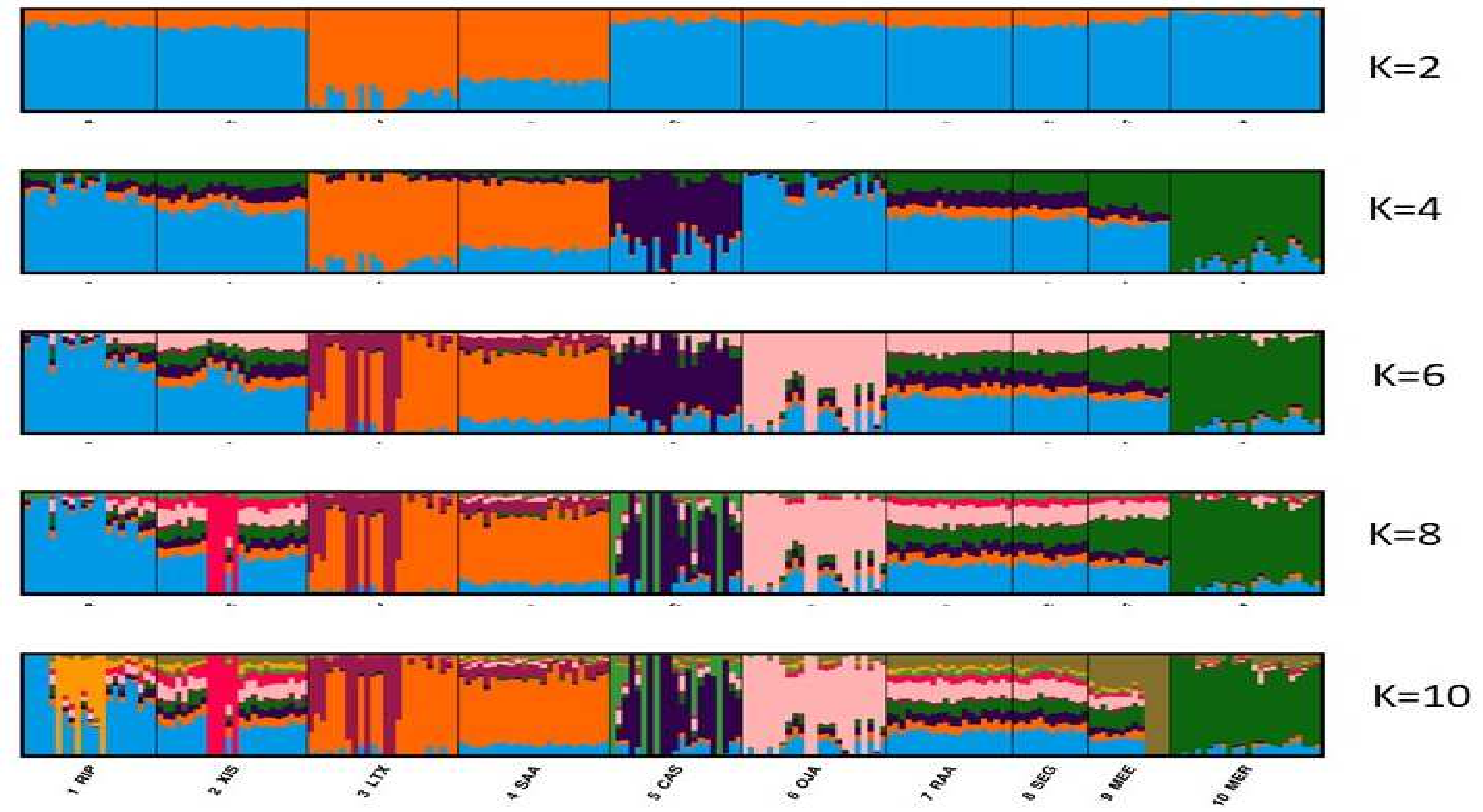

Sheep dataset : Spain

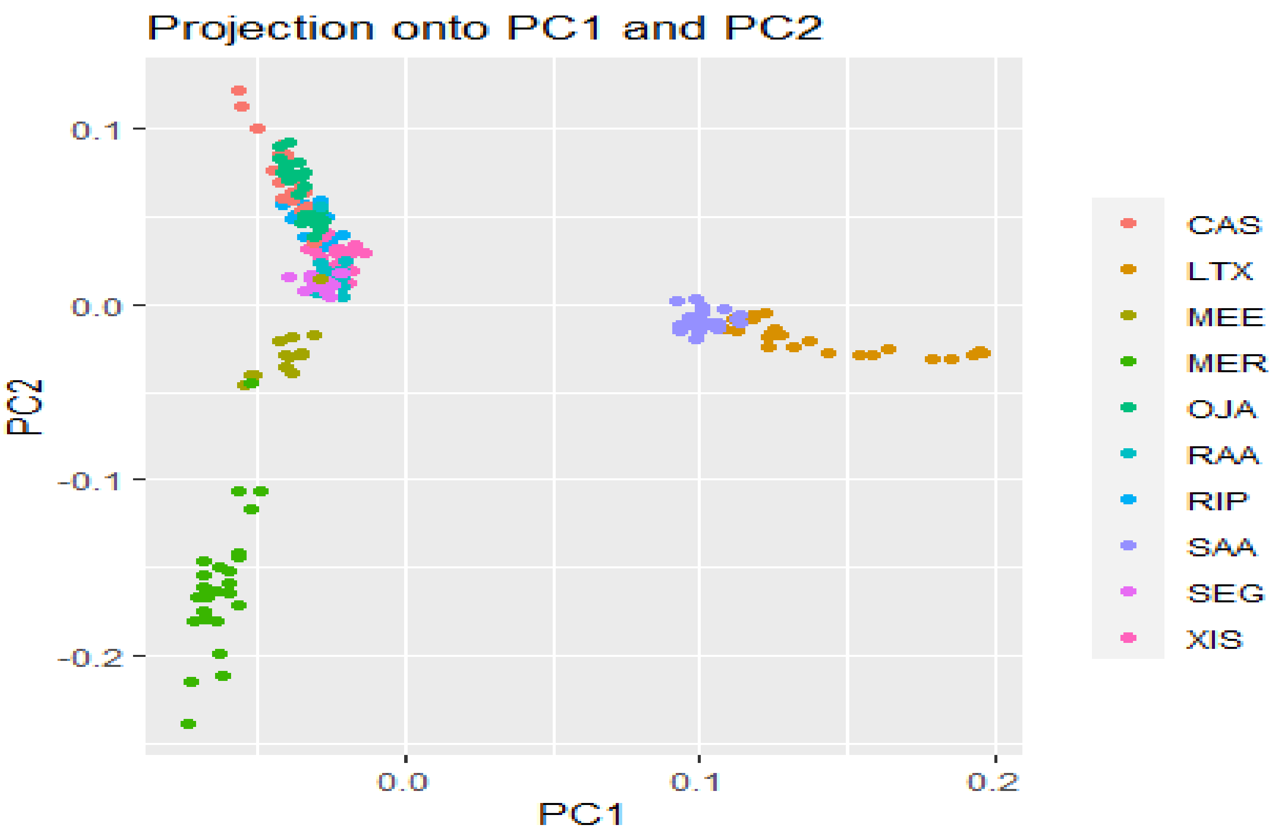

Sheep dataset : Spain

	Q1	Q2	Q3	Q4	Q5	Q6	Q7	Q8	Q9	Q10	Q11	Q12	Q13	Q14	Q15	Q16	Q17	Q18
ALPmean	0.01	0.07	0.01	0.00	0.00	0.86	0.01	0.00	0.00	0.00	0.01	0.01	0.00	0.00	0.01	0.00	0.00	0.01
ALTmean	0.00	0.01	0.00	0.01	0.00	0.00	0.00	0.00	0.01	0.00	0.00	0.01	0.00	0.00	0.9	0.01	0.00	0.02
APPmean	0.02	0.05	0.01	0.01	0.02	0.01	0.01	0.03	0.03	0.01	0.70	0.01	0.00	0.03	0.02	0.02	0.00	0.01
BAGmean	0.04	0.02	0.01	0.03	0.06	0.01	0.01	0.26	0.23	0.02	0.04	0.06	0.01	0.02	0.10	0.04	0.01	0.02
BERmean	0.01	0.83	0.01	0.00	0.01	0.06	0.01	0.01	0.01	0.00	0.01	0.00	0.00	0.02	0.00	0.01	0.00	0.00
BIEmean	0.00	0.85	0.00	0.01	0.01	0.01	0.06	0.01	0.00	0.00	0.00	0.00	0.00	0.01	0.00	0.00	0.00	0.01
COMmean	0.00	0.00	0.01	0.00	0.00	0.00	0.00	0.00	0.92	0.01	0.01	0.00	0.01	0.00	0.00	0.00	0.02	0.00
DELmean	0.00	0.01	0.00	0.00	0.01	0.01	0.01	0.01	0.00	0.01	0.01	0.00	0.00	0.00	0.01	0.01	0.00	0.91
FABmean	0.01	0.04	0.48	0.01	0.01	0.01	0.01	0.01	0.01	0.01	0.01	0.00	0.00	0.39	0.01	0.00	0.00	0.01
GENmean	0.01	0.01	0.00	0.00	0.02	0.01	0.00	0.01	0.01	0.9	0.01	0.00	0.00	0.00	0.02	0.00	0.00	0.00
LATmean	0.02	0.01	0.01	0.01	0.02	0.01	0.01	0.71	0.06	0.02	0.02	0.02	0.01	0.01	0.03	0.02	0.01	0.00
LECmean	0.01	0.10	0.01	0.02	0.01	0.02	0.02	0.02	0.13	0.01	0.02	0.53	0.01	0.01	0.05	0.02	0.01	0.01
MASmean	0.81	0.02	0.01	0.01	0.01	0.01	0.01	0.02	0.01	0.01	0.01	0.02	0.00	0.01	0.02	0.01	0.01	0.01
PINmean	0.03	0.01	0.01	0.05	0.02	0.01	0.02	0.02	0.55	0.03	0.03	0.03	0.04	0.01	0.09	0.03	0.03	0.02
SAMmean	0.00	0.10	0.01	0.00	0.01	0.00	0.82	0.01	0.00	0.00	0.00	0.00	0.00	0.01	0.00	0.00	0.00	0.01
SAWmean	0.00	0.01	0.00	0.86	0.01	0.00	0.01	0.01	0.03	0.01	0.00	0.01	0.01	0.01	0.02	0.01	0.01	0.01
SOPmean	0.02	0.01	0.01	0.03	0.33	0.01	0.03	0.04	0.03	0.1	0.02	0.02	0.01	0.02	0.0	0.32	0.01	0.01
VBEmean	0.00	0.00	0.00	0.01	0.00	0.00	0.00	0.01	0.05	0.00	0.00	0.01	0.54	0.00	0.01	0.00	0.35	0.00

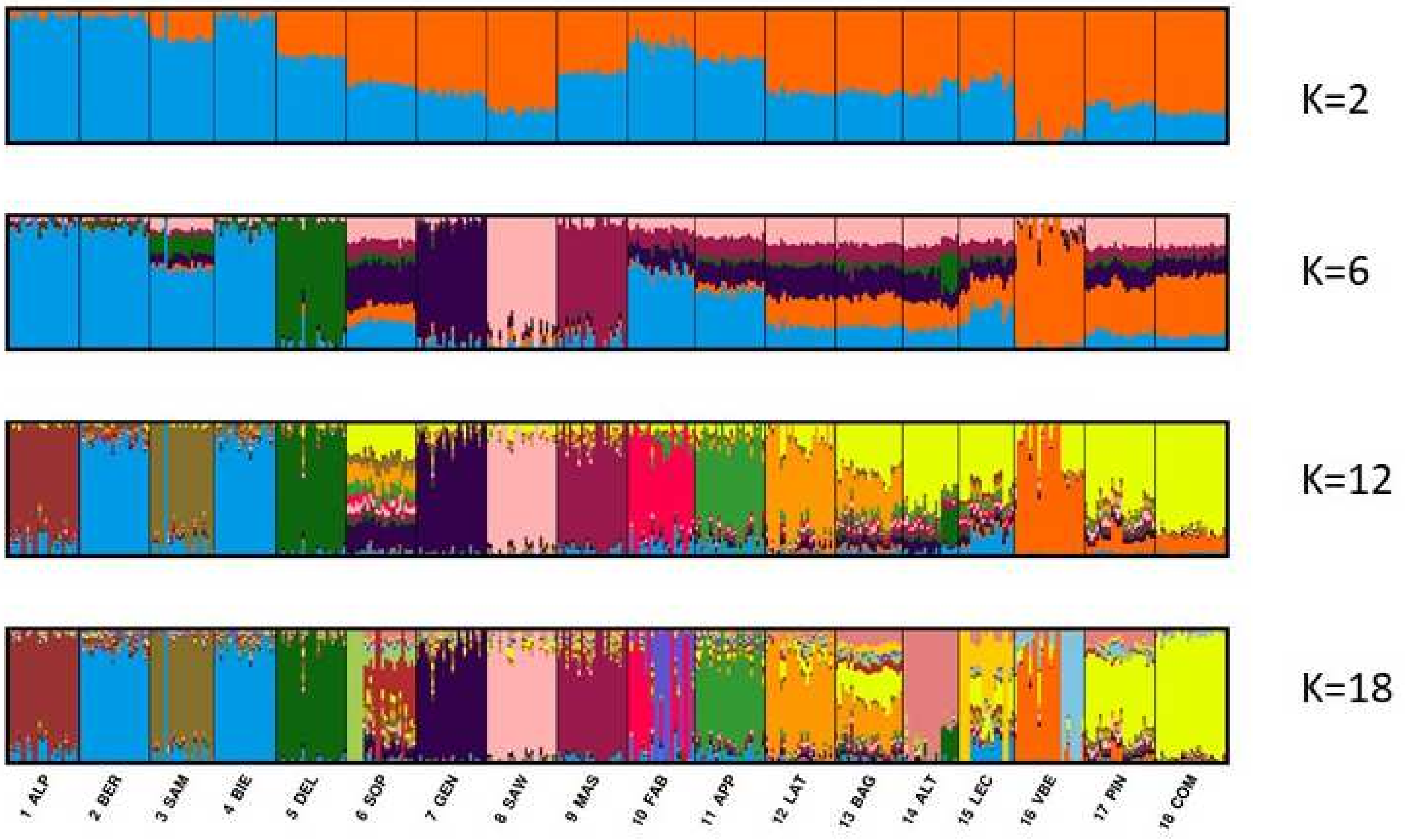

Sheep dataset : Italy

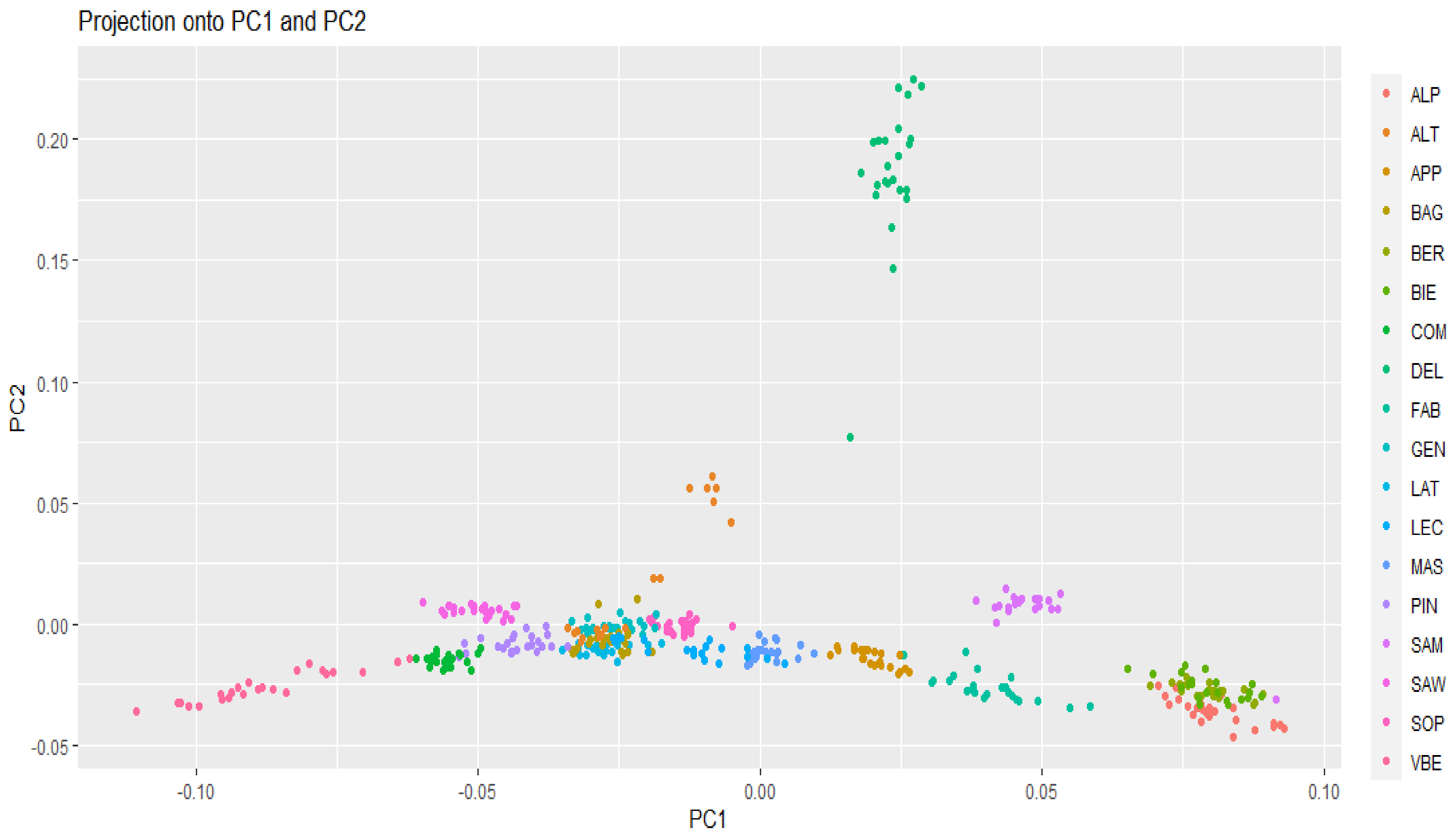

Sheep dataset : Italy

Supplementary Figures 3: Neighbor net graphs, based on Reynold's genetic distances, for goat datasets.

European goat dataset: France, Italy, Spain

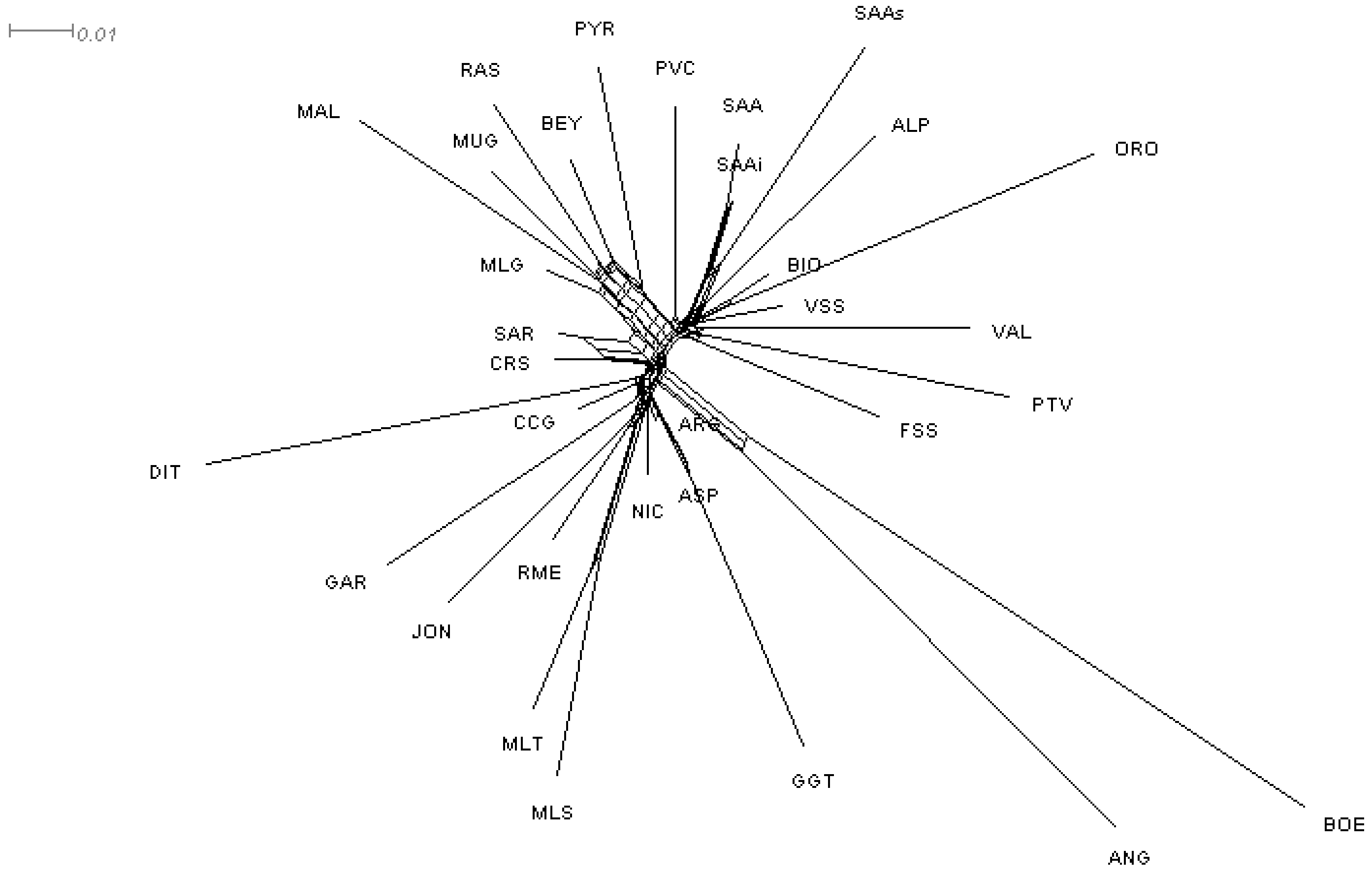

European goat dataset: North Europe

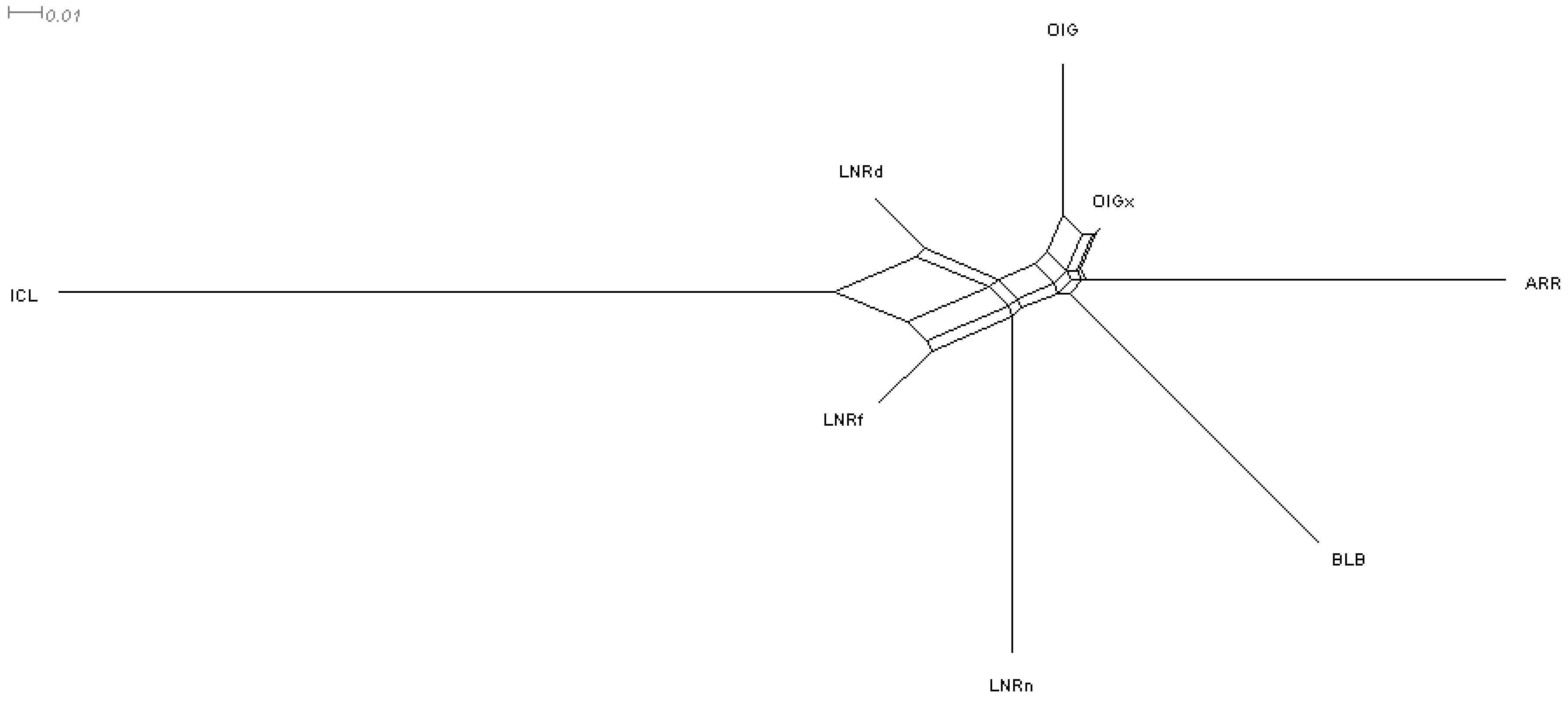

Asian goat dataset

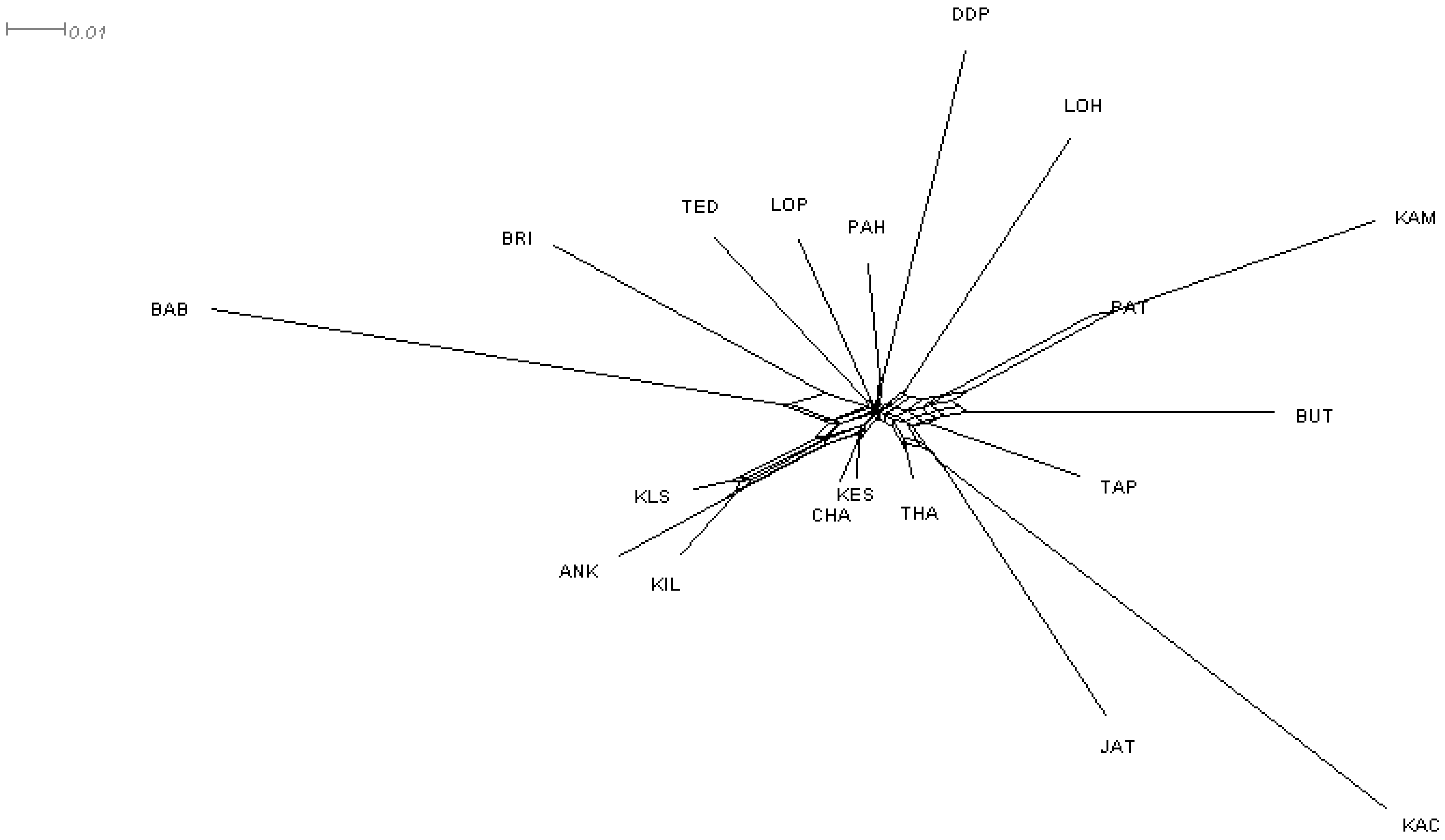

African goat dataset East Africa

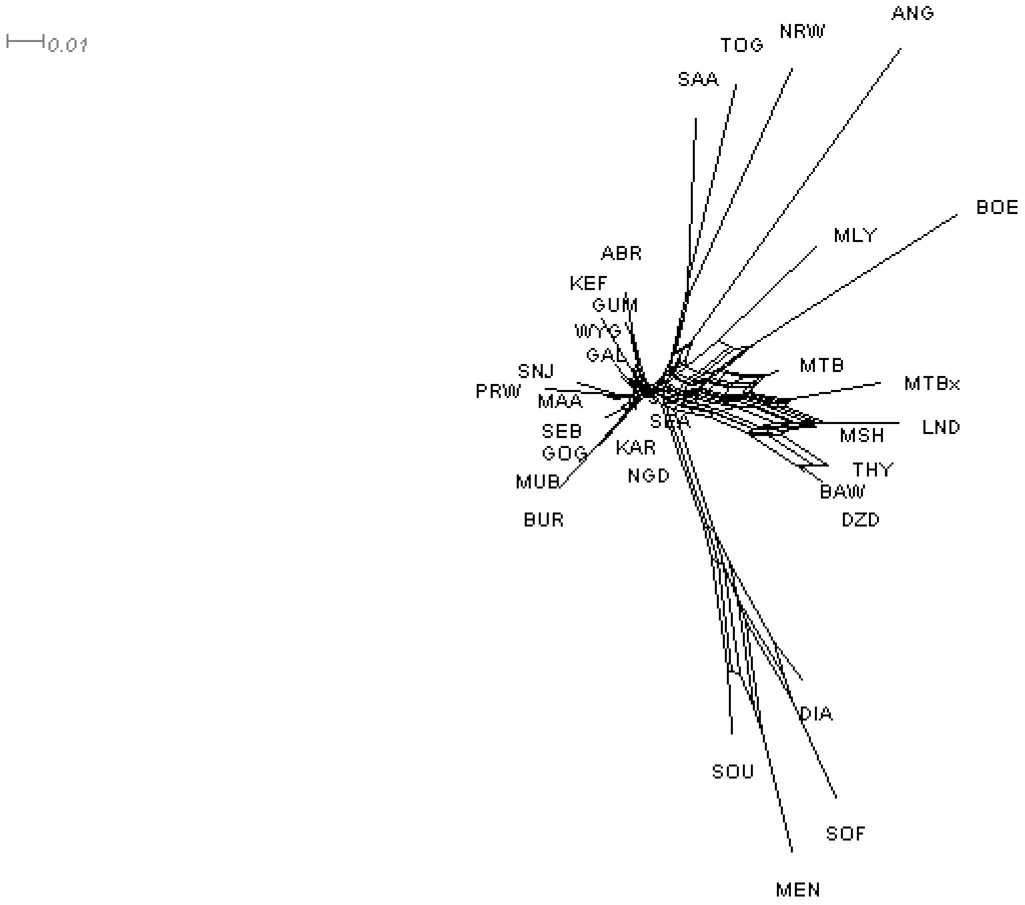

African goat dataset North and West Africa

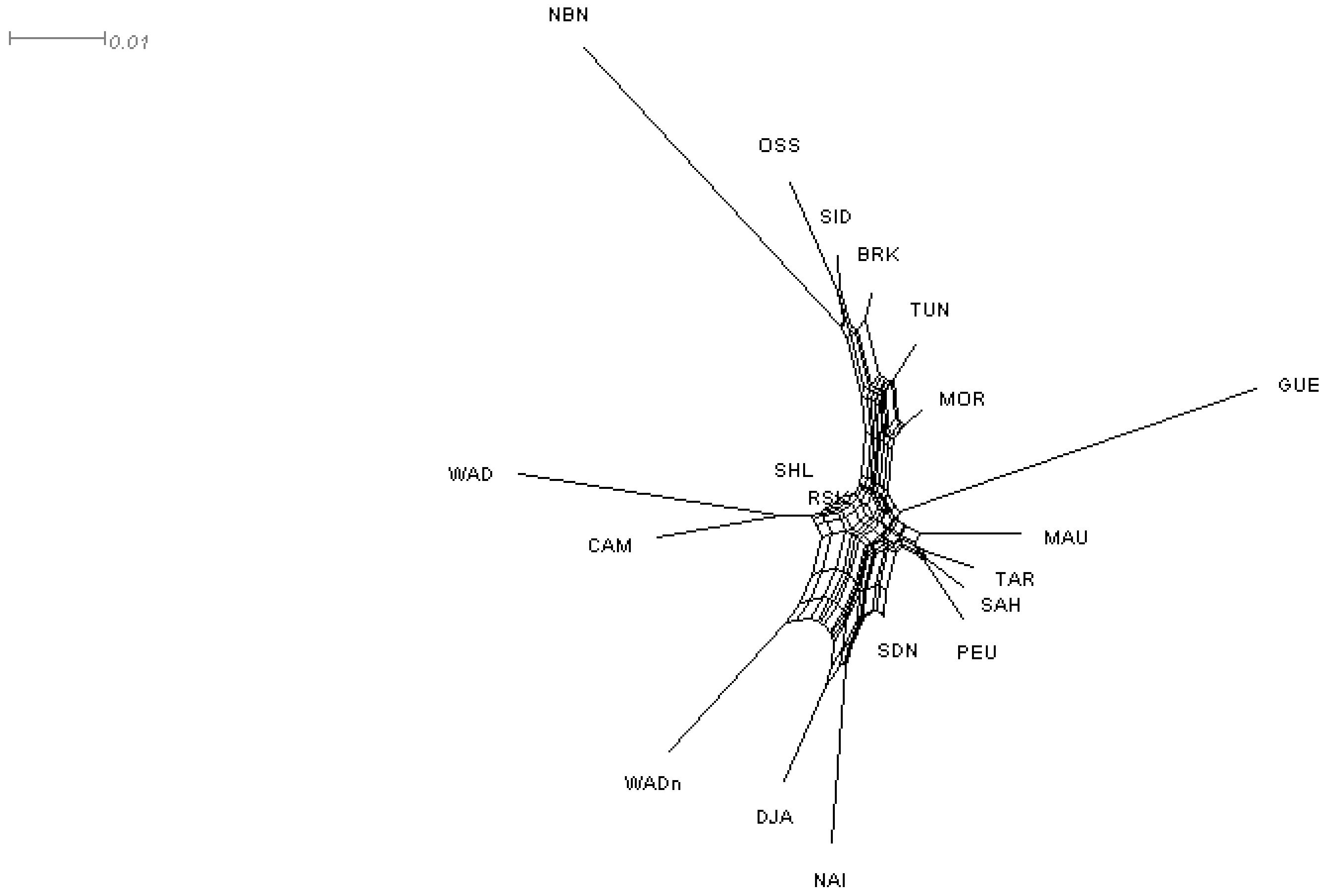

Supplementary Figures 4: Neighbor net graphs, based on Reynold's genetic distances, for sheep datasets.

European sheep dataset: France, Italy, Spain

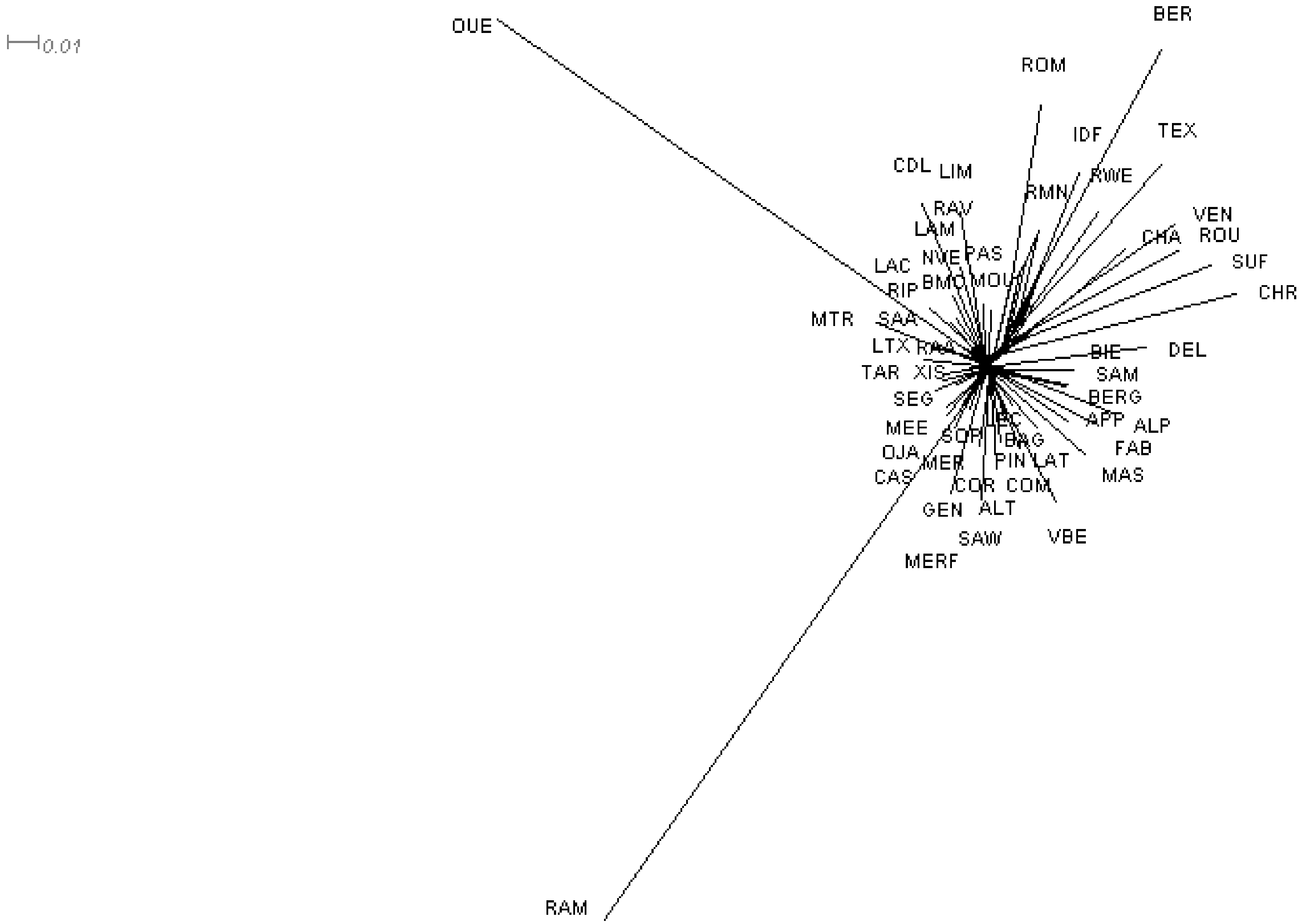

European sheep dataset: Central and Northern Europe

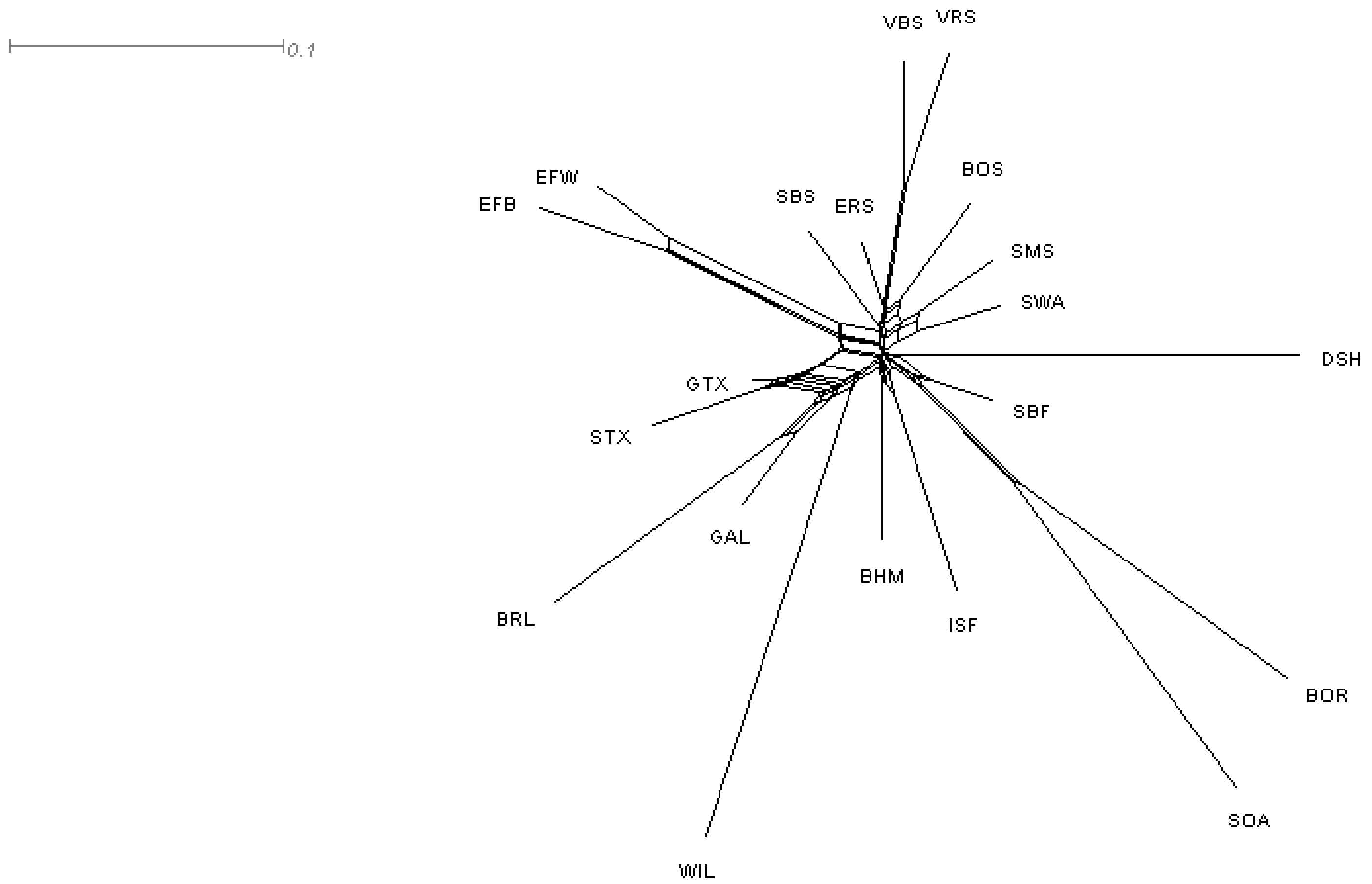

Asian sheep dataset: Southwest Asia

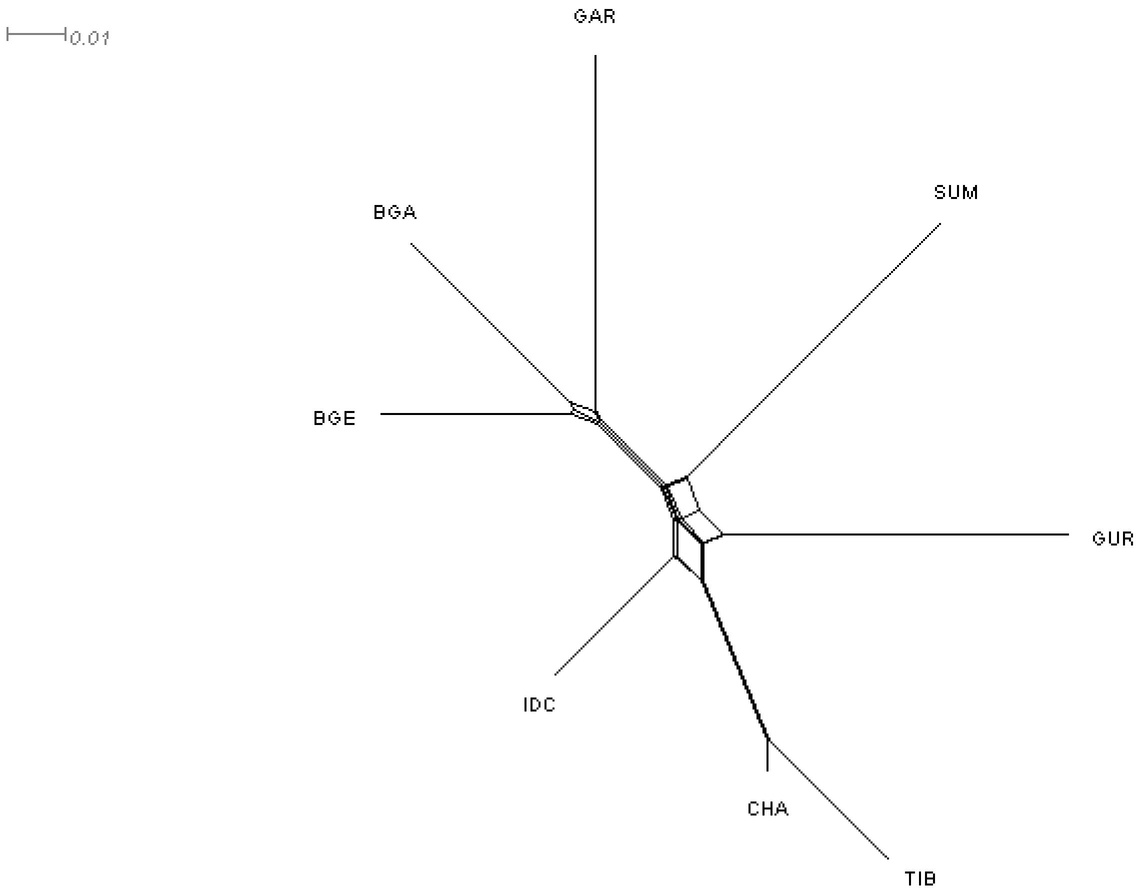

Asian sheep dataset: Pacific Asia

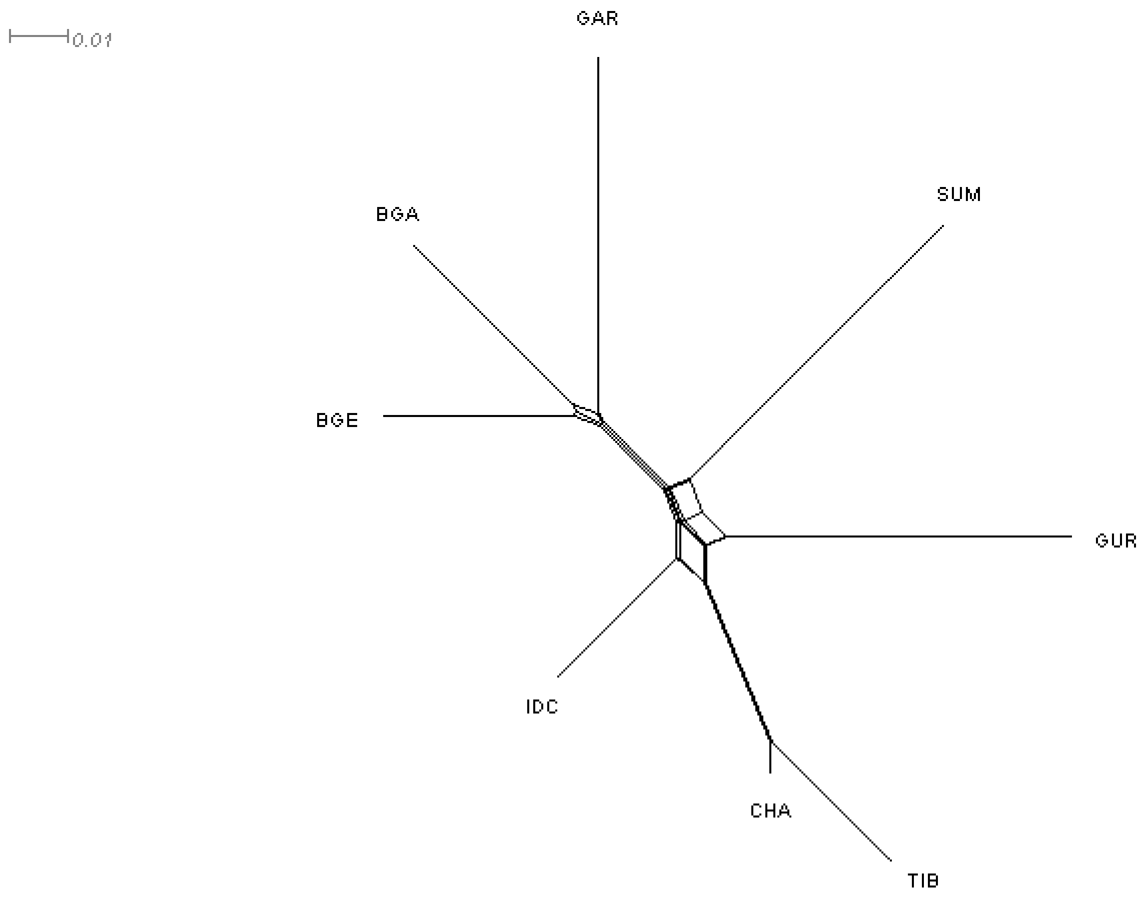

Asian sheep dataset: China

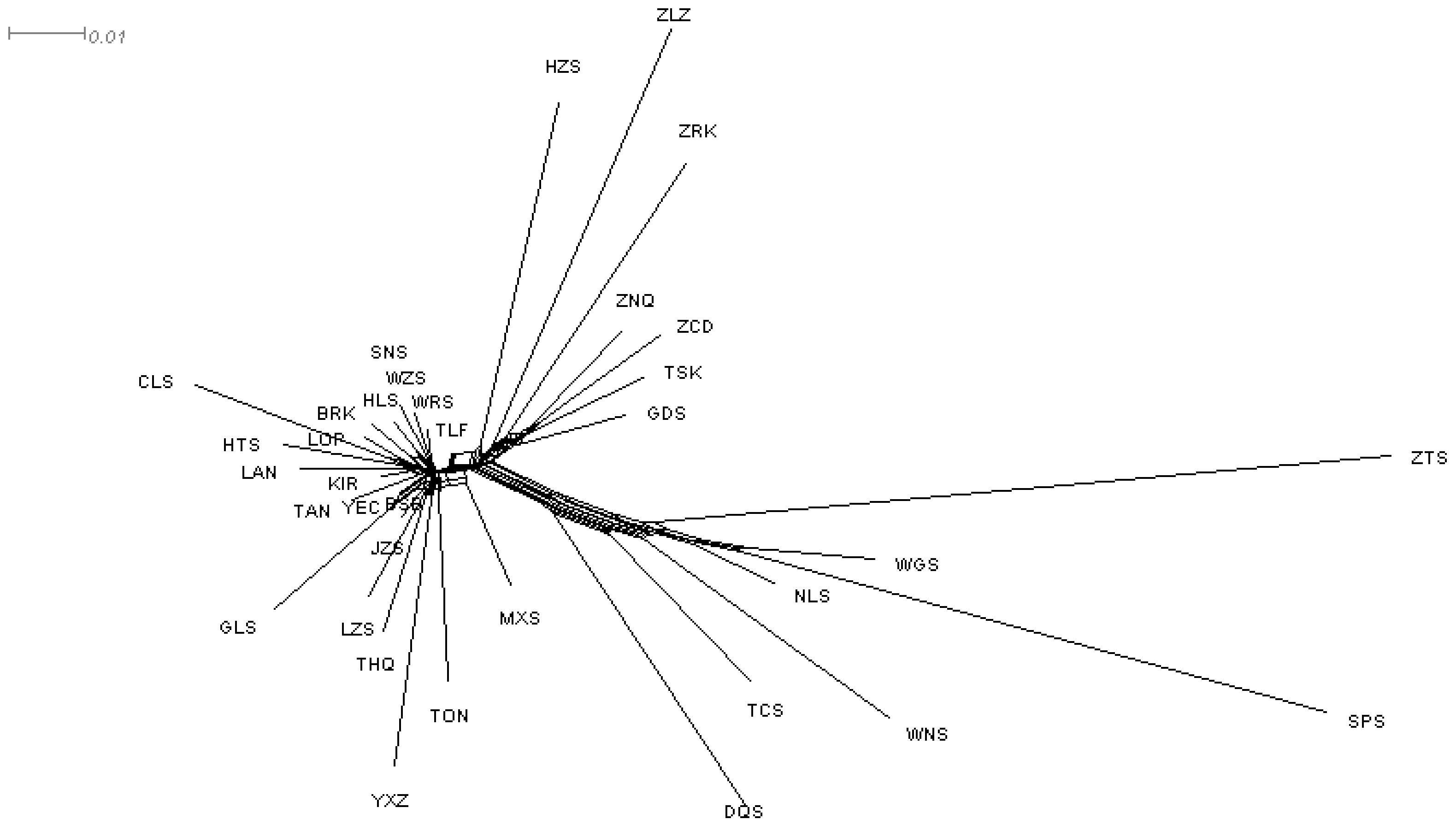

African sheep dataset: Ethiopia

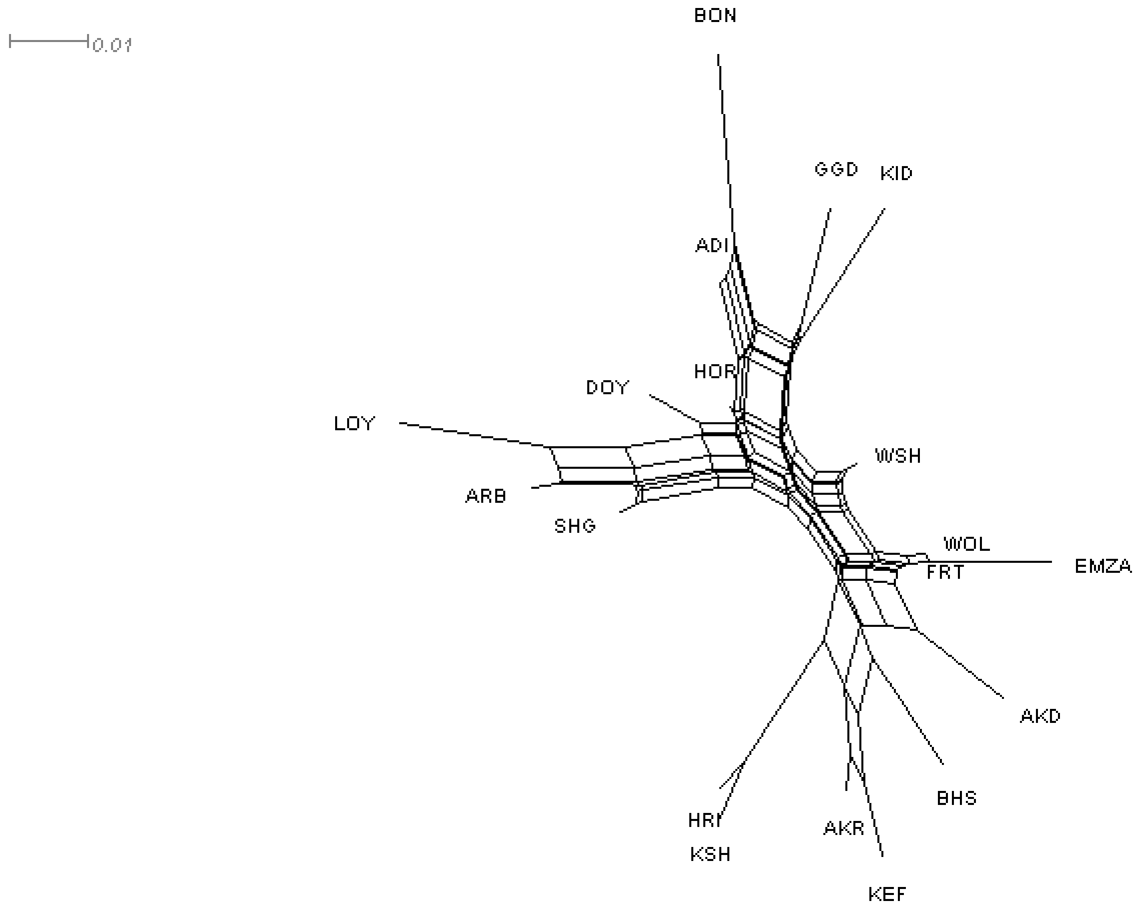

African sheep dataset: South Africa

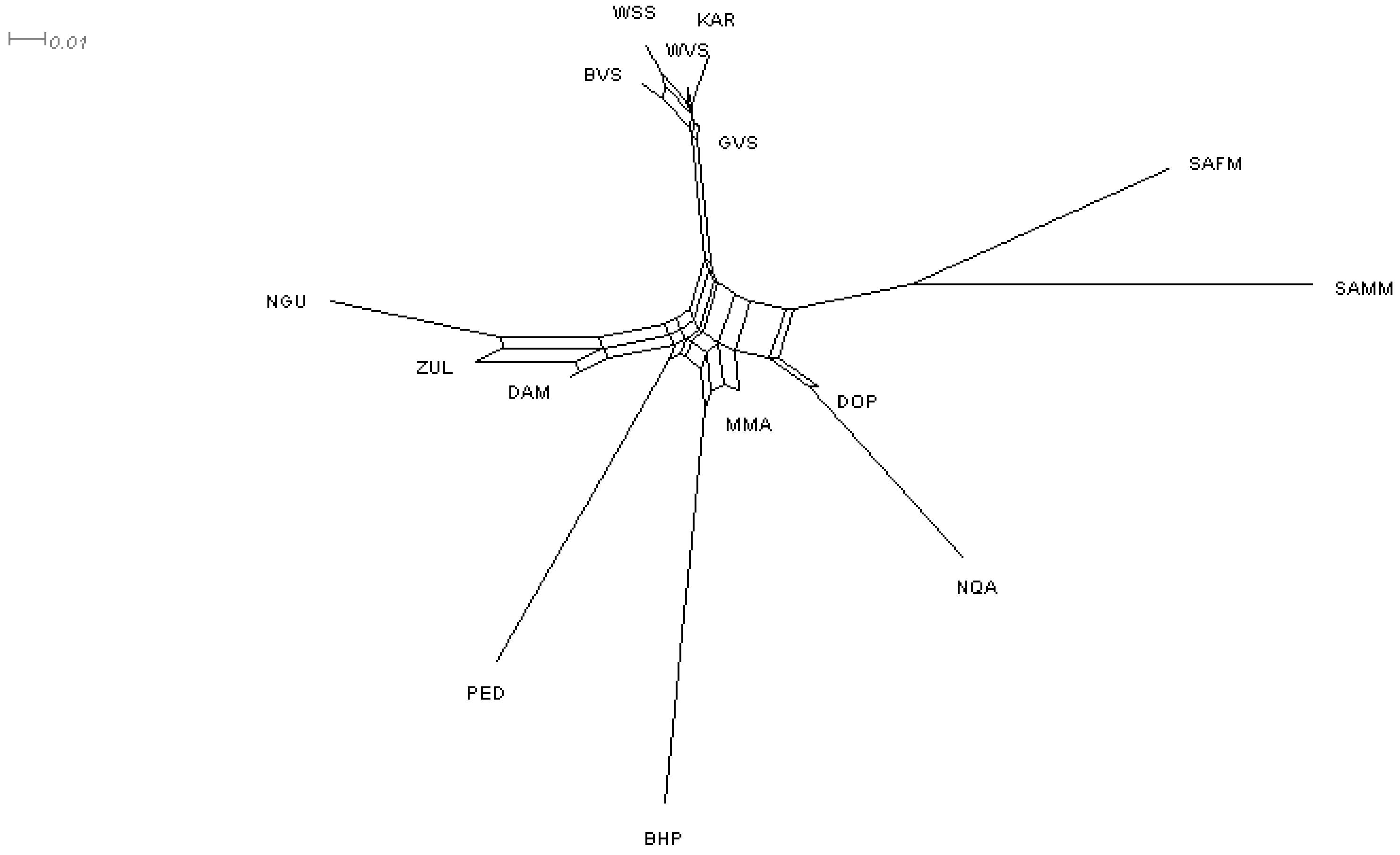

Supplementary Figure 5: Box plots of "genetic integrity level", "AV index", "IBD number" and "IBD length" for 1) goats and 2) sheep, considering "admixed" and "slightly admixed" individuals.

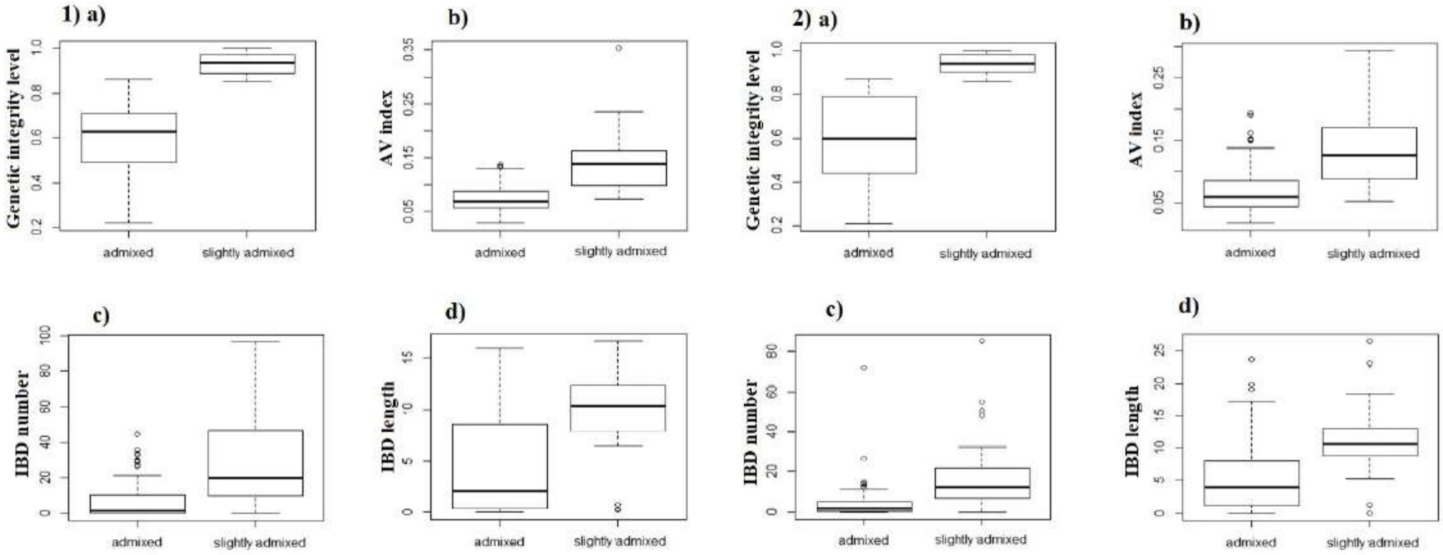

Supplementary Table 1: Details of the goat breeds used.

Breed name	Breed Code	Number of ind. (number retained)	Admixture status	Genetic integrity level	AV index	IBD number	IBD Length (Mb)	country	Region
Barbari	BAB	17	slightly admixed	0.99	0.162	70.51	11.82	Pakistan	Asia
Bari	BRI	35 (30)	admixed	0.79	0.112	29.55	12	Pakistan	Asia
Bugituri	BUT	40 (30)	slightly admixed	0.85	0.115	28.84	16.55	Pakistan	Asia
Chappar	CHA	10	admixed	0.22	0.065	0.08	0.32	Pakistan	Asia
Dera Din Panah	DDP	22	admixed	0.7	0.114	35.7	8.62	Pakistan	Asia
Jattan	JAT	24	admixed	0.61	0.113	29.07	10.36	Pakistan	Asia
Kachan	KAC	24	slightly admixed	0.92	0.156	60.9	15.86	Pakistan	Asia
Kamori	KAM	42 (30)	admixed	0.78	0.134	21.32	15.95	Pakistan	Asia
Koh-e-sulmani	KES	14	admixed	0.25	0.064	1.26	0.63	Pakistan	Asia
Lohri	LOH	25	admixed	0.64	0.108	26.42	11.07	Pakistan	Asia
Local Pothohari	LOP	16	admixed	0.86	0.085	2.84	5.1	Pakistan	Asia
Pahari	PAH	19	admixed	0.74	0.078	5.03	8.4	Pakistan	Asia
Pateri	PAT	37 (30)	admixed	0.57	0.085	7.78	10.21	Pakistan	Asia
Tapri	TAP	24	admixed	0.59	0.085	17.6	9.13	Pakistan	Asia
Teddi	TED	51 (30)	admixed	0.76	0.093	17.86	11.04	Pakistan	Asia
Thari	THA	16	admixed	0.26	0.065	0.46	2.18	Pakistan	Asia
Ankara	ANK	20	admixed	0.65	0.095	2.84	3.27	Turkey	Asia
Kil	KIL	25	admixed	0.57	0.087	1.6	2.55	Turkey	Asia
Kilis	KLS	40 (30)	admixed	0.54	0.080	1.46	2.09	Turkey	Asia
Angora	ANG	26	slightly admixed	0.97	0.147	45.58	9.65	France	Europe
Corse	CRS	30	admixed	0.69	0.063	0.02	0.14	France	Europe
Poitevine	PTV	29	slightly admixed	0.93	0.100	27.49	9.43	France	Europe
Saanen	SAA	56 (30)	admixed	0.81	0.077	14	10.5	France	Europe
Fosses	FSS	26	admixed	0.61	0.082	12.85	8.93	France	Europe
Pyrenean	PYR	27	admixed	0.59	0.089	10.15	9.22	France	Europe
Provencale	PVC	18	admixed	0.78	0.083	14.6	10.77	France	Europe

Rossa Mediterranea	RME	45 (30)	admixed	0.81	0.076	6.58	8.8	Italy	Europe
Orobica	ORO	24	slightly admixed	0.91	0.117	20.3	8.28	Italy	Europe
Argentata	ARG	25	admixed	0.38	0.053	0.1	0.51	Italy	Europe
Aspromontana	ASP	24	admixed	0.37	0.060	2.26	3.44	Italy	Europe
Bionda dell'Adamello	BIO	24	admixed	0.65	0.064	0.67	2.28	Italy	Europe
Ciociara Grigia	CCG	19	admixed	0.39	0.061	0.63	1.05	Italy	Europe
Di Teramo	DIT	24	slightly admixed	0.86	0.118	57.47	13.72	Italy	Europe
Garganica	GAR	20	admixed	0.68	0.098	17.36	9.63	Italy	Europe
Maltese sarda	MLS	15	admixed	0.63	0.108	44.44	10.33	Italy	Europe
Saanen	SAAi	24	admixed	0.67	0.068	11.47	9.94	Italy	Europe
Sarda	SAR	39 (30)	admixed	0.5	0.062	0.13	0.79	Italy	Europe
Valdostana	VAL	24	slightly admixed	0.87	0.093	11.85	9.52	Italy	Europe
Valpassiria	VSS	24	admixed	0.8	0.064	0.48	1.73	Italy	Europe
Girgentana	GGT	30	admixed	0.67	0.108	33.5	9.6	Italy	Europe
Jonica	JON	16	slightly admixed	0.91	0.093	9.88	8.74	Italy	Europe
Maltese	MLT	16	admixed	0.66	0.099	29.83	9.02	Italy	Europe
Nicastrese	NIC	25	admixed	0.46	0.062	1.98	2.36	Italy	Europe
Blanca de Rasquera	RAS	20	admixed	0.63	0.089	7.76	7.5	Spain	Europe
Mallorquina	MAL	20	admixed	0.6	0.102	12.58	7.05	Spain	Europe
Malaguena	MLG	42 (30)	admixed	0.65	0.066	0.49	1.96	Spain	Europe
Murciano-Granadina	MUG	20	admixed	0.7	0.081	3.41	4.92	Spain	Europe
Bermeya	BEY	24	admixed	0.81	0.075	0.3	0.89	Spain	Europe
Alpine suisse	ALP	67 (30)	slightly admixed	0.9	0.090	12.18	11.62	Switzerland	Europe
Boer	BOE	190 (30)	slightly admixed	0.96	0.168	2.54	11.79	Switzerland	Europe
Saanen	SAAs	47 (30)	slightly admixed	0.86	0.099	15.36	10.62	Switzerland	Europe
Traditional Arran	ARR	10	slightly admixed	1	0.235	85.15	10.09	Ireland	North Europe
Bilberry	BLB	10	slightly admixed	0.95	0.216	85.51	13.92	Ireland	North Europe
Icelandic Goat	ICL	13	slightly admixed	1	0.353	96.44	14.4	Iceland	North Europe
Landrace Goat	LNRDK	120 (30)	slightly admixed	0.94	0.159	5.97	10.54	Denmark	North Europe
Landrace Goat	LNRFI	20	slightly admixed	0.98	0.160	3.36	6.52	Finland	North Europe
Landrace Goat	LNRNL	15	slightly admixed	0.99	0.212	62.32	11.08	Netherlands	North Europe

Old Irish Goat	OIG	20	slightly admixed	0.94	0.174	46.84	12.27	Ireland	North Europe
Old Irish goat cross	OIGx	10	admixed	0.45	0.137	9.82	2.15	Ireland	North Europe
Djallonke	DJA	12	admixed	0.81	0.049	0.09	0.16	Burkina Faso	Africa
Sahel	SAH	15	admixed	0.64	0.034	0.04	0.19	Burkina Faso	Africa
Burundi	BUR	72 (30)	slightly admixed	0.91	0.081	0.04	0.23	Burundi	Africa
Cameroon Goat	CAM	40 (30)	admixed	0.67	0.044	0.07	0.29	Cameroon	Africa
West African Dwarf	WAD	34	admixed	0.84	0.058	0.2	0.29	Cameroon	Africa
Barki	BRK	153 (30)	admixed	0.4	0.039	0.16	0.45	Egypt	Africa
Nubian	NBN	84 (30)	slightly admixed	0.86	0.073	11.66	12.37	Egypt	Africa
Oasis	OSS	72 (30)	admixed	0.65	0.050	0.29	0.8	Egypt	Africa
Saidi	SID	60 (30)	admixed	0.6	0.042	0.03	0.04	Egypt	Africa
Gumez	GUM	41 (30)	admixed	0.7	0.069	0.01	0.01	Ethiopia	Africa
Keffa	KEF	49 (30)	admixed	0.83	0.073	0.24	0.86	Ethiopia	Africa
Woyito Guji	WYG	46 (30)	admixed	0.51	0.061	0.05	0.17	Ethiopia	Africa
Abergelle	ABR	53 (30)	slightly admixed	0.9	0.076	0.07	0.34	Ethiopia	Africa
Galla	GAL	23	admixed	0.74	0.057	0.01	0.03	Kenya	Africa
SEA cross	SEAx	30 (30)	admixed	0.58	0.054	0.01	0.05	Kenya	Africa
Diana	DIA	17	slightly admixed	0.85	0.124	10.76	7.6	Madagascar	Africa
Menabe	MEN	21	slightly admixed	0.96	0.163	9.45	7.49	Madagascar	Africa
Sofia	SOF	24	slightly admixed	1	0.154	20	7.86	Madagascar	Africa
Sud Ouest	SOU	10	admixed	0.81	0.132	5.93	6.42	Madagascar	Africa
Balaka-Ulongwe	BAW	12	admixed	0.84	0.083	0.62	1.65	Malawi	Africa
Dedza	DZD	15	slightly admixed	0.96	0.089	0.97	0.77	Malawi	Africa
Thyolo	THY	9	admixed	0.77	0.087	0	0	Malawi	Africa
Guera	GUE	25	admixed	0.71	0.064	13.83	11.83	Mali	Africa
Maure	MAU	14	admixed	0.63	0.038	0.19	1.02	Mali	Africa
Naine	NAI	17	admixed	0.47	0.054	1.59	3.49	Mali	Africa
Peulh	PEU	25	admixed	0.41	0.036	0.03	0.16	Mali	Africa
Soudanaise	SDN	25	admixed	0.29	0.032	0.11	0.39	Mali	Africa
Targui	TAR	22	admixed	0.55	0.034	0.68	0.48	Mali	Africa
Moroccan goat	MOR	10	admixed	0.26	0.033	0	0	Morocco	Africa

Landin	LND	33 (30)	admixed	0.81	0.103	1.4	2.19	Mozambique	Africa
Red Sokoto	RSK	21	admixed	0.3	0.028	0	0	Nigeria	Africa
Sahel	SHL	21	admixed	0.37	0.028	0	0	Nigeria	Africa
West African Dwarf	WADn	21	admixed	0.81	0.053	0.02	0.11	Nigeria	Africa
Angora	ANG	48 (30)	slightly admixed	0.97	0.159	44.81	10.19	South-Africa	Africa
Gogo	GOG	13	admixed	0.57	0.058	0.32	0.63	Tanzania	Africa
Maasai	MAA	20	admixed	0.65	0.059	0.01	0.02	Tanzania	Africa
Malya	MLY	12	admixed	0.77	0.105	32.93	11.24	Tanzania	Africa
Norwegian	NRW	18	slightly admixed	0.89	0.139	14.46	10.69	Tanzania	Africa
Pare White	PRW	22	admixed	0.67	0.075	2.61	5.14	Tanzania	Africa
Sonjo	SNJ	22	admixed	0.46	0.067	0.32	0.85	Tanzania	Africa
Toggenburg	TOG	23	admixed	0.49	0.130	27.26	7.6	Tanzania	Africa
Saanen	SAA	21	slightly admixed	0.86	0.120	29.65	6.81	Tanzania	Africa
Tunisian	TUN	23	admixed	0.38	0.036	0.37	0.63	Tunisia	Africa
Karamonja	KAR	20	admixed	0.43	0.055	0.03	0.05	Uganda	Africa
Mubende	MUB	23	admixed	0.5	0.072	0.9	1.25	Uganda	Africa
Nganda	NGD	11	admixed	0.62	0.064	0.14	0.92	Uganda	Africa
Small East Africa	SEA	15	admixed	0.23	0.048	2.35	3.02	Uganda	Africa
Sebei	SEB	24	admixed	0.6	0.061	0.02	0.17	Uganda	Africa
Boer	BOE	34 (30)	slightly admixed	0.97	0.138	34.19	12.59	Zimbabwe	Africa
Mashona	MSH	23	admixed	0.4	0.081	1.41	0.97	Zimbabwe	Africa
Matebele	MTB	26	admixed	0.52	0.075	3.96	3.88	Zimbabwe	Africa
Matabele cross	MTBx	24	admixed	0.56	0.100	17.81	8.5	Zimbabwe	Africa

Name, code, country of sampling, number of individuals considered, admixture status, “genetic integrity level”, average number and size of IBD segments shared between individuals of the breed. Ind. = individual.

Supplementary Table 2: Details of the sheep breeds used.

Breed name	Breed code	Number of ind. (number retained)	Admixture status	Genetic integrity level	AV index	IBD number	IBD length (Mb)	Country	Region
Adile	ADI	11	admixed	0.6	0.053	0.00	0.00	Ethiopia	Africa
Adane	AKD	12	admixed	0.34	0.060	3.42	3.90	Ethiopia	Africa
Arabo	AKR	10	admixed	0.58	0.059	0.58	1.67	Ethiopia	Africa
ArsiBale	ARB	8	admixed	0.81	0.061	0.00	0.00	Ethiopia	Africa
Black Headed Somali	BHS	12	slightly admixed	0.97	0.062	0.00	0.00	Ethiopia	Africa
Bonga	BON	15	slightly admixed	0.99	0.081	0.00	0.00	Ethiopia	Africa
Doyogena	DOY	15	admixed	0.85	0.051	0.01	0.06	Ethiopia	Africa
Farta	FRT	41 (30)	admixed	0.5	0.041	0.02	0.12	Ethiopia	Africa
Gesses	GGD	10	admixed	0.56	0.065	0.56	1.59	Ethiopia	Africa
Horro	HOR	44 (30)	admixed	0.57	0.043	0.01	0.10	Ethiopia	Africa
Hammari	HRI	18	admixed	0.64	0.064	0.09	0.57	Ethiopia	Africa
Kefis	KEF	14	admixed	0.74	0.067	0.31	0.86	Ethiopia	Africa
Kido	KID	10	admixed	0.41	0.067	2.13	3.04	Ethiopia	Africa
Kabashi	KSH	18	admixed	0.44	0.067	0.32	0.71	Ethiopia	Africa
Loya	LOY	15	admixed	0.62	0.077	2.54	2.18	Ethiopia	Africa
Menz	MNZ	27	admixed	0.84	0.060	0.04	0.26	Ethiopia	Africa
Shubi Gemo	SHG	15	admixed	0.42	0.053	1.18	1.94	Ethiopia	Africa
Wollo	WOL	39 (30)	admixed	0.47	0.044	0.02	0.17	Ethiopia	Africa
Washera	WSH	59 (30)	admixed	0.37	0.043	0.22	0.95	Ethiopia	Africa
Blackhead Persian	BHP	8	slightly admixed	0.98	0.208	54.64	11.44	South Africa	Africa
Damara	DAM	30	admixed	0.68	0.119	10.89	8.50	South Africa	Africa
Dorper	DOP	17	admixed	0.61	0.114	14.03	5.28	South Africa	Africa
Karakul	KAR	41 (30)	admixed	0.53	0.152	5.31	12.09	South Africa	Africa
Meatmaster	MMA	19	admixed	0.45	0.107	13.18	10.38	South Africa	Africa
Nguni	NGU	30	slightly admixed	0.89	0.175	28.89	16.12	South Africa	Africa
Pedi	PED	28	slightly admixed	0.93	0.190	32.69	26.46	South Africa	Africa
SA Merino	SAFM	10	slightly admixed	1	0.204	31.73	9.70	South Africa	Africa
SA Mutton Merino	SAMM	10	slightly admixed	0.98	0.234	47.96	10.19	South Africa	Africa
Swakara1	BVS	16	admixed	0.62	0.124	11.16	11.31	South Africa	Africa

Swakara2	GVS	22	admixed	0.35	0.108	4.52	11.50	South Africa	Africa
Swakara3	WSS	17	admixed	0.55	0.130	10.55	12.15	South Africa	Africa
Swakara4	WVS	41 (30)	admixed	0.43	0.116	7.24	10.80	South Africa	Africa
Zulu	ZUL	28	admixed	0.68	0.137	12.26	19.05	South Africa	Africa
Bangladeshi Garole	BGA	24	admixed	0.85	0.112	3.55	4.21	Pacific Asia	Asia
Bangladeshi	BGE	24	slightly admixed	0.93	0.106	1.30	1.36	Pacific Asia	Asia
Changthangi	CHA	24	admixed	0.72	0.096	1.50	1.52	Pacific Asia	Asia
Indian Garole	GAR	26	slightly admixed	0.9	0.128	7.41	5.28	Pacific Asia	Asia
Garut	GUR	22	slightly admixed	0.96	0.125	8.30	6.75	Pacific Asia	Asia
Deccani	IDC	24	admixed	0.82	0.098	0.02	0.13	Pacific Asia	Asia
Sumatra	SUM	24	slightly admixed	0.91	0.126	6.96	5.53	Pacific Asia	Asia
Tibetan	TIB	37 (30)	admixed	0.87	0.116	0.78	1.17	Pacific Asia	Asia
Baerchuke	BRK	20	admixed	0.32	0.045	1.91	1.79	China	Asia
Bashbay	BSB	20	admixed	0.3	0.036	0.25	0.46	China	Asia
Celeiblack	CLS	19	admixed	0.77	0.067	7.39	9.76	China	Asia
Diqing	DQS	20	slightly admixed	0.9	0.089	21.63	12.73	China	Asia
Guide Black Fur	GDS	20	admixed	0.75	0.055	1.12	3.99	China	Asia
Guangling fat tail	GLS	20	admixed	0.52	0.062	1.77	6.38	China	Asia
Hulun Buir	HLS	20	admixed	0.31	0.043	0.12	0.89	China	Asia
Hetian	HTS	24	admixed	0.45	0.054	2.97	4.76	China	Asia
Hanzhong	HZS	20	slightly admixed	0.9	0.084	6.53	11.87	China	Asia
Jingzhong	JZS	20	admixed	0.29	0.042	0.67	1.45	China	Asia
Kirghiz	KIR	19	admixed	0.43	0.041	0.09	0.66	China	Asia
Lanzhou Large tailed	LAN	20	admixed	0.3	0.052	0.43	1.96	China	Asia
Lop	LOP	17	admixed	0.37	0.044	0.30	1.79	China	Asia
Luzhong Mountain	LZS	20	admixed	0.36	0.053	2.63	3.73	China	Asia
Minxian Black Fur	MXS	20	admixed	0.42	0.051	3.99	4.78	China	Asia
Ninglang Black	NLS	20	admixed	0.59	0.069	1.01	3.13	China	Asia
Sunite	SNS	22	admixed	0.47	0.044	0.66	6.27	China	Asia
Shiping Gray	SPS	20	slightly admixed	1	0.143	29.82	10.63	China	Asia
Tan	TAN	20	admixed	0.5	0.046	1.52	4.52	China	Asia
Tengchong	TCS	20	admixed	0.81	0.076	4.69	10.71	China	Asia
TaihangFur	THQ	18	admixed	0.65	0.056	0.63	3.64	China	Asia
Turfan Black	TLF	18	admixed	0.21	0.036	0.20	1.26	China	Asia
Tong	TON	20	admixed	0.68	0.062	2.96	6.37	China	Asia

Tashkurgan	TSK	20	admixed	0.48	0.058	1.35	7.39	China	Asia
Lanping Blackbone	WGS	20	slightly admixed	0.89	0.081	1.47	6.43	China	Asia
Weining	WNS	20	slightly admixed	0.89	0.092	26.60	6.12	China	Asia
Wuranke	WRS	20	admixed	0.42	0.040	0.08	0.33	China	Asia
Ujimqin	WZS	20	admixed	0.41	0.043	0.08	0.55	China	Asia
Yecheng	YEC	20	admixed	0.27	0.039	0.13	0.81	China	Asia
Yuxi Fat tailed	YXZ	18	slightly admixed	0.92	0.073	14.59	8.25	China	Asia
Tibetan Qamdo	ZCD	20	admixed	0.58	0.063	3.18	2.75	China	Asia
Tibetan Nyingchi	ZLZ	20	admixed	0.71	0.097	26.73	8.17	China	Asia
Tibetan Nagqu	ZNQ	20	admixed	0.53	0.059	2.15	3.26	China	Asia
Tibetan Bainang	ZRK	20	admixed	0.81	0.082	8.04	8.81	China	Asia
Zhaotong	ZTS	20	admixed	0.73	0.149	72.13	19.87	China	Asia
Cyprus Fat Tail	CFT	30	slightly admixed	0.94	0.127	13.97	8.87	South West Asia	Asia
Karakas	KRS	18	admixed	0.63	0.080	4.27	5.61	South West Asia	Asia
Moghani	MOG	34 (30)	admixed	0.84	0.062	0.25	1.24	South West Asia	Asia
Norduz	NDZ	20	admixed	0.85	0.091	6.65	8.55	South West Asia	Asia
Qezel	QEZ	35 (30)	admixed	0.59	0.056	0.08	0.10	South West Asia	Asia
Sakiz	SKZ	22	slightly admixed	0.96	0.147	15.72	6.72	South West Asia	Asia
Afshari	AFS	37 (30)	admixed	0.79	0.079	4.18	7.01	South West Asia	Europe
Bundner Oberlander Sheep	BOS	21	slightly admixed	0.86	0.130	14.88	15.58	Central Europe	Europe
East-Friesian Brown	EFB	39 (30)	admixed	0.56	0.191	9.20	23.59	Central Europe	Europe
East-Friesian White	EFW	9	slightly admixed	0.99	0.171	14.61	12.11	Central Europe	Europe
Engadine Red Sheep	ERS	21	slightly admixed	0.89	0.109	1.92	6.66	Central Europe	Europe
Swiss Black-Brown Mountain Sheep	SBS	23	admixed	0.81	0.126	11.07	11.33	Central Europe	Europe
Swiss Mirror Sheep	SMS	20	admixed	0.8	0.126	14.78	14.07	Central Europe	Europe
Swiss White Alpine Sheep	SWA	20	slightly admixed	0.88	0.127	6.21	10.69	Central Europe	Europe
Valais Blacknose Sheep	VBS	19	slightly admixed	0.92	0.158	12.08	10.85	Central Europe	Europe
Valais Red Sheep	VRS	21	slightly admixed	0.97	0.163	21.63	15.49	Central Europe	Europe
Black-Headed Mutton	BHM	24	admixed	0.59	0.151	12.05	17.26	Central Europe	Europe
Berrichon du Cher	BER	19	slightly admixed	0.99	0.164	32.53	13.00	France	Europe
Blanche du Massif Central	BMC	20	admixed	0.85	0.080	1.06	6.12	France	Europe
Causses du Lot	CDL	20	slightly admixed	0.97	0.116	9.04	10.12	France	Europe
Charollais	CHA	24	slightly admixed	0.96	0.111	8.10	10.53	France	Europe
Charmoise	CHR	23	slightly admixed	0.98	0.143	19.03	10.65	France	Europe
Île-de-France	IDF	23	slightly admixed	0.97	0.120	12.16	11.05	France	Europe

Lacaune (milk)	LAC	36 (30)	slightly admixed	0.91	0.087	4.28	10.23	France	Europe
Lacaune (meat)	LAM	34 (30)	admixed	0.78	0.079	2.71	8.00	France	Europe
Limousine	LIM	18	slightly admixed	0.98	0.111	9.00	10.53	France	Europe
Mérinos d'Arles	MER	18	admixed	0.85	0.085	0.77	4.22	France	Europe
Mourerous	MOU	16	admixed	0.42	0.081	1.48	6.47	France	Europe
Manech tête rousse	MTR	25	slightly admixed	0.97	0.103	5.47	12.36	France	Europe
Noire du Velay	NVE	19	slightly admixed	0.93	0.086	2.02	7.82	France	Europe
Ouessant	OUE	18	slightly admixed	0.98	0.254	85.16	13.07	France	Europe
Préalpes du Sud	PAS	17	admixed	0.85	0.082	1.77	6.57	France	Europe
Mérinos de Rambouillet	RAM	27	slightly admixed	1	0.274	50.30	14.25	France	Europe
Rava	RAV	20	slightly admixed	0.9	0.094	4.40	10.43	France	Europe
Romane	RMN	19	admixed	0.55	0.097	5.84	10.83	France	Europe
Romanov	ROM	10	slightly admixed	1	0.143	5.31	8.95	France	Europe
Roussin de la Hague	ROU	21	slightly admixed	0.95	0.126	21.35	11.71	France	Europe
Rouge de l'Ouest	RWE	17	slightly admixed	0.97	0.112	5.99	10.65	France	Europe
Suffolk	SUF	19	slightly admixed	0.99	0.136	12.92	10.20	France	Europe
Tarasconnaise	TAR	15	admixed	0.85	0.086	1.10	4.94	France	Europe
Texel	TEX	24	slightly admixed	0.99	0.136	12.44	10.43	France	Europe
Vendéen	VEN	22	slightly admixed	0.97	0.128	15.04	11.14	France	Europe
Alpagota	ALP	24	slightly admixed	0.86	0.069	6.82	9.61	Italy	Europe
Altamurana	ALT	19	slightly admixed	0.9	0.058	8.30	8.09	Italy	Europe
Appenninica	APP	24	admixed	0.7	0.058	8.40	12.50	Italy	Europe
Bagnolese	BAG	23	admixed	0.26	0.042	1.20	2.08	Italy	Europe
Bergamasca	BERG	22	admixed	0.83	0.051	0.76	3.70	Italy	Europe
Biellese	BIE	21	admixed	0.85	0.052	0.68	3.74	Italy	Europe
Comisana	COM	24	slightly admixed	0.92	0.053	1.43	5.59	Italy	Europe
DelleLanghe	DEL	24	slightly admixed	0.91	0.082	18.21	11.68	Italy	Europe
Fabrianese	FAB	23	slightly admixed	0.87	0.065	11.36	13.55	Italy	Europe
Gentile di Puglia	GEN	24	slightly admixed	0.9	0.070	14.05	15.76	Italy	Europe
Laticauda	LAT	24	admixed	0.71	0.053	1.88	4.74	Italy	Europe
Leccese	LEC	19	admixed	0.53	0.050	4.92	4.21	Italy	Europe
Massese	MAS	24	admixed	0.81	0.069	7.65	9.76	Italy	Europe
Pinzirita	PIN	24	admixed	0.55	0.044	2.00	1.31	Italy	Europe
Sambucana	SAM	22	admixed	0.82	0.057	6.12	10.18	Italy	Europe
Sardinian White	SAW	24	slightly admixed	0.86	0.069	5.73	9.47	Italy	Europe

Sopravissana	SOP	24	admixed	0.65	0.046	2.22	2.24	Italy	Europe
Valle del Belice	VBE	24	slightly admixed	0.89	0.072	16.34	12.56	Italy	Europe
Boreray	BOR	17	slightly admixed	0.9	0.288	32.78	17.31	Northern Europe	Europe
Border Leicester	BRL	48 (30)	slightly admixed	0.98	0.248	6.69	13.32	Northern Europe	Europe
Dorset Horn	DSH	21	slightly admixed	0.92	0.271	23.48	23.01	Northern Europe	Europe
Galway	GAL	40 (30)	slightly admixed	0.92	0.184	9.85	14.75	Northern Europe	Europe
GermanTexel	GTX	42 (30)	admixed	0.76	0.162	4.93	11.66	Northern Europe	Europe
Irish Suffolk	ISF	46 (30)	slightly admixed	0.97	0.214	10.39	13.03	Northern Europe	Europe
Scottish Black face	SBF	40 (30)	slightly admixed	0.91	0.170	5.69	11.17	Northern Europe	Europe
Soay	SOA	55 (30)	slightly admixed	1	0.293	9.53	13.95	Northern Europe	Europe
Scottish Texel	STX	42 (30)	admixed	0.81	0.194	6.47	23.57	Northern Europe	Europe
Wiltshire	WIL	23	slightly admixed	0.97	0.292	29.42	18.36	Northern Europe	Europe
Castellana	CAS	21	admixed	0.79	0.030	1.90	5.62	Spain	Europe
Latxa	LTX	24	admixed	0.6	0.030	2.13	3.02	Spain	Europe
Merino Extremadura	MEE	13	admixed	0.53	0.028	1.62	4.86	Spain	Europe
Merino	MER	18	admixed	0.81	0.033	1.23	4.42	Spain	Europe
Ojalada	OJA	23	admixed	0.76	0.028	1.76	6.04	Spain	Europe
Rasa Aragonesa	RAA	20	admixed	0.23	0.017	0.02	0.09	Spain	Europe
Ripollesa	RIP	21	admixed	0.8	0.027	1.62	3.51	Spain	Europe
Sasi Ardi	SAA	24	admixed	0.67	0.029	0.37	1.57	Spain	Europe
Segurena	SEG	12	admixed	0.24	0.022	0.18	0.89	Spain	Europe
Xisqueta	XIS	24	admixed	0.27	0.020	0.44	0.81	Spain	Europe

Name, code, country of sampling, number of individuals considered, admixture status, “genetic integrity level”, average number and size of IBD segments shared between individuals of the breed. Ind. = individual.

Supplementary Table 3: Inbreeding index for sheep breeds.

Breed code	Breed name	Region	Lower limit confidence Interval 95%	Upper limit confidence Interval 95%	mean F_{IS}
IDC	Deccani	West Asia	0.0037	0.0071	0.0054
GUR	Garut	West Asia	0.0032	0.0079	0.0055
GAR	Indian Garole	West Asia	0.0263	0.0295	0.0279
SUM	Sumatra	West Asia	0.0435	0.0483	0.0459
TIB	Tibetan	West Asia	0.0652	0.0689	0.0670
CHA	Changthangi	West Asia	0.0704	0.0742	0.0723
BGA	Bangladeshi Garole	West Asia	0.0722	0.0763	0.0742
BGE	Bangladeshi	West Asia	0.1446	0.1493	0.1469
DSH	Dorset Horn	Central North Europe	-0.0664	-0.0619	-0.0641
STX	Scottish Texel	Central North Europe	-0.043	-0.0387	-0.0408
BOS	Bundner Oberlander Sheep	Central North Europe	-0.0273	-0.023	-0.0251
VRS	Valais Red Sheep	Central North Europe	-0.0223	-0.0181	-0.0202
SOA	Soay	Central North Europe	-0.003	0.0001	-0.0014
SMS	Swiss Mirror Sheep	Central North Europe	-0.0031	0.0011	-0.0010
SBS	Swiss Black-Brown Mountain Sheep	Central North Europe	-0.0016	0.0025	0.0004
GAL	Galway	Central North Europe	0.0014	0.0047	0.0030
EFW	East-Friesian White	Central North Europe	-0.0006	0.007	0.0032
ERS	Engadine Red Sheep	Central North Europe	0.0033	0.0077	0.0055
SWA	Swiss White Alpine Sheep	Central North Europe	0.0038	0.0087	0.0062
WIL	Wiltshire	Central North Europe	0.0045	0.0091	0.0068
BOR	Boreray	Central North Europe	0.0124	0.0178	0.0151
EFB	East-Friesian Brown	Central North Europe	0.0269	0.0307	0.0288
GTX	GermanTexel	Central North Europe	0.0272	0.031	0.0291
BRL	Border Leicester	Central North Europe	0.0278	0.0323	0.0300
SBF	Scottish Black face	Central North Europe	0.0281	0.0329	0.0305
BHM	Black-Headed Mutton	Central North Europe	0.0386	0.0427	0.0406
VBS	Valais Blacknose Sheep	Central North Europe	0.0522	0.0564	0.0543
ISF	Irish Suffolk	Central North Europe	0.0559	0.0601	0.0580

ZTS	Zhaotong	Chinese	-0.0512	-0.0434	-0.0473
HZS	Hanzhong	Chinese	-0.0416	-0.0366	-0.0391
GLS	Guangling fat tail	Chinese	-0.0372	-0.0321	-0.0346
THQ	TaihangFur	Chinese	-0.0322	-0.0255	-0.0288
LAN	Lanzhou Large tailed	Chinese	-0.0307	-0.0253	-0.0280
TSK	Tashkurgan	Chinese	-0.0192	-0.0134	-0.0163
GDS	Guide Black Fur	Chinese	-0.0171	-0.0107	-0.0139
ZNQ	Tibetan Nagqu	Chinese	-0.0125	-0.0061	-0.0093
TON	Tong	Chinese	-0.0058	0,000	-0.0029
HLS	Hulun Buir	Chinese	-0.0053	0.0016	-0.0018
HTS	Hetian	Chinese	-0.0045	0.0008	-0.0018
SNS	Sunite	Chinese	-0.0038	0.0023	-0.0007
DQS	Diqing	Chinese	-0.0022	0.0042	0.0010
NLS	Ninglang Black	Chinese	-0.0017	0.0041	0.0012
WZS	Ujimqin	Chinese	-0.0005	0.0058	0.0026
TCS	Tengchong	Chinese	0.0002	0.006	0.0031
ZRK	Tibetan Bainang	Chinese	0.0016	0.0084	0.0050
CLS	Celeiblack	Chinese	0.0121	0.0179	0.0150
YEC	Yecheng	Chinese	0.0122	0.0184	0.0153
WRS	Wuranke	Chinese	0.0137	0.0192	0.0164
WGS	Lanping Blackbone	Chinese	0.0134	0.0196	0.0165
BRK	Baerchuke	Chinese	0.0173	0.0237	0.0205
KIR	Kirghiz	Chinese	0.0183	0.0248	0.0215
BSB	Bashbay	Chinese	0.0218	0.0277	0.0247
ZLZ	Tibetan Nyingchi	Chinese	0.026	0.0328	0.0294
TAN	Tan	Chinese	0.0308	0.0364	0.0336
LOP	Lop	Chinese	0.0313	0.0381	0.0347
TLF	Turfan Black	Chinese	0.032	0.0385	0.0352
LZS	Luzhong Mountain	Chinese	0.0385	0.0448	0.0416
MXS	Minxian Black Fur	Chinese	0.0471	0.0526	0.0498
JZS	Jingzhong	Chinese	0.0494	0.0554	0.0524
SPS	Shiping Gray	Chinese	0.0507	0.0573	0.0540
ZCD	Tibetan Qamdo	Chinese	0.0627	0.0689	0.0658
YXZ	Yuxi Fat tailed	Chinese	0.0664	0.0743	0.0703
WNS	Weining	Chinese	0.1054	0.1133	0.1093
GGD	Gesses	Ethiopia	-0.0388	-0.0291	-0.0339
KID	Kido	Ethiopia	-0.0227	-0.0138	-0.0182
AKR	Arabo	Ethiopia	-0.0084	0.0025	-0.0029
KEF	Kefis	Ethiopia	-0.0051	0.0038	-0.0006
SHG	Shubi Gemo	Ethiopia	-0.0007	0.0072	0.0032
LOY	Loya	Ethiopia	0.0009	0.0105	0.0057
BON	Bonga	Ethiopia	0.004	0.0134	0.0087
FRT	Farta	Ethiopia	0.007	0.0123	0.0096
WSH	Washera	Ethiopia	0.0074	0.0119	0.0096
DOY	Doyogena	Ethiopia	0.0084	0.0167	0.0125
HRI	Hammari	Ethiopia	0.0151	0.0235	0.0193
WOL	Wollo	Ethiopia	0.0204	0.025	0.0227
MNZ	Menz	Ethiopia	0.0204	0.0284	0.0244
AKD	Adane	Ethiopia	0.0213	0.0308	0.0260
HOR	Horro	Ethiopia	0.0241	0.0289	0.0265
BHS	Black Headed Somali	Ethiopia	0.0278	0.0357	0.0317

ARB	ArsiBale	Ethiopia	0.0309	0.0428	0.0368
ADI	Adile	Ethiopia	0.0378	0.0468	0.0423
KSH	Kabashi	Ethiopia	0.0401	0.0504	0.0452
RAM	Mérinos de Rambouillet	South Europe	-0.0302	-0.0229	-0.0265
FAB	Fabrianese	South Europe	-0.0273	-0.0212	-0.0242
GEN	Gentile di Puglia	South Europe	-0.018	-0.0125	-0.0152
BER	Berrichon du Cher	South Europe	-0.0174	-0.0095	-0.0134
COM	Comisana	South Europe	-0.0135	-0.0066	-0.0100
APP	Appenninica	South Europe	-0.0133	-0.0065	-0.0099
CAS	Castellana	South Europe	-0.0101	-0.0038	-0.0069
CDL	Causses du Lot	South Europe	-0.0093	-0.0015	-0.0054
SAW	Sardinian White	South Europe	-0.0069	-0.0011	-0.0040
MER	Mérinos d'Arles	South Europe	-0.0078	-0.0001	-0.0039
RWE	Rouge de l'Ouest	South Europe	-0.0075	0.0011	-0.0032
MTR	Manech tête rousse	South Europe	-0.005	0.0005	-0.0022
RAV	Rava	South Europe	-0.0037	0.0023	-0.0007
CHR	Charmoise	South Europe	-0.0037	0.0032	-0.00025
ROU	Roussin de la Hague	South Europe	-0.0039	0.0037	-0.0001
SUF	Suffolk	South Europe	-0.0032	0.0033	0.0001
LIM	Limousine	South Europe	-0.0031	0.0038	0.0003
NVE	Noire du Velay	South Europe	-0.0034	0.0041	0.0003
RMN	Romane	South Europe	-0.0033	0.0048	0.0007
OJA	Ojalada	South Europe	-0.002	0.0042	0.0011
IDF	Île-de-France	South Europe	-0.0013	0.0056	0.0021
PAS	Préalpes du Sud	South Europe	-0.0016	0.0061	0.0022
MAS	Massese	South Europe	-0.0004	0.0075	0.0035
VEN	Vendéen	South Europe	0.0004	0.0073	0.0038
CHA	Charollais	South Europe	0.0018	0.0081	0.0049
TEX	Texel	South Europe	0.0023	0.0085	0.0054
MEE	Merino Extremadura	South Europe	0.0011	0.0099	0.0055
RAA	Rasa Aragonesa	South Europe	0.002	0.0091	0.0055
MOU	Mourerous	South Europe	0.0026	0.0099	0.0062
BMC	Blanche du Massif Central	South Europe	0.0035	0.0116	0.0075
XIS	Xisqueta	South Europe	0.0076	0.013	0.0103
SEG	Segurena	South Europe	0.0073	0.0144	0.0108
SAM	Sambucana	South Europe	0.0087	0.0154	0.0120
LAM	Lacaune (meat)	South Europe	0.0115	0.0171	0.0143
LAC	Lacaune (milk)	South Europe	0.0115	0.0172	0.0143
TAR	Tarasconnaise	South Europe	0.0117	0.0197	0.0157
DEL	DelleLanghe	South Europe	0.0124	0.0192	0.0158
ALT	Altamurana	South Europe	0.0125	0.0202	0.0163
ALP	Alpagota	South Europe	0.0142	0.0211	0.0176
BER	Berrichon du Cher	South Europe	0.0143	0.0211	0.0177
ROM	Romanov	South Europe	0.016	0.0262	0.0211
LAT	Laticauda	South Europe	0.0191	0.0265	0.0228
SAA	Sasi Ardi	South Europe	0.021	0.0267	0.0238
RIP	Ripollesa	South Europe	0.0258	0.0317	0.0287
SOP	Sopravissana	South Europe	0.0263	0.0326	0.0294
BIE	Biellese	South Europe	0.0265	0.0329	0.0297
MER	Mérinos d'Arles	South Europe	0.0311	0.0371	0.0341
BAG	Bagnolese	South Europe	0.0321	0.0387	0.0354

LTX	Latxa	South Europe	0.0353	0.0414	0.0383
VBE	Valle del Belice	South Europe	0.0352	0.0423	0.0387
PIN	Pinzirita	South Europe	0.0385	0.044	0.0412
LEC	Leccese	South Europe	0.0622	0.0697	0.0659
OUE	Ouessant	South Europe	0.1729	0.1816	0.1772
PED	Pedi	South Africa	-0.0945	-0.0887	-0.0916
BHP	Blackhead Persian	South Africa	-0.0632	-0.0481	-0.0556
SAMM	SAMutton Merino	South Africa	-0.0347	-0.0245	-0.0296
ZUL	Zulu	South Africa	-0.0318	-0.0247	-0.0282
MMA	Meatmaster	South Africa	-0.0251	-0.0174	-0.0212
SAFM	SAMerino	South Africa	-0.0127	-0.0033	-0.008
NGU	Nguni	South Africa	0.008	0.0167	0.0123
KAR	Karakul	South Africa	0.0296	0.0362	0.0329
GVS	Swakara2	South Africa	0.0388	0.0463	0.0425
DAM	Damara	South Africa	0.0403	0.0474	0.0438
BVS	Swakara1	South Africa	0.0848	0.0938	0.0893
WSS	Swakara3	South Africa	0.0924	0.1012	0.0968
WVS	Swakara4	South Africa	0.1024	0.109	0.1057
DOP	Dorper	South Africa	0.1215	0.13	0.1257
SKZ	Sakiz	Pacific Asia	-0.0566	-0.0524	-0.0545
NDZ	Norduz	Pacific Asia	-0.0366	-0.0324	-0.0345
CFT	Cyprus Fat Tail	Pacific Asia	-0.033	-0.0295	-0.0312
AFS	Afshari	Pacific Asia	-0.0189	-0.0161	-0.0175
KRS	Karakas	Pacific Asia	-0.0104	-0.005	-0.0077
MOG	Moghani	Pacific Asia	0.0162	0.02	0.0181
QEZ	Qezel	Pacific Asia	0.0337	0.0375	0.0356

Mean F_{IS} by population, 95% confidence interval upper and lower bounds.

Supplementary Table 4: Inbreeding index for goat breeds.

Breed code	Breed name	Region	Lower limit confidence Interval 95%	Upper limit confidence Interval 95%	mean F_{IS}
BOE	Boer	East Africa	-0.0799	-0.0736	-0.07675
MLY	Malya	East Africa	-0.0613	-0.051	-0.05615
MTBx	Matabele cross	East Africa	-0.0506	-0.0423	-0.04645
NRW	Norwegian	East Africa	-0.0223	-0.0129	-0.0176
SNJ	Sonjo	East Africa	-0.0169	-0.0081	-0.0125
MTB	Matebele	East Africa	-0.0136	-0.006	-0.0098
SOU	Sud Ouest	East Africa	-0.0132	0.0005	-0.00635
SEB	Sebei	East Africa	-0.0072	0.0008	-0.0032
GOG	Gogo	East Africa	-0.0027	0.0075	0.0024
ANG	Angora	East Africa	0.0005	0.0085	0.0045
SAA	Saanen	East Africa	0.0005	0.0104	0.00545
BUR	Burundi	East Africa	0.0018	0.0092	0.0055
PRW	Pare White	East Africa	0.0022	0.0102	0.0062
GAL	Galla	East Africa	0.0033	0.0112	0.00725
MAA	Maasai	East Africa	0.0043	0.0117	0.008
NGD	Nganda	East Africa	0.0072	0.0169	0.01205
WYG	Woyito Guji	East Africa	0.01	0.0183	0.01415
GUM	Gumez	East Africa	0.0109	0.0188	0.01485
MUB	Mubende	East Africa	0.0127	0.0202	0.01645
SEAX	SEA cross	East Africa	0.0133	0.0201	0.0167
ABR	Abergelle	East Africa	0.0131	0.0217	0.0174
MEN	Menabe	East Africa	0.0133	0.0224	0.01785
KAR	Karamonja	East Africa	0.0148	0.0231	0.01895
TOG	Toggenburg	East Africa	0.0247	0.0323	0.0285
LND	Landin	East Africa	0.0259	0.0347	0.0303
KEF	Keffa	East Africa	0.0273	0.0344	0.03085
BAW	Balaka-Ulongwe	East Africa	0.027	0.0366	0.0318
MSH	Mashona	East Africa	0.039	0.0463	0.04265
SEA	Small East Africa	East Africa	0.0561	0.0617	0.0589
DIA	Diana	East Africa	0.0566	0.066	0.0613
SOF	Sofia	East Africa	0.0631	0.0725	0.0678
DZD	Dedza	East Africa	0.077	0.0885	0.08275
THY	Thyolo	East Africa	0.1007	0.1117	0.1062
GUE	Guera	West Africa	-0.0658	-0.0598	-0.0628
MAU	Maure	West Africa	-0.0214	-0.0119	-0.01665
PEU	Peulh	West Africa	-0.0124	-0.0059	-0.00915
SDN	Soudanaise	West Africa	-0.0001	0.0067	0.0033
NAI	Naine	West Africa	0.0004	0.0086	0.0045
TAR	Targui	West Africa	0.0047	0.0118	0.00825
DJA	Djallonke	West Africa	0.004	0.0138	0.0089
CAM	Cameroon Goat	West Africa	0.0076	0.0147	0.01115
SAH	Sahel	West Africa	0.0106	0.0185	0.01455
TUN	Tunisian	West Africa	0.0129	0.0189	0.0159
SHL	Sahel	West Africa	0.0142	0.0212	0.0177
NBN	Nubian	West Africa	0.0156	0.0234	0.0195
BRK	Barki	West Africa	0.0181	0.0252	0.02165
WAD	West African Dwarf Nigeria	West Africa	0.0222	0.0308	0.0265

WADc	West African Dwarf Cameroon	West Africa	0.0299	0.0355	0.0327
RSK	Red Sokoto	West Africa	0.0337	0.0419	0.0378
OSS	Oasis	West Africa	0.0471	0.055	0.05105
MOR	Moroccan goat	West Africa	0.0648	0.0748	0.0698
SID	Saidi	West Africa	0.072	0.0807	0.07635
BAB	Barbari	Asia	-0.0563	-0.0479	-0.0521
LOH	Lohri	Asia	-0.0308	-0.0244	-0.0276
PAH	Pahari	Asia	-0.0074	-0.0005	-0.00395
LOP	Local Pothohari	Asia	-0.0063	0.0011	-0.0026
KAM	Kamori	Asia	-0.0031	0.0022	-0.00045
KAC	Kachan	Asia	0.0021	0.0085	0.0053
PAT	Pateri	Asia	0.006	0.0109	0.00845
KIL	Kil	Asia	0.0062	0.0117	0.00895
THA	Thari	Asia	0.0064	0.0146	0.0105
CHA	Chappar	Asia	0.011	0.0177	0.01435
KLS	Kilis	Asia	0.0156	0.0201	0.01785
ANK	Ankara	Asia	0.0208	0.0311	0.02595
JAT	Jattan	Asia	0.023	0.0309	0.02695
DDP	Dera Din Panah	Asia	0.0257	0.0323	0.029
BUT	Bugituri	Asia	0.0294	0.0369	0.03315
KES	Koh-e-sulmani	Asia	0.0361	0.0451	0.0406
TAP	Tapri	Asia	0.0398	0.0465	0.04315
TED	Teddi	Asia	0.0468	0.0511	0.04895
BRI	Bari	Asia	0.0479	0.0546	0.05125
JON	Jonica	South Europe	-0.0855	-0.0763	-0.0809
GAR	Garganica	South Europe	-0.0723	-0.0647	-0.0685
DIT	Di Teramo	South Europe	-0.0614	-0.0548	-0.0581
SAR	Sarda	South Europe	-0.0556	-0.0484	-0.052
RME	Rossa Mediterranea	South Europe	-0.0364	-0.0288	-0.0326
SAA	Saanen	South Europe	-0.0287	-0.0213	-0.025
BOE	Boer	South Europe	-0.0223	-0.016	-0.01915
PAL	Palmera	South Europe	-0.0173	-0.0091	-0.0132
SAAFR	Saanen France	South Europe	-0.0165	-0.0093	-0.0129
MLS	Maltese sarda	South Europe	-0.0131	-0.0033	-0.0082
ALP	Alpine suisse	South Europe	-0.0098	-0.0024	-0.0061
ORO	Orobica	South Europe	-0.0079	-0.0018	-0.00485
BEY	Bermeya	South Europe	-0.0053	-0.0005	-0.0029
SAAIT	Saanen Italy	South Europe	-0.0053	0.0011	-0.0021
MLG	Malaguena	South Europe	-0.0048	0.0019	-0.00145
ARG	Argentata	South Europe	0.0003	0.0062	0.00325
MUG	Murciano-Granadina	South Europe	-0.0006	0.0075	0.00345
ANG	Angora	South Europe	0.0006	0.0087	0.00465
PVC	Provencale	South Europe	0.0003	0.0107	0.0055
PTV	Poitevine	South Europe	0.008	0.0138	0.0109
FSS	Fosses	South Europe	0.0107	0.0166	0.01365
GGT	Girgentana	South Europe	0.0105	0.0172	0.01385
BIO	Bionda dell'Adamello	South Europe	0.0156	0.0227	0.01915
CRS	Corse	South Europe	0.0215	0.0264	0.02395
ASP	Aspromontana	South Europe	0.022	0.0288	0.0254

VSS	Valpassiria	South Europe	0.0301	0.0378	0.03395
CCG	Ciociara Grigia	South Europe	0.0335	0.0396	0.03655
RAS	Blanca de Rasquera	South Europe	0.0342	0.0413	0.03775
NIC	Nicastrese	South Europe	0.0352	0.0422	0.0387
VAL	Valdostana	South Europe	0.037	0.0438	0.0404
PYR	Pyrenean	South Europe	0.04	0.0465	0.04325
MAL	Mallorquina	South Europe	0.0405	0.0481	0.0443
MLT	Maltese	South Europe	0.0421	0.0513	0.0467
ARR	Traditional Arran	North Europe	0.0293	0.0413	0.0353
ICL	Icelandic Goat	North Europe	0.0395	0.0552	0.04735
LNRFI	Landrace Goat	North Europe	0.0294	0.0376	0.0335
LNRDK	Landrace Goat	North Europe	-0.0502	-0.0432	-0.0467
BLB	Bilberry	North Europe	-0.0374	-0.0261	-0.03175
OIG	Old Irish Goat	North Europe	-0.0166	-0.0085	-0.01255
LNRNL	Landrace Goat	North Europe	-0.0255	-0.0157	-0.0206
OIGX	Old Irish goat cross	North Europe	0.16	0.1732	0.1666

Mean F_{IS} by population, 95% confidence interval upper and lower bounds.
